# Platelet transfusion practice in the intensive care unit: the Nine-I international platelet transfusion survey

**DOI:** 10.1186/s13613-025-01494-4

**Published:** 2025-07-08

**Authors:** Lene Russell, Elie Azoulay, Carl Thomas Anthon, Frédéric Pène, Padmastuti Akella, Asma Mabrouki, Kathryn Puxty, Lene Bjerregaard Nielsen, Jo Bønding Andreasen, Thomas Kander, Fredrik Sjövall, Johanna Hästbacka, Christine Lodberg Hvas, Andry Van De Louw, Sanjay Chawla, Philippe R. Bauer, Pedro Castro, Pedro Povoa, Luis Coelho, Sara Fernandez, Arzu Topeli, Andreas Barratt-Due, Caterina Barbaglio, Matthias Kochanek, Ignacio Martin-Loeches, Nancy Kentish-Barnes

**Affiliations:** 1 Present Address: Department of Intensive Care, Copenhagen University Hospital Gentofte, Gentofte Hospitalsvej 1, 2900 Hellerup, Denmark; 2 Department of Clinical Medicine, University of Copenhagen, Copenhagen, Denmark; 3 Médecine Intensive & Réanimation, Groupe Famiréa, Hôpital Saint-Louis, Assistance Publique – Hôpitaux de Paris, Université Paris Cité, Paris, France; 4 Department of Intensive Care, Copenhagen University Hospital – Rigshospitalet, Copenhagen, Denmark; 5 Médecine Intensive & Réanimation, Hôpital Cochin, Assistance Publique – Hôpitaux de Paris, Institut Cochin, INSERM U1016, CNRS UMR8104, Université Paris Cité, Paris, France; 6 Division of Pulmonary, Critical Care and Sleep Medicine, University of South Florida, Tampa, FL USA; 7 Department of Intensive Care, Glasgow Royal Infirmary, Glasgow, UK; 8 Department of Anaesthesiology and Intensive Care, Odense University Hospital, Odense, Denmark; 9 Department of Anaesthesia and Intensive Care, Aalborg University Hospital, Aalborg, Denmark; 10 Department of Intensive and Perioperative Care, Skåne University Hospital, Lund, Sweden; 11 Department of Clinical Sciences, Lund University, Lund, Sweden; 12 Department of Intensive Care, Tampere University Hospital, Wellbeing Services County of Pirkanmaa and Tampere University, Tampere, Finland; 13 Department of Anaesthesiology and Intensive Care, Aarhus University Hospital, Aarhus, Denmark; 14 Division of Pulmonary and Critical Care, Penn State University College of Medicine, Hershey, PA USA; 15 Critical Care Medicine Service, Department of Anesthesiology and Critical Care Medicine, Memorial Sloan Kettering Cancer Center, New York, NY USA; 16 Division of Pulmonary and Critical Care Medicine, Mayo Clinic College of Medicine and Science, Rochester, MN USA; 17 Medical Intensive Care Unit, Hospital Clinic Barcelona, IDIBAPS, University of Barcelona, Barcelona, Spain; 18 Department of Intensive Care, Hospital de Sao Francisco Xavierl, ULSLO, Lisbon, Portugal; 19 NOVA Medical School, CHRC, NOVA University of Lisbon, Lisbon, Portugal; 20 Center for Clinical Epidemiology and Research Unit of Clinical Epidemiology, Odense University Hospital, Odense, Denmark; 21 Department of Internal Medicine, Division of Intensive Care, Faculty of Medicine, Hacettepe University, Ankara, Turkey; 22 Department of Anaesthesia and Intensive Care Medicine, Division of Emergencies and Critical Care, Oslo University Hospital, Rikshospitalet, Oslo, Norway; 23 Department of Transplant Anaesthesia and Intensive Care, Great Metropolitan Hospital Niguarda, Milan, Italy; 24 Department I of Internal Medicine, Faculty of Medicine, University of Cologne and University Hospital Cologne, Cologne, Germany; 25 Department of Intensive Care Medicine, Multidisciplinary Intensive Care Research Organisation (MICRO), St James’s Hospital, Dublin, Ireland; 26 CIBER of Respiratory Diseases (CIBERES), Institute of Health Carlos III, Madrid, Spain; 27 Faculty of Medicine and Health Technology, Tampere University, Tampere, Finland; 28 Department of Clinical Medicine, Aarhus University, Aarhus, Denmark; 29 Department of Anesthesiology, Weill Cornell Medical College, New York, USA

**Keywords:** Platelet transfusions, Thrombocytopenia, Critically ill, Intensive care

## Abstract

**Background:**

Platelet transfusions are frequent in the Intensive Care Unit (ICU), either as prophylaxis against bleeding complications or as treatment for bleeding. The European Society of Intensive Care Medicine guidelines for ICU patients generally recommend not using prophylactic platelet transfusions unless the platelet count falls below 10 × 10^9^ cells/L in non-bleeding patients and make no recommendation for platelet transfusion threshold in non-massively bleeding patients with thrombocytopenia. Therefore, the decision to transfuse platelets is often left to clinical assessment by the treating physician. This study aims to describe current platelet transfusion preferences among ICU physicians.

**Methods:**

An online, anonymous survey consisting of 43 items was produced in two languages (French and English) and distributed by investigators in the Nine-I research network to ICU physicians in Europe and the United States of America. The survey evaluated platelet transfusion practices in ICU patients with and without bleeding, the presence of local guidelines, and factors influencing the decisions to transfuse platelets. Only completed surveys were analysed.

**Results:**

We received 997 surveys completed by ICU physicians. Overall, there was large heterogeneity in platelet transfusion practices between and within countries. In non-bleeding, thrombocytopenic medical ICU patients, most would transfuse prophylactic platelets at a platelet count threshold of 10 × 10^9^ cells/L. Thirty percent would change their strategy in patients with bone marrow failure and either be more liberal (60%; 95% Confidence Limits 0.54, 0.66), more restrictive (31%; 0.26,0.36) or seek assistance. Higher thresholds were preferred in surgical patients, prior to procedures and in patients with bleeding. Only 173 (17%; 0.15,0.19) responded that they were confident about the clinical indications every time they prescribed a platelet transfusion. As for existing guidelines, only 123 (12%; 0.10,0.15) responded that they always read them. Colleagues' attitudes and departmental culture were important influencers on transfusion practice.

**Conclusion:**

Platelet transfusion practice in the ICU is heterogeneous, both between and within countries; guidelines are often not used, and there is often uncertainty about the clinical indication.

**Supplementary Information:**

The online version contains supplementary material available at 10.1186/s13613-025-01494-4.

## Background

Thrombocytopenia is common in the intensive care unit (ICU) [1–4] and is seen in several different subpopulations, such as trauma patients [5], cardiac ICU patients [6, 7], patients with sepsis [8] and patients with cancer [9, 10]. Therefore, the pathogenesis behind thrombocytopenia may differ among patients. Moreover, while associated with worse outcomes in most studies, the impact of thrombocytopenia on outcomes has not been established [11].

Thrombocytopenia increases the risk of major bleeding complications [12] and platelet transfusions are often used in ICU patients [13] to prevent or treat bleeding [14, 15]. The European Society of Intensive Care Medicine guidelines generally recommend not using platelet transfusions unless the platelet count falls below 10 × 10^9^ cells/L in non-bleeding patients, make no recommendation regarding prophylactic platelet transfusion prior to invasive procedures for platelet counts between 10 and 50 × 10 cells/L [16] and make no recommendation for the use of a restrictive versus a liberal platelet transfusion threshold in non-massively bleeding patients with thrombocytopenia [17]. Therefore, the decision to transfuse platelets is often left to the clinical assessment by the treating physician.

This study aimed to assess ICU physicians'preferred platelet transfusion practices and explore which variables influence their decisions. We hypothesised that there would be a substantial heterogeneity in platelet transfusion practice and that existing guidelines were not always followed.

## Methods

### Questionnaire design

A questionnaire consisting of 7 sections and 43 questions was constructed by LR and NKB. The sections addressed the following topics: (1) Hospital and ICU structure, (2) Institutional protocols and guidelines, (3) Preferences in the use of prophylactic platelet transfusions in different clinical scenarios or prior to procedures, (4) Platelet transfusions in bleeding, (5) Participants’ views on future platelet transfusion trials, (6) Views and concerns about transfusions and (7) Participant characteristics.

The questionnaire was constructed in two languages. The first version was written in English and then translated into French. Adequate translation was performed by a professional writer fluent in both languages, and medical consistency was verified by a French-speaking physician. The results were translated from French to English by two French–English-speaking physicians. Both questionnaire versions were tested by the study team locally in Saint Louis Hospital, Paris, France, and Copenhagen University Hospital Gentofte, Denmark, prior to distribution. We used the Consensus-Based Checklist for Reporting Survey Studies (CROSS) when designing and reporting the study [18].

### Distribution

The survey was distributed through the ‘Caring for Critically Ill Immunocompromised Patients International research network (Nine-I), where investigators distributed the internet-based questionnaire (UmfrageOnline, Enuvo, Switzerland) in their respective countries. The survey was distributed to approximately 4000 physicians working in adult ICUs in 15 countries from the 1 st of April to the 30th of September 2023, either through direct email or, as in Portugal and Sweden, through the societies. The English version of the survey was distributed to physicians in Denmark, the United Kingdom (UK), Finland, Germany, Ireland, Italy, Norway, Portugal, Spain, Sweden, the Netherlands, Türkiye and the United States of America (USA). The French version was distributed to physicians located in France and Belgium. Up to three reminders were sent before database closure. Details about distribution can be found in Supplement 1, Figure S1 and Table S1. The cover letter, full surveys and information about the Nine-I research network can be found in Supplement 2.

### Statistics

Based on the assumption that ICU physicians can be viewed as a homogenous sample, we calculated a sample size of approximately 400 respondents in the final analysis to achieve a margin of error of 5% and a confidence level of 95%; and 660 respondents in the final analysis to achieve a confidence level of 99%. Data are presented descriptively with continuous variables as medians with interquartile ranges (IQRs) and categorical variables as numbers and percentages with Wilson score 95% confidence limits. Countries with fewer than 20 responses were pooled in the per-country analyses. Open-ended responses and comments in sections 3 and 6 were analysed using thematic analysis, where an initial code book consisting of key themes was identified. The data were then categorised using the initial codes through an iterative process, after which the final themes were confirmed.

Only completed surveys were analysed. SAS Enterprise Edition 3.8, SAS Institute Inc. was used for quantitative statistical analyses.

## Results

### Response rates and completed surveys

We received 1384 survey responses, of which 999 were completed; 825 completed the English version of the survey, and 174 completed the French version. Two responses were excluded (non-ICU physicians), leaving 997 responses for final analyses. The estimated response rate was 30–35% and the completion rate 72%. Response and completion rates varied across countries (Supplement, Table S1 and Fig. S2).

### Demographics

Most survey respondents worked in publicly funded ICUs within not-for-profit University Hospitals, and their primary specialties were intensive care medicine, anaesthesiology, and internal medicine. Demographics are shown in Table 1; details per country can be found in Supplement 1, Table S2.

**Table 1:** Demographics

Participants demographics		
Age	42	36–50
Gender:		
Female	390	39.1%
Male	589	59.1%
Non-binary and other	3	0.3%
Prefer not to answer	15	1.50%
ICU experience (years):	10	4–16
Primary speciality:		
Intensive care medicine^a)^	568	57.0%
Anaesthesiology	304	30.5%
Internal medicine	71	7.1%
Pulmonology	19	1.9%
Haematology	7	0.7%
Gasteroenterology and/or hepatology	7	0.7%
Surgery	7	0.7%
Other specialty^b)^	15	1.5%
ICU demographics		
Number of ICU beds:		
Less than 10	221	22.2%
10–19	312	31.3%
20–29	292	29.3%
30–39	63	6.3%
40 or more	109	10.9%
Type of institution:		
University hospital	736	73.8%
University-affiliated hospital	208	20.9%
Regional non-teaching hospital	53	5.3%
Hospital funding:		
Public	841	84.4%
Private	52	5.2%
Mixed	98	9.8%
Other ^c)^	6	0.6%
Presence of haematology department in hospital:	776	77.8%
Presence of oncology department in hospital	813	81.5%
Type of patients treated in the ICU:		
Medical patients	941	94.4%
Surgical patients (including trauma)	784	78.6%
Neurosurgery patients	334	33.5%
Cardio-thoracic surgery patients	283	28.4%
Haematological patients	729	73.1%
Oncological patients	767	76.9%
Burn patients	178	17.9%
Solid organ transplant patients	411	41.2%
Haematopoetic stem cell transplant patients	415	41.6%
ECMO/ECLS patients	327	32.8%
Presence of platelet transfusion protocol in the hospital:		
Yes	422	42.3%
No	342	34.3%
I do not know	233	23.4%
Presence of an ICU specific platelet transfusion protocol		
Yes	139	13.9%
No	698	70.0%
I do not know	160	16.1%
Who can prescribe a platelet transfusion at your ICU?		
ICU specialist	979	98.2%
Haematologist	450	45.1%
Transfusion medicine specialist	136	13.6%
Resident, trainee, junior doctor	773	77.5%
Nurse	6	0.6%
Other specialists ^d)^	38	3.8%

Data are presented as number (percentages) or median (interquartile range(IQR))

*ICU* Intensive Care Unit, *ECMO* Extracorporeal membrane oxygenation, *ECLS* Extracorporal life support

^a)^16 physicians responded that they were specialists in Intensive Care Medicine and Anaesthesiology (15) or Intensive Care Medicine and pulmonology. (1) Here they are listed as ICM specialists only

^b)^Other specialties included neurology, oncology, pediatric critical care, emergency medicine, infectious diseases specialists, cardiologists, nephrologists and burn specialists

^c)^Included hospitals owned by foundations, churches and religious organisations

^d)^Other specialists who can prescribe platelet transfusions include surgeons, internal medicine specialists, cardiologists

### Confidence in the decision to transfuse platelets and the use of guidelines

Survey participants were asked, “The last ten times you provided patients with platelet transfusions, how many times were you confident about the clinical indication?”. Only 173 (17%; 95% CI 0.15,0.19) responded that they had been confident with the decision every single time. 633 (63%; 0.60,0.66) were confident “most” times, whereas the remaining 191 respondents (19%; 0.17,0.22) were confident about their decision only half of the time or less. These results were consistent throughout all countries, and no difference was found between gender, ICU experience or age.

**Fig. 1:**
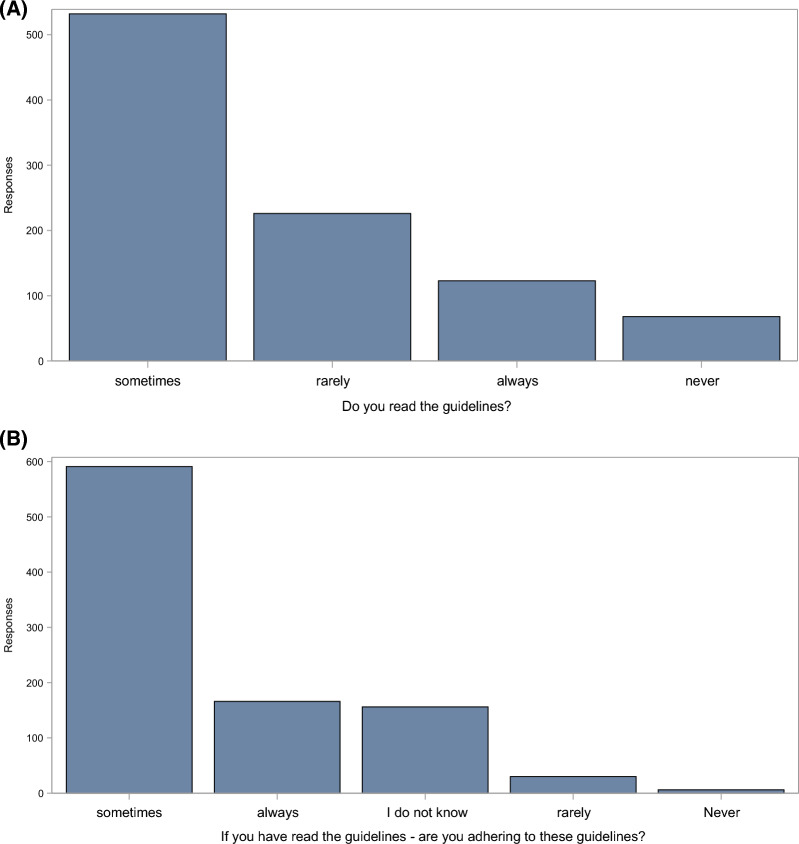
Use and adherence to guidelines. **A** show the number of physicians who responded that they read the guidelines about platelet transfusions. Most (532, 53%; 95% CL 0.50–0.56) said they read the guidelines sometimes, and 123 (12%; 0.10,0.15) said they always read them. Alternatively, 226 (23%; 0.20,0.25) and 68 (7%; 0.05,0.09) said they rarely or never read the guidelines. In **B** the replies to the questions: ‘*If you have read the guidelines, do you adhere to the guidelines?’* are shown. Here, the majority (591(62%; 0.59,0.65) replied that they ‘sometimes’ adhere to the guidelines, whereas 166 (17%; 0.15,0.20) said they always adhered if they had chosen to read them. The remaining 192 (20%) responded that they never or rarely adhered to the guidelines or replied that they did not know. (Please note that 48 respondents who replied ‘I do not know’ to Question **A** are not shown in the figure **B**)

As for existing guidelines, only 123 (12%; 0.10,0.15) said they always read the guidelines, and even if they had read them, only 167 (17%; 0.15,0.19) said they always followed the guidelines (Fig. 1). Here, there was some difference between countries (Supplement 1, Table S3 and Fig. S3).

### Prophylactic platelet transfusions in medical patients with thrombocytopenia and no bleeding

**Fig. 2:**
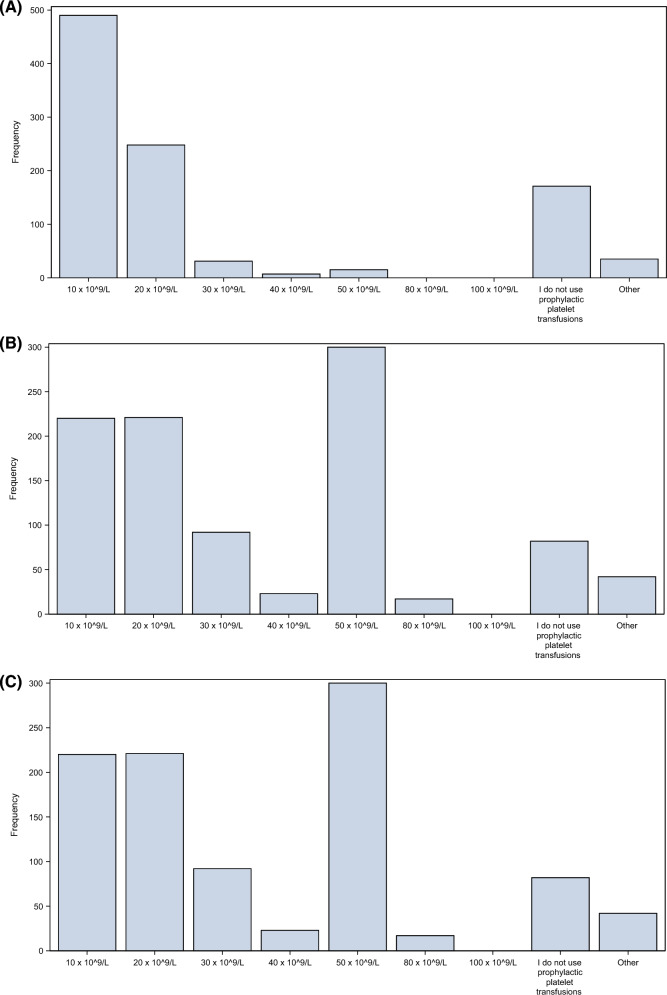

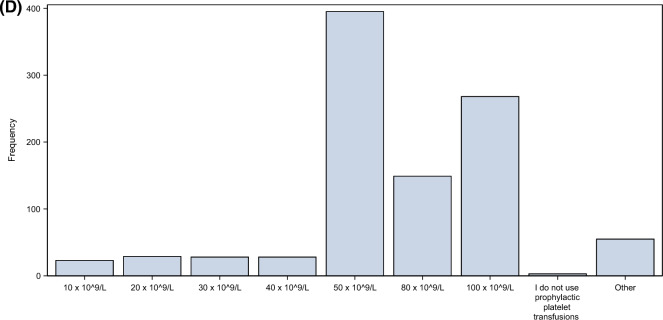
Platelet transfusion thresholds. **A** Prophylactic platelet transfusion threshold in medical patients (without bleeding). **B** Prophylactic platelet transfusion threshold in surgical patients (without bleeding). **C** Platelet transfusion threshold in patients with minor bleedings (WHO I–II; not requiring RBC transfusion). **D** Platelet transfusion threshold in patients with MAJOR bleeding (WHO III–VI; requiring RBC transfusion)

In non-bleeding, thrombocytopenic *medical* ICU patients, 490 (49%; 0.46,0.52) of the respondents would transfuse prophylactic platelets at a threshold of 10 × 10^9^ cells/L, and 248 (25%; 0.22,0.28) would use a threshold of 20 × 10^9^ cells/L. 171 (17%; 0.15,0.20) respondents answered that they would not use prophylactic platelet transfusions at all (Fig. 2A).

There were some differences between countries. For example, respondents from the USA were more likely to prefer a threshold of 10 × 10^9^ cells/L (78%; 0.69,0.85), whereas the French and UK physicians were more likely to prefer a threshold of 20 × 10^9^ cells/L (45%; 0.38,0.52 and 46%; 0.38,0.58, respectively) (Supplement 1; Table S4 and Fig. S4).

More than half, (605 (61%; 0.58,0.64)), responded that they always checked the platelet increment after giving one platelet transfusion before giving a second platelet transfusion, 231 (23%,0.21,0.26) said they did so sometimes, whereas the last 161(16%, 0.14, 0.19) responded that they did not check.

Among the 776 physicians who treated haematological patients in their ICUs, 120 (16%; 0.14, 0.19) said they did not use prophylactic transfusions.

However, when asked if the presence of *bone marrow failure* would change their management, 292 (29%; 0.27,0.32) of the respondents answered ‘yes, it would’. Of these, 175 (60%; 0.54,0.66) responded that they would be more *liberal* with transfusions. Three main themes were identified among explanations for a more liberal strategy: (1) A perceived higher risk of severe thrombocytopenia, (2) A perceived higher risk of bleeding, and (3) The perceived presence of dysfunctional platelets. In addition, several responded that the haematologists in their hospitals insisted on higher transfusion thresholds. Relevant quotes can be found in Supplement 1, Table S5A.

On the contrary, 90 respondents (31%; 0.26,0.36) said they would be more restrictive in this scenario. Three main themes were identified explaining why a more restrictive transfusion strategy would be favoured in patients with bone marrow failure: (1) The transfusion will not be effective, (2) Fear of alloimmunisation and (3) A perceived higher tolerance of thrombocytopenia (Supplement 1, Table S5B).

### Conditions and treatments influencing the decision to transfuse platelets in patients without bleeding

**Fig. 3:**
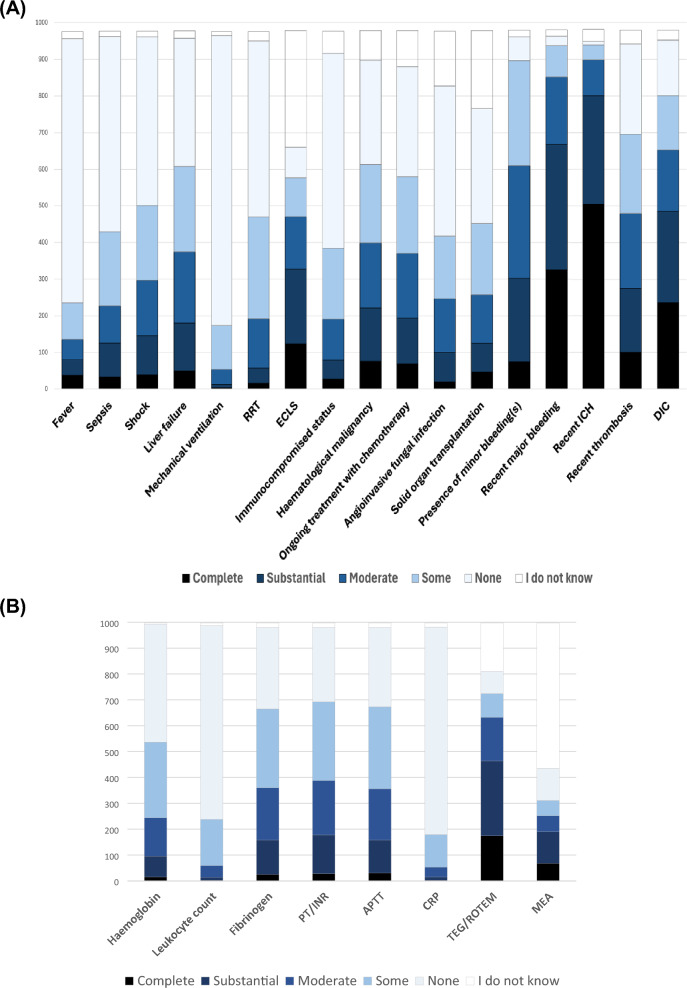
**A** Conditions and treatments influencing the decision to transfuse platelets in thrombocytopenic patients without bleeding. The survey participants were asked to answer to what extent the conditions and treatments shown in the figure would influence their decision and make it more likely to transfuse. As shown in the figure, recent intracranial haemorrhage and recent major bleeding were strong influencers making it more likely for the respondents to use prophylactic platelet transfusions, whereas fever, sepsis and shock did not strongly influence the decision of most responders. *DIC* disseminated intravascular coagulation, *ECLS* extracoporeal life support, *ICH* intracranial haemorrhage, *RRT* renal replacement therapy. **B** Blood tests influence on the decision to transfuse platelets in thrombocytopenic patients without bleeding. The survey participants were asked to answer to what extent the biomarkers and coagulation test shown in the figure would influence their decision and make it *more likely* for them to transfuse. Overall, more than half of the survey respondents indicated that the viscoelastic tests TEG and ROTEM would have an influence on their decision to transfuse. *APTT* activated partial thromboplastin time, *CRP* C-reactive protein, *PT* Prothrombin time, *INR* International normalised ratio, *TEG* Thromboelastography, *ROTEM* Rotational thromboelastometry, (INR), *MEA* Multiple electrode aggregometry (e.g. Multiplate analyser)

Most physicians would be more likely to use prophylactic platelet transfusions in non-bleeding patients with recent major bleeding or intracerebral haemorrhage (ICH). Disseminated intravascular coagulation (DIC), presence of minor bleedings and extracorporeal membrane oxygenation (ECMO) treatment were other conditions that many responded would make it more likely to use a prophylactic platelet transfusion (Fig. 3A). Results of other blood tests than the platelet count also influenced the decision to transfuse or not (Fig. 3B). Here, substantial differences between countries were found; for example, 71% of Danish physicians responded that the result of viscoelastic analyses such as thromboelastography (TEG) and rotational thromboelastometry (ROTEM) had a strong or complete influence on their decision-making, compared to 16% of French physicians (Supplement 1, Fig S5A-D).

### Prophylactic platelet transfusions in surgical patients and prior to procedures

**Fig. 4:**
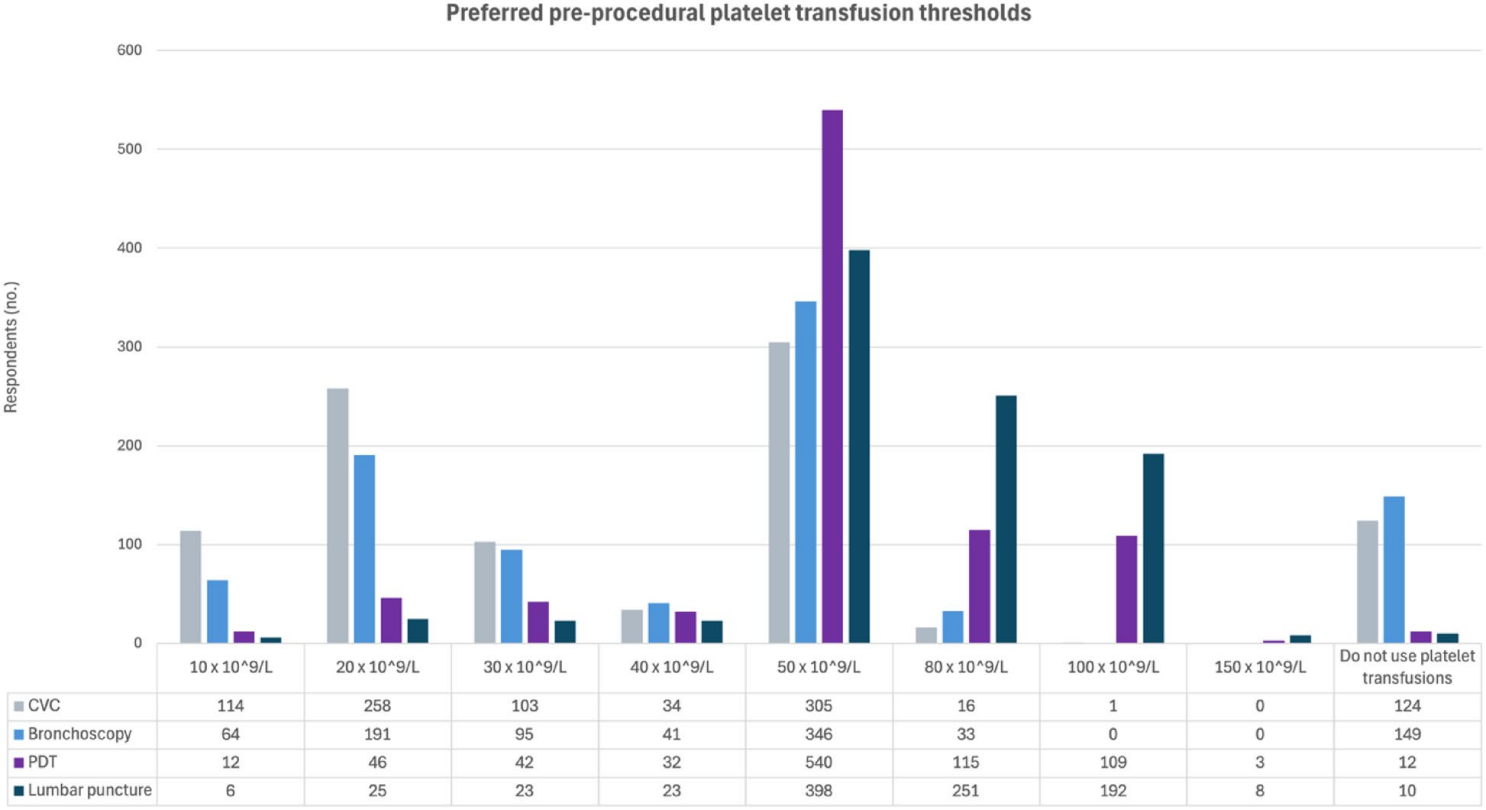
Preferred prophylactic platelet thresholds prior to different invasive procedures in the ICU. Overall, for all procedures, the most preferred threshold was 50 × 10^9^ cells/litre, preferred by 305 (31%) prior to placing a CVC, 346 (35%) prior to a bronchoscopy, 540 (54%) prior to a PDT and 398 (40%) prior to a lumbar puncture. More details can be found in Supplement 1, Tables and Figures S7–S10. *CVC* central venous catheter, *PDT* percutaneous dilatation tracheostomy

In non-bleeding, thrombocytopenic, surgical and trauma ICU patients, the most preferred threshold was 50 × 10^9^ cells/L (300 respondents, 30%; 0.27, 0.33) (Fig. 2B and Supplement 1: Table S6 and Fig S6). The preferred platelet counts prior to procedures varied considerably, but overall, most preferred a threshold of 50 × 10^9^ cells (Fig. 4). However, prior to placing a central venous catheter or performing a bronchoscopy, 124 (12%; 0.11,0.15) and 149 (15%; 0.13,0.17), respectively, would not transfuse at all (Supplement 1: Tables S7-S10, Figures S7-S10).

### Platelet transfusions in patients with bleeding

In thrombocytopenic ICU patients with *minor* bleeding (World Health Organisation Bleeding Classification (WHO) I and II; classified as non-CNS bleeding not requiring RBC transfusions) [19], 350 (35%; 0.32,0.38) would prefer a threshold of 50 × 10^9^ cells/L and 276 (28%; 0.25,0.30) a threshold of 20 × 10^9^ cells/L (Fig. 2C, Supplement 1: Table S11 and Fig. S11).

In thrombocytopenic ICU patients with *major* bleeding (WHO III and IV; classified as bleeding requiring RBC transfusions or CNS bleeding), 395 (40%; 0.37,0.43) responded that they would prefer a threshold of 50 × 10^9^ cells/L and 268 (27%; 0.24,0.30) a threshold of 100 × 10^9^ cells/L. 55 (6%) responded that they did not use a specific platelet count in patients with major bleeding but balanced transfusion or a transfusion strategy depending on the clinical context (Fig. 2D and Supplement 1: Table S12 and Fig S12).

**Fig. 5:**
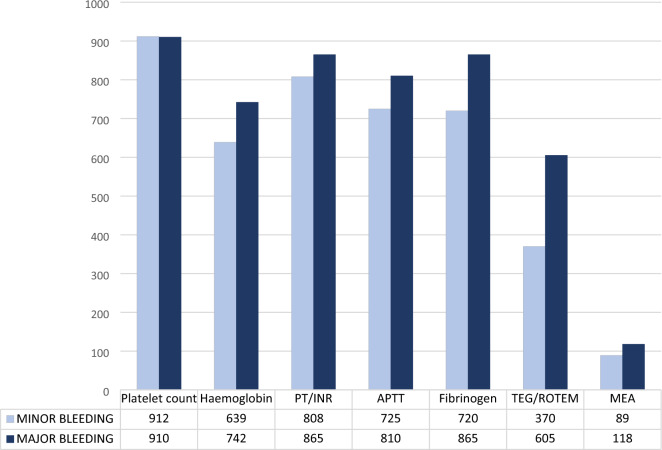
Use of coagulation tests to evaluate coagulation in thrombocytopenic patients with bleeding. Most respondents would use standard coagulation tests to evaluate coagulation in thrombocytopenic patients with bleeding. A minority said they used MEA; here 67/89(75%) and 79/118(67%) were from Denmark. (More details can be found in Supplement 1, Tables S13-14 and Figures S13-S19.). *APTT* activated partial thromboplastin time, *CRP* C-reactive protein, *PT* Prothrombin time, *INR* International normalised ratio, *TEG* Thromboelastography, *ROTEM* Rotational thromboelastometry, (INR), *MEA* Multiple electrode aggregometry (e.g. Multiplate analyser)

Respondents in most countries would use standard coagulation tests to evaluate coagulation in a thrombocytopenic patient with minor and major bleeding (Fig. 5). Denmark was the exception; here, viscoelastic analyses were preferred by 37% (0.34,0.40) in minor and 60% (0.58,0.64) in major bleeding, respectively (Supplement 1: Table S13-14 and Figures S13-19).

### Specific properties of blood products and dilemmas with transfusion

About half of the respondents [481 (48%;0.45,0.51)] considered blood products to be different from other treatments they prescribed to their patients. The most common explanation was that blood products are a ‘rare resource with a limited supply’ [128 (27%)], followed by the answer that it is ‘living material’ [100 (21%)]; here, the phrase “transplantation” was used by several respondents. Relevant quotes can be found in Supplement 1, Table S15.

The majority of responded that it was likely, possible, or that they could not rule out that unknown elements could be transferred to patients through blood products. Only 173 (17%; 0.15, 0.20) were convinced this was impossible. The most common concerns were unknown infections [125 (13%)] and the transfer of other unknown substances, including immunologically active compounds, chemicals, toxins, and drugs (Relevant quotes can be found in Supplement 1: Table S16).

As for their views and practices on blood products, 604 (61%; 0.58,0.64) believed that social factors could affect their views. When asked what they thought was most influential, the most common responses were the culture in the institution, colleagues’ views, religion (primarily if the patient was religious), availability of blood products, opinions from specialists and previous experiences and fear of consequences. Relevant quotes can be found in Supplement 1, Table S17.

### Participation in future randomised trials

Overall, most respondents supported the idea of a future randomised controlled trial involving patients with thrombocytopenia: 883 (89%; 0.86,0.90) answered that they would be or were likely to randomise this population to different management strategies. Most [676 (68%; 0.65,0.70)] favoured a three-level protocol with three different transfusion thresholds depending on the risk of bleeding: one for low (or normal), one for high risk and one for ongoing bleeding. However, 126 (13%,0.11,0.15) responded that they would not be willing to include patients with ongoing bleeding in a trial (Supplement 1: Table S20-S22, Fig S20-22).

## Discussion

The results of this large survey, with participants from 15 countries, illustrate the considerable heterogeneity in platelet transfusion practice in critically ill patients between countries and within countries. Variations were seen in the preferred thresholds, in which conditions triggered transfusion and in preferred blood tests to evaluate the need for transfusion. The considerable variation in practice likely reflects the uncertainty many physicians experience when prescribing platelet transfusions; only 17% of physicians in this survey responded that they were confident about the clinical indication every time.

Other surveys on platelet transfusion have previously been performed. The TRACE survey from 2018 evaluated blood product transfusion practices in ICU patients without bleeding, including platelet transfusions, among 745 respondents worldwide [20]. They found that in patients without a planned invasive procedure, respondents would transfuse patients at a median platelet count of 20 (Interquartile range (IQR) 10–25) × 10^9^ cells/L. Higher transfusion triggers were used prior to invasive procedures. Another smaller survey (n = 97) investigated the use of platelet transfusions before placement of central venous catheters and found current transfusion practice highly variable and the decision to be based mainly on clinical parameters, insertion site and technique applied [21]. The TRACE-2 survey from 2022 evaluated transfusion practices in ICU patients with bleeding and found, similar to our study, that the applied platelet threshold for the general non-massively bleeding ICU patient was 50 (IQR 20–50) × 10^9^/L [22]. Outside the ICU setting, a ﻿recent survey among Dutch haematologists (n = 73) focusing on thrombocytopenia in outpatients with acute leukaemia, myelodysplastic syndrome and aplastic anaemia found that a threshold of ≤ 10 × 10^9^ cells/L was routinely used; however, this changed if clinical conditions that potentially could increase bleeding risks were present in which a wide range of thresholds between 10 and 50 × 10^9^ cells/L were applied [23]. Similarly, this study found substantial heterogeneity in how clinicians evaluate bleeding risk in various clinical conditions.

It was clear from our study that the presence of *bone marrow failure* influenced management, in most cases towards a more liberal transfusion strategy. This may be surprising given the results of large trials in haematology patients, which have generally shown that a restrictive strategy is safe [15, 24]. However, studies in haematological ICU patients have demonstrated that the risk of bleeding is increased in critically ill haematology patients, likely due to severe inflammation and sepsis [8, 25].

One surprising finding in our survey, considering that previous studies on platelet transfusion response in ICU patients typically use a platelet count measured 18–24 h post-transfusion [26, 27], was that 61% responded that they always checked the platelet count after administering one transfusion before giving a second platelet transfusion in patients without bleeding.

Given the lack of evidence for such practice, another unexpected finding was the high number of physicians who reported using viscoelastic methods such as TEG/ROTEM in thrombocytopenic ICU patients with or without bleeding [28, 29]. This lack of evidence may also explain the large difference between countries; for example, whereas the majority of Danish physicians responded that they used TEG/ROTEM for evaluating coagulation in patients with minor bleeding, the opposite was true in France.

Several societal guidelines about platelet transfusions have been published in the last decade, although few are specifically aimed at ICU patients. More importantly, they are not entirely consistent in their recommendations. The American Society of Clinical Oncology clinical practice guidelines recommend prophylactic platelet transfusion for platelet counts < 10 × 10^9^ cells/L in patients receiving active therapy for cancer and those undergoing allogeneic hematopoietic stem cell transplantation (HSCT). In cancer patients not receiving active chemotherapy but who have chronic, stable, but severe thrombocytopenia, observation has been a recommended strategy [30]. On the other hand, the Association for the Advancement of Blood and Biotherapies (AABB) 2015 guidelines strongly recommend prophylactic platelet transfusion for most hospitalised patients with a platelet count below 10 × 10^9^ cells/L to prevent spontaneous bleeding [31]. The British Society for Haematology 2017 guidelines suggest that thresholds recommended for reversible bone marrow failure may be used as a general guide also for non-bleeding patients with critical illness; also here, prophylactic platelet transfusions to maintain a platelet count at or above 10 × 10^9^ cells/L is recommended, however, in patients judged to have additional risk factors for bleeding it should be considered to raise the threshold to between 10 and 20 × 10^9^ cells/L. Here, “individual review is required”. They also provide more specific recommendations for peri-procedural platelet count threshold, e.g. > 20 × 10^9^cells/L for inserting central lines [32]. Meanwhile, the European Society of Intensive Care Medicine guidelines from 2020 suggest not using platelet transfusions to treat thrombocytopenia unless the platelet count falls below 10 × 10^9^/L and make no recommendation regarding prophylactic platelet transfusions before invasive procedures when counts are between 10 and 50 × 10^9^ cells/L [16].

This study confirms findings from previous studies indicating that physicians often do not find the guidelines helpful in their daily practice [33, 34]. In this survey, only a minority said they always read the guidelines and followed them if they had chosen to read them. However, we did not explore when or how often the participants read the guidelines. In the TRACE survey, no association was found between the presence of a local guideline and transfusion practice [20]. Previous studies have also shown that guidelines concerning procedures with higher complexity have lower compliance rates than those low on complexity [35]. Prophylactic platelet transfusion in critically ill patients indeed falls under the first category; the bleeding risk may be reduced, but the effects are likely depending on both acute factors relating to critical illness and the underlying condition [36]. Furthermore, the risk of procedure-related bleeding complications in thrombocytopenic ICU patients remains unclear, and there is limited evidence that pre-procedural platelet transfusion reduces the risk of bleeding in ICU patients [37]. Overall, the very large number of guidelines produced per year (> 1000) is probably also part of the reason, which, in a broad field like intensive care medicine, makes it challenging to keep up. Also, because clinical practice guidelines specify how health care should be performed, they could be considered a threat to clinical autonomy [38], which could potentially be harder to tolerate when guidelines are not based on solid evidence. Physicians believing that following the guideline will not lead to the desired outcome have been described as a reason why guidelines are often not followed [34].

This survey also demonstrated that many physicians have concerns about the side effects of transfusions and would favour a restrictive strategy. This is understandable, as RCTs in preterm infants [39, 40], in patients with ICH [41] and patients with dengue infection [42] have indicated both short-term and long-term harm from platelet transfusions, as have various smaller observational studies in ICU patients [43–45] and cancer patients [46]. For the physicians in this study, the most common concern was the transfer of infections, but other potential side effects were also mentioned, such as the transfer of immunologically active compounds and harmful chemicals. This study also highlights the importance of departmental and societal culture on physicians’ decision-making. That colleagues'attitudes are important influencers for transfusion practice has also recently been confirmed in a study on intraoperative transfusion decision-making variability, where physicians reported fear of being judged by peers, nursing staff, and patients if making the wrong decision [47]. Furthermore, as acknowledged by the participants in this survey, platelets are a valuable resource, and shortages are common.

To our knowledge, this is the largest international survey on platelet transfusion practice among ICU physicians. As surveys are the principal method used to address topics about attitudes or opinions which cannot be assessed using other approaches [48], we used this method to collect information on intensivists’ practices, preferences and beliefs on platelet transfusions in a sample that we believe is large enough to encompass the heterogeneity of platelet transfusion practices in the participating countries. Furthermore, we produced the survey in both English and French to minimize language barriers as much as possible.

This study also has several limitations. The response rate was low, and the survey population was unbalanced between countries. The survey was quite substantial, and many questions required fluency in English or French at a higher level. As the number of participants completing the survey varied considerably between countries, this could indicate a variation in English proficiency, which may have restricted some from completing the survey. Also, most clinicians in this study worked in university hospitals, likely because many were recruited through personal emails by investigators or through research networks. Our results may, therefore, mainly mirror physicians working in academic settings. Furthermore, surveys record opinions and not actual daily practice; if this had been the case, the results may have been different.

## Conclusion

Platelet transfusion practice in the ICU is heterogeneous, but overall, most favour a restrictive strategy in patients without bleeding. Clinicians are often not sure about the clinical indication, and the use of existing guidelines is limited. Many physicians are concerned about the side effects of transfusions, and colleagues'attitudes are important influencers of transfusion practice. Future high-evidence studies on platelet transfusions are urgently needed, and most respondents in this survey would be happy to enrol patients into a randomized controlled trial.

## Supplementary Information

Supplementary material 1.

Supplementary material 2.

## Data Availability

The data sets used and/or analysed in the current study are available from the corresponding author upon reasonable request.
