# Supplementary material for: Platelet transfusion practice in the intensive care unit: the Nine-I international platelet transfusion survey
Source: Ann Intensive Care. 2025 Jul 8;15:91. doi: 10.1186/s13613-025-01494-4 (PMC12237844; doi:10.1186/s13613-025-01494-4)
Supplement: Supplementary file 1 — Supplementary material 1. [file 13613_2025_1494_MOESM1_ESM.pdf]

SUPPLEMENTAL MATERIAL 1 – ADDITIONAL RESULTS

1. Countries in the PLOT ICU survey p. 2
2. Response rate and completed surveys per country p. 3
3. Demographics listed per country p. 5
4. Use of guidelines per country p. 8
5. Prophylactic platelet transfusions in medical patients per country p. 9
6. Results of qualitative analysis of reasons for strategy change in patients with bone marrow failure p. 10
7. Influence on coagulation analyses other than platelet count on the decision to transfuse prophylactic platelets p. 11
8. Prophylactic platelet transfusions in surgical patients per country p. 14
9. Preferred pre-procedural platelet counts p. 15
10. Transfusion thresholds in patients with minor bleeding p. 19
11. Transfusion thresholds in patients with major bleeding p. 20
12. Use of coagulation blood test to evaluate coagulation in thrombocytopenic patients with bleeding p. 21
13. Qualitative analysis of comments from the section on specific properties and dilemmas with transfusion of blood products p. 29
14. RCT preferences p. 33

Fig S1: Countries included in the Nine-I platelet survey

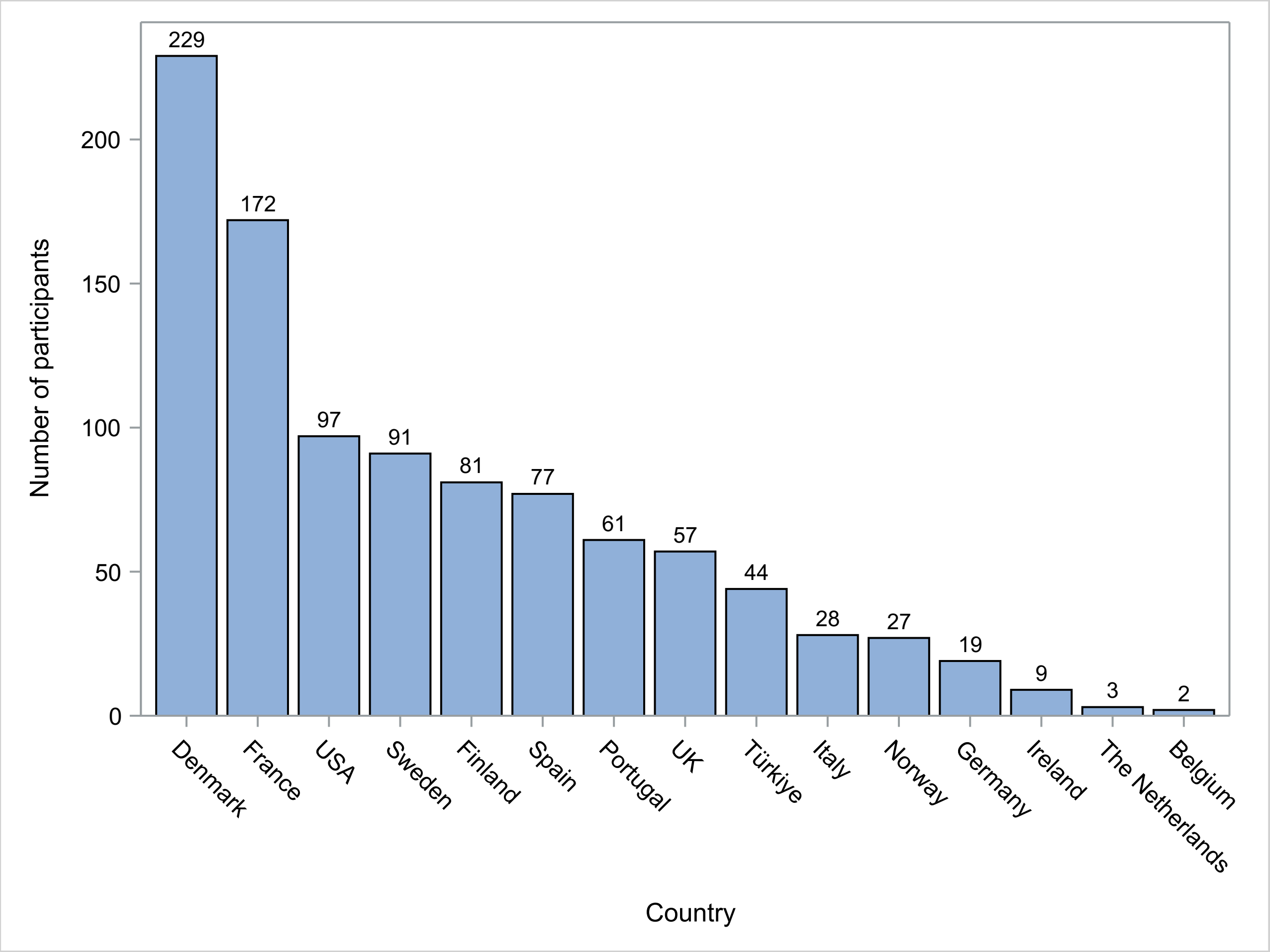

The Nine-I platelet survey included 977 participants from 15 different countries. 823 completed the English version of the survey, and 174 completed the French version. Most responses were from Denmark (23%) and France (18%), followed by the USA (10%), Sweden (9%), Finland (8%), and Spain (8%). Together, respondents from these 6 countries comprised 76% of the survey.

Table S1. Survey distribution, survey completion and response rate per country

Country	Distributed surveys (n)	Responses (n)	Response rate	Completed (n)	Completion rate	Distribution method
**Denmark**	442	290	66 %	229	79%	*Personal email by local investigators*
**France**	1200	211	18 %	172	82%	*By email to the GRRROH association (www.grrroh.fr)*
**USA**	334	121	36 %	97	80%	*Personal email by local investigators*
**Sweden**	253	133	53 %	91	68 %	*Email through the Swedish Society of Intensive Care Medicine (SIS)*
**Finland**	401	106	26 %	81	76 %	*Personal email by local investigator*
**Spain**	291	102	35 %	77	75 %	*Personal email by local investigators*
**Portugal**	Estimated 500	165	Appr. 33 %	63	38 %	*E-mails distributed through the Portuguese Society of Intensive Care Medicine (SPCI)*
**United Kingdom**	106	77	73%	57	74%	*Personal email by local investigators*
**Türkiye**	165	54	33 %	44	81 %	*Personal email by local investigators*
**Italy**	70	35	50 %	28	80 %	*Personal email by local investigator*
**Norway**	104	33	32 %	27	82%	*Personal email by local investigator*
**Germany**	Unknown	35	Unknown	19	54%	*Email distributed through mailing list by local investigator*
**Ireland**	28	15	54 %	9	60%	*Personal email by local investigator*
**Netherlands**	Unknown	4	Unknown	3	75%	*Personal email by local investigator*
**Belgium**	Included in the French distribution list	2	Unknown	2	100%	*By email to the GRRROH association (www.grrroh.fr)*
**Austria**	1	1	100 %	0	0 %	*Local investigator*
**Total**	Estimated approximately 4000	1384	Estimated 30-35%	999*	72%	

*Two respondents were excluded as they were not physicians, leaving a total of 997 completed surveys for analysis

Fig S2. Number of survey responses and completions per country

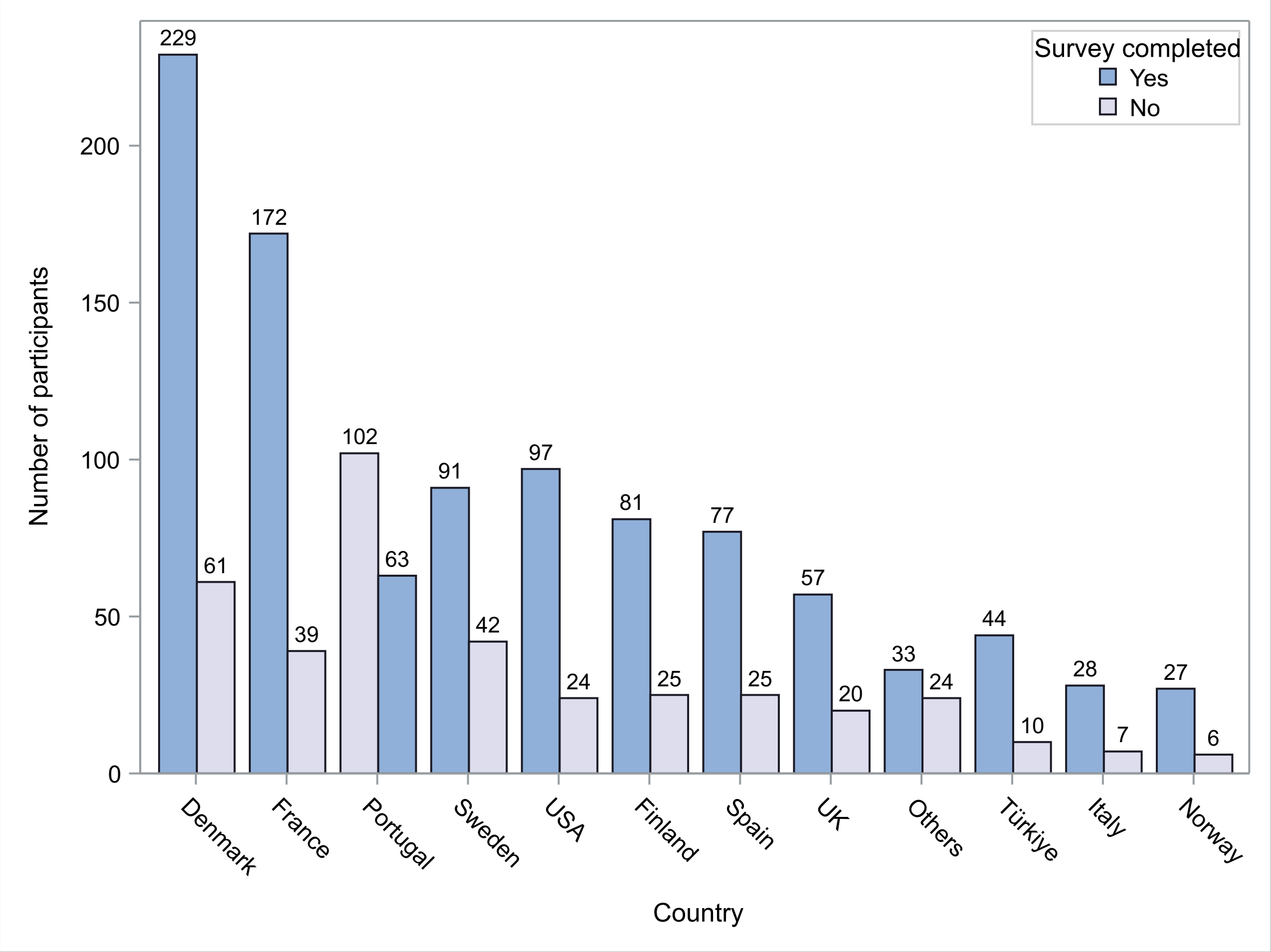

The survey was distributed to approximately 4450 ICU physicians; 2198 received the survey through direct email from one of the investigators. The remainder received the survey through distribution either through national societies (Portugal, Sweden) or through other work-related email lists (France, Germany). The completion rate varied but was around 70-80% in most countries, the noticeable exception being Portugal, where only 38% completed the survey.

Participant and hospital demographics per country

A: Table S2a. Participant demographics per country

	Denmark		Finland		France		Italy		Norway		Portugal	
	Median/ No	*IQR/ %*	*Median/ No.*	*IQR/ %*	*Median/ No.*	*IQR/ %*	*Median/ No.*	*IQR/ %*	*Median/ No.*	*IQR/ %*	*Median/ No.*	*IQR/ %*
**Age** (years)	45	40-52	45	39-53	37	32-45	39	31-42	47	42-54	40	33-49
**Gender:**												
- Female	90	39.3	36	44.4	64	37.2	15	53.6	4	14.8	24	39.3
- Male	136	59.4	43	53.1	107	62.2	12	42.9	21	77.8	37	60.7
- Non-binary and other	1	0.4	-	-	1	0.6	-	-	-	-	-	-
- Prefer not to answer	2	0.9	2	2.5	-	-	1	3.6	2	7.4	-	-
**ICU experience** (years)	9	4-15	11	4-20	7	4-15	7	3-10	10	5-18	6	3-14
**Primary speciality:**												
- Intensive Care Medicine [a)]	85	37.1	18	22.2	150	87.2	20	71.4	4	14.8	48	62.3
- Anaesthesiology	141	61.6	58	71.6	10	5.8	8	28.6	22	81.5	2	2.60
- Internal medicine	-		3	3.70	4	2.3	-	-	1	3.7	11	14.3
- Pulmonology	1	0.4	-	-	-	-	-	-	-	-	3	3.9
- Haematology	-	-	-	-	3	1.7	-	-	-	-	-	
- Gastroenterology and/or hepatology	-	-	-	-	-	-	-	-	-	-	7	9.1
- Surgery	1	0.4	1	1.2	-	-	-	-	-	-	4	5.2
- Other specialties[b)]	1	0.4	1	1.2	5	2.9	-	-	-	-	2	2.6

	Spain		Sweden		Türkiye		UK		USA		Others[c)]	
	Median/ No	*IQR/ %*	*Median/ No*	*IQR/ %*	*Median/ No*	*IQR/ %*	*Median/ No*	*IQR/ %*	*Median/ No*	*IQR/ %*	*Median/ No.*	*IQR/ %*
**Age** (years)	39	33-46	50	41-56	38	35-42	41	37-45	38	33-47	47	39-54
**Gender:**												
- Female	33	42.9	33	36.3	28	63.6	19	33.33	33	34.0	11	33.3
- Male	41	53.3	57	62.6	15	34.1	37	64.91	61	62.9	22	66.7
- Non-binary and other	-	-	-	-	-	-	-	-	1	1.0	-	-
- Prefer not to answer	3	3.9	1	1.1	1	2.3	1	1.75	2	2.1	-	-
**ICU experience (years):**	10	5-20	12	8-22	6	4-9	11	7-15	6	2-12	16	9-20
**Primary speciality:**												
- Intensive Care Medicine [a)]	48	62.3	63	69.2	24	54.6	45	79.0	61	62.9	22	66.7
- Anaesthesiology	2	2.6	28	30.8	4	9.1	11	19.3	9	9.3	5	15.2
- Internal medicine	11	14.3	-	-	11	25.0	-	-	13	13.4	4	12.1
- Pulmonology	3	3.9	-	-	3	6.8	1	1.8	10	10.3	-	-
- Haematology	-	-	-	-	-	-	-	-	2	2.1	2	6.1
- Gasteroenterology and/or hepatology	7	9.1	-	-	-	-	-	-	-	-	-	-
- Surgery	4	5.2	-	-	-	-	-	-	-	-	-	-
- Other specialties [b)]	2	2.6	-	-	2	4.6	-	-	2	2.1	-	-

[a)] 16 physicians responded that they were specialists in Intensive Care Medicine and Anaesthesiology (n=15) or Intensive Care Medicine and Pulmonology (n=1). Here, they are listed as Intensive Care Medicine specialists only.
[b)] Other specialties included neurology, oncology, pediatric critical care, emergency medicine, infectious diseases, cardiology, nephrology and burn specialists
c) Countries, here named 'Others', with less than 20 responses are analysed together: Germany, Ireland, The Netherlands and Belgium

Table S2b: Hospital demographics per country

	Denmark		Finland		France		Italy		Norway		Portugal	
	Median/ No	*IQR/ %*	*Median/ No*	*IQR/ %*	*Median/ No*	*IQR/ %*	*Median/ No*	*IQR/ %*	*Median/ No*	*IQR/ %*	*Median/ No*	*IQR/ %*
**Hospital type:**												
- University hospital	194	84.7	46	56.8	132	76.7	16	57.1	14	51.9	28	45.9
- University-affiliated hospital	34	14.9	33	40.7	23	13.4	11	39.3	7	25.9	22	36.1
- Regional hospital	1	0.4	2	2.5	17	9.9	1	3.6	6	22.2	11	18.0
**Funding:**												
- Public	228	99.6	76	93.8	158	91.9	24	85.7	26	96.3	54	88.5
- Private	-	-	-	-	6	3.5	1	3.6	0	-	5	8.2
- Mixed	1	0.4	5	6.2	8	4.7	3	10.7	1	3.7	2	3.3
**Presence of haematology department:**	147	64.2	65	80.3	143	83.1	21	75.0	19	70.4	44	72.1
**Presence of oncology department:**	171	74.7	64	79.0	155	90.1	24	85.7	14	51.9	46	75.4
**Number of ICU beds:**												
- < 10	54	23.6	35	43.2	17	9.9	11	39.3	10	37.0	17	27.9
- 10-19	82	35.8	13	16.1	52	30.2	15	53.6	15	55.6	20	32.8
- 20-29	82	35.8	23	28.4	68	39.5	1	3.6	2	7.4	13	21.3
- 30-39	4	1.8	2	2.5	28	16.3	1	3.6	0	0	5	8.2
> 40	7	3.1	8	9.9	7	4.1	0	0	0	0	6	9.8
**Type of patients:**												
- Medical	201	87.8	77	95.1	169	98.3	25	89.3	26	96.3	56	91.8
- Surgical (including trauma)	205	89.5	77	95.1	78	45.4	25	89.3	26	96.3	58	95.1
- Neurosurgical	66	28.8	49	60.5	16	9.3	5	17.86	12	44.4	32	52.5
- Cardiothoracic	70	30.6	46	56.8	31	18.0	6	21.43	3	11.1	14	23.0
- Haematological	102	44.5	64	79.0	150	87.2	15	53.57	19	70.4	48	78.7
- Oncological	123	53.7	61	75.3	164	95.4	15	53.57	18	66.7	49	80.3
- Burn patients	64	28.0	21	25.9	2	1.2	6	21.43	3	11.1	13	21.3
- SOT	69	30.1	14	17.3	114	66.3	7	25.00	11	40.7	19	31.2
- HSCT	55	24.0	16	19.8	109	63.4	9	32.14	11	40.7	12	19.7
- ECMO	178	77.7	16	19.8	82	47.7	10	35.71	11	40.7	12	19.7
**Presence of a local platelet transfusion protocol in hospital:**												
- Yes	147	64.2	10	12.4	32	18.6	11	39.3	11	40.7	23	37.7
- No	36	15.7	49	60.5	92	53.5	10	35.7	8	29.6	27	44.3
- I do not know	46	20.1	22	27.2	48	27.9	7	25.0	8	29.6	11	18.0
**Presence of a platelet transfusion protocol specific for ICU:**												
- Yes	26	11.4	13	16.1	19	11.1	3	10.7	2	7.41	14	23.0
- No	144	62.9	61	75.3	139	80.8	22	78.6	21	77.78	45	73.8
- I do not know	59	25.8	7	8.6	14	8.2	3	10.7	4	14.81	2	3.3

	Spain		Sweden		Türkiye		UK		USA		Others	
	Median/ No	*IQR/ %*	*Median/ No*	*IQR/ %*	*Median/ No*	*IQR/ %*	*Median/ No*	*IQR/ %*	*Median/ No*	*IQR/ %*	*Median/ No*	*IQR/ %*
**Hospital type:**												
- University hospital	74	96.1	52	57.1	31	70.5	43	75.4	83	85.6	23	69.7
- University-affiliated hospital	3	3.9	31	34.1	8	18.2	13	22.8	14	14.4	9	27.3
- Regional hospital			8	8.8	5	11.4	1	1.8			1	3.0

**Funding:**												
- Public	54	70.1	89	97.8	43	97.7	57	100	11	11.3	21	63.6
- Private	-	-	2	2.2	0	-	-	-	36	37.1	2	6.1
- Mixed	23	29.9	-	-	1	2.3	-	-	44	45.4	10	30.3
- Other funding sources	-	-	-	-	-	-	-	-	6	6.2	-	-
**Presence of haematology department in hospital:**	73	94.8	73	80.2	35	79.6	39	68.4	88	90.7	29	87.9
**Presence of oncology department in hospital:**	76	98.7	72	79.1	41	93.2	28	49.1	91	93.8	31	93.9
**Number of ICU beds:**												
- <10	1	1.3	64	70.3	3	6.8	5	8.8	8	8.3	4	12.1
- 10-19	38	49.4	23	25.3	19	43.2	19	33.3	8	8.3	8	24.2
- 20-29	16	20.8	3	3.3	12	27.3	20	35.1	38	39.2	14	42.4
- 30-39	4	5.2	1	1.1	4	9.1	2	3.5	10	10.3	2	6.1
- > 40	18	23.4	-	-	6	13.6	11	19.3	41	42.3	5	15.2
**Type of patients:**												
- Medical	72	93.5	88	96.7	44	100	56	98.3	94	96.9	33	100
- Surgical (including trauma)	50	64.9	86	94.5	31	70.5	56	98.3	68	70.1	24	72.7
- Neurosurgical	33	42.9	37	40.7	13	29.6	18	31.6	38	39.2	15	45.5
- Cardiothoracic	35	45.5	11	12.1	8	18.2	16	28.1	33	34.0	10	30.3
- Haematological	64	83.1	78	85.7	33	75.0	40	70.2	87	89.7	29	87.9
- Oncology	64	83.1	78	85.7	36	81.8	40	70.2	88	90.7	31	93.9
- Burn	5	6.5	15	16.5	9	20.5	25	43.9	5	5.2	10	30.3
- SOT	43	55.8	25	27.5	24	54.6	15	26.3	59	60.8	11	33.3
- HSCT	40	52.0	33	36.3	25	56.8	11	19.3	74	76.3	20	60.6
- ECMO	27	35.1	5	5.5	12	27.3	17	29.8	29	29.9	15	45.5
**Presence of a local platelet transfusion protocol in hospital:**												
- Yes	51	66.2	18	19.8	20	45.5	23	40.4	58	59.8	18	54.6
- No	12	15.6	43	47.3	20	45.5	19	33.3	15	15.5	11	33.3
- I do not know	14	18.2	30	33.0	4	9.1	15	26.3	24	24.7	4	12.1
**Presence of a platelet transfusion protocol specific for ICU:**												
- Yes	15	19.5	13	14.3	10	22.7	8	14.0	11	11.3	5	15.2
- No	48	62.3	68	74.7	31	70.5	43	75.4	49	50.5	27	81.8
- I do not know	14	18.2	10	11.0	3	6.8	6	10.5	37	38.1	1	3.0

Abbreviations: ICU: intensive Care Unit; ECMO: Extracorporeal membrane oxygenation; ECLS: Extracorporal life support; HSCT: Haematopoitic stem cell transplantation; SOT: Solid organ transplant patients

Use of guidelines per country

Table S3: Respondents reply to the question, 'Do you read the guidelines?'

	RESPONSES				
**Country**	***'Never'***	***'Rarely'***	***'Sometimes'***	***'Always'***	***'I do not know'***
**Denmark**	20 (8.7)	51 (22.3)	**124 (54.2)**	18 (7.9)	16 (7.0)
**Finland**	0	21 (25.9)	**51 (63.0)**	6 (7.4)	3 (3.7)
**France**	10 (5.8)	35 (20.4)	**86 (50.0)**	37 (21.5)	4 (2.3)
**Italy**	2 (7.1)	4 (14.3)	**14 (50.0)**	6 (21.4)	2 (7.1)
**Norway**	5 (18.5)	6 (22.2)	**13 (48.2)**	3 (11.1)	0
**Portugal**	8 (13.1)	4 (6.6)	**36 (59.0)**	11 (18.0)	2 (3.3)
**Spain**	4 (5.2)	**33 (42.9)**	29 (37.7)	8 (10.4)	3 (3.9)
**Sweden**	6 (6.6)	25 (27.5)	**49 (53.9)**	6 (6.6)	5 (5.5)
**Türkiye**	1 (2.3)	9 (20.5)	**23 (52.3)**	7 (15.9)	4 (9.1)
**UK**	4 (7.0)	16 (28.1)	**33 (57.9)**	1 (1.8)	3 (5.3)
**USA**	8 (8.3)	17 (17.5)	**54 (55.7)**	12 (12.4)	6 (6.2)
**Others***	0	5 (15.2)	**20 (60.6)**	8 (24.2)	0
**All**	68 (6.8)	226 (22.7)	**532 (53.4)**	123 (12.3)	48 (4.8)

Data are presented in numbers (percentages). The most common response in each country is marked with blue font.

* Countries with fewer respondents than 20 (Germany, Ireland, The Netherlands, Belgium) has been pooled and labeled 'Others'.

Figure S3: Physicians' responses to whether they read the transfusion guidelines per country

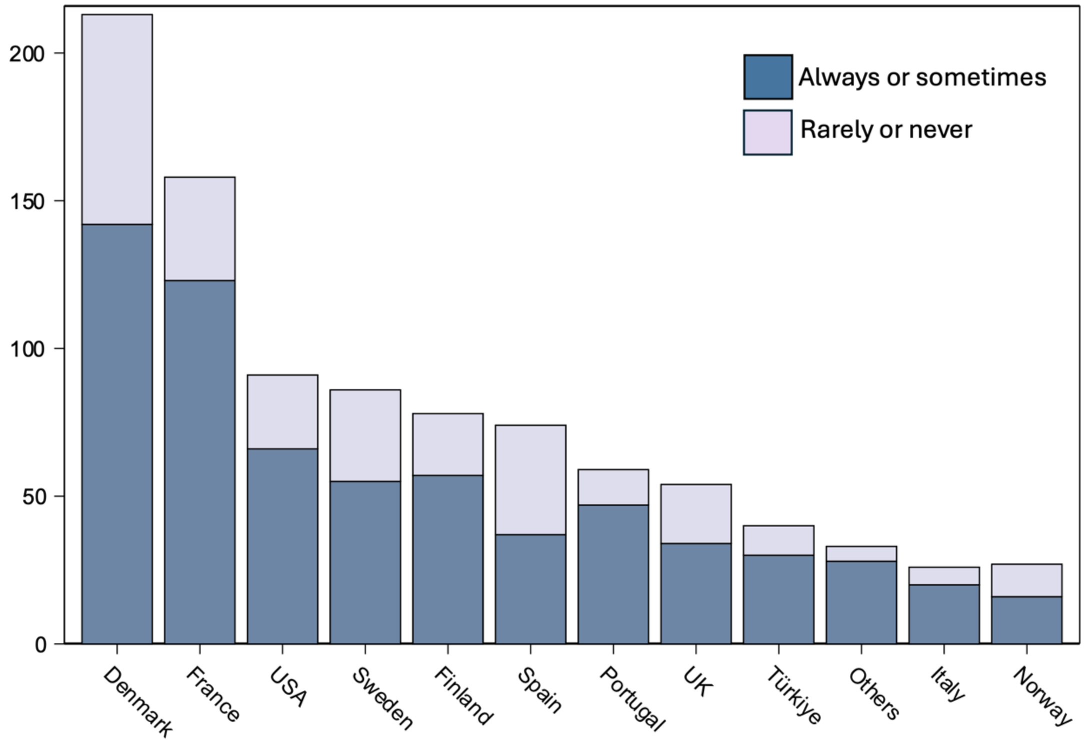

Shown in Fig S3 are the survey respondents' answers to the question: 'Do you read the published guidelines about platelet transfusions in the ICU?' Overall, 123 (12%) said they always read the guidelines, 226 (23%) said they sometimes read the guidelines, 226 (23%) rarely and 68 (7%) never.
48 respondents replied, 'I do not know'; these responses are not illustrated in the figure.
(UK = The United Kingdom, Others = Germany, Ireland, The Netherlands, Belgium and Austria)

Prophylactic platelet transfusion thresholds in medical ICU patients per country

Table S4. Prophylactic platelet transfusion threshold preferences in thrombocytopenic medical ICU patients without bleeding in the different countries

Country	10 x 10^9/L	20 x 10^9/L	30 x 10^9/L	40 x 10^9/L	50 x 10^9/L	Do not use	Other thresholds[a)]
**Denmark**	**120 (52.4)**	43 (18.8)	8 (3.5)	3 (1.3)	3 (1.3)	40 (17.5)	12 (5.2)
**Finland**	**38 (46.9)**	14 (17.3)	2 (2.5)	1(1.2)	0	22 (27.2)	4 (4.9)
**France**	67 (39.0)	**78 (45.4)**	4 (2.3)	1 (0.6)	1 (0.6)	16 (9.3)	5 (2.9)
**Italy**	**11 (39.3)**	4 (14.3)	2 (7.1)	0	2 (7.1)	8 (28.6)	1 (3.6)
**Norway**	**11 (40.7)**	8 (29.6)	0	0	0	7 (25.9)	1 (3.7)
**Portugal**	**32 (52.5)**	7 (11.5)	0	0	1 (1.6)	18 (29.5)	3 (4.9)
**Spain**	**31 (40.3)**	25(32.5)	6 (7.8)	1 (1.3)	4 (5.2)	9 (11.7)	1 (1.3)
**Sweden**	**42 (46.2)**	17 (18.7)	7 (7.7)	0	0	22 (24.2)	3 (3.3)
**Türkiye**	**24 (54.6)**	12 (27.3)	0	0	3 (6.8)	4 (9.1)	1 (2.3)
**UK**	19 (33.3)	**26 (45.6)**	1 (1.8)	1 (1.8)	0	7 (12.3)	3 (5.3)
**USA**	**76 (78.4)**	6 (6.2)	1 (1.0)	0	0	13 (13.4)	1 (1.0)
**Others**	**19 (57.6)**	8 (24.2)	0	0	1 (3.0)	5 (15.2)	0
**All**	**490 (49.2)**	248 (24.9)	31 (3.1)	7 (0.7)	15 (1.5)	171 (17.2)	35 (3.5)

Data are presented in numbers (percentages). The threshold with the highest number of responses in each country is highlighted.
[a)] The most common replies in the category 'other thresholds' were variations of: 1) It depends on the clinical situation (n=15), 2) I would consult a haematologist or another specialist (n=6), 3) I use other thresholds (e.g. 2x10^9/L, 4 x 10^9/L, 5x10^9/L, 15x10^9/L, 80x10^9/L) (n=7) The most common response in each country is marked with blue font.

[b)] Countries with fewer respondents than 20 (Germany, Ireland, The Netherlands, Belgium) has been pooled and labeled 'Others'**.**

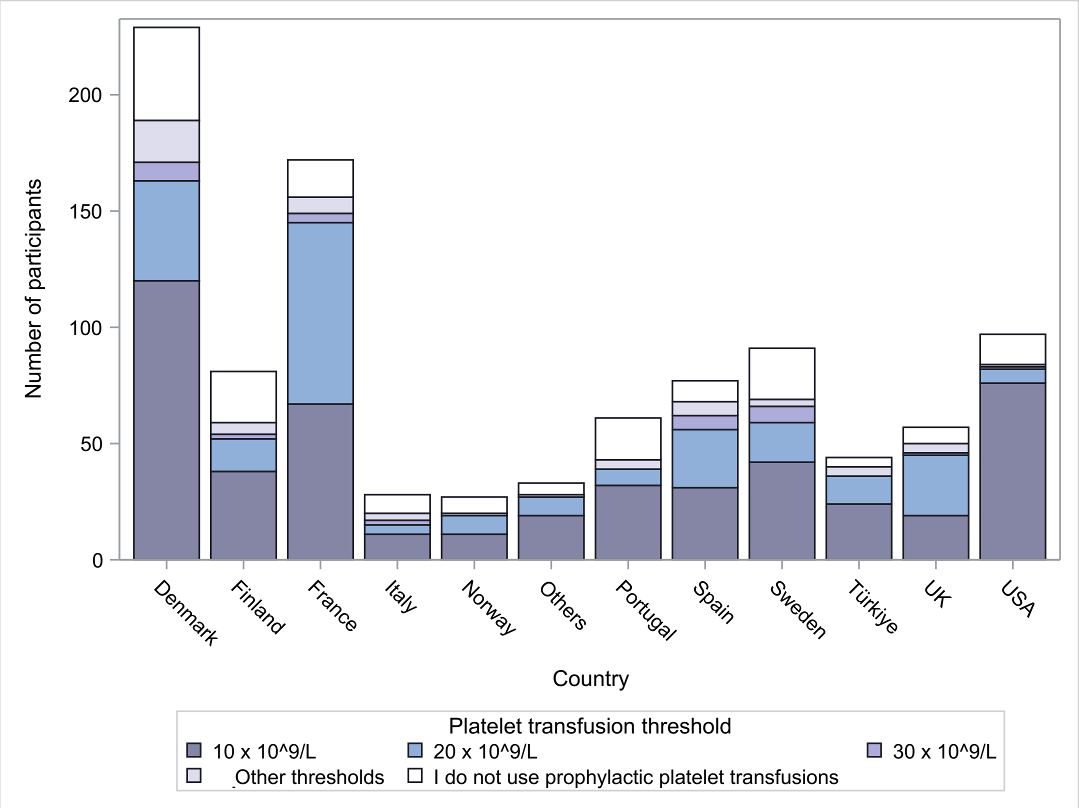

Fig S4: Preferred prophylactic platelet transfusion threshold in thrombocytopenic non-bleeding medical ICU patients, here shown per country. The corresponding numbers and percentages can be found in Table S4. Countries with fewer respondents than 20 (Germany, Ireland, The Netherlands, Belgium) have been pooled and labelled 'Others'. Categories with less than 25 responses are pooled into the category 'Other thresholds'.

Selected quotes illustrating the most common responses to why physicians change transfusion strategy in patients with bone-marrow failure

Table S5. Prophylactic platelet transfusions in patients with bone-marrow failure

**A: Reasons for a more liberal transfusion strategy**
**Higher perceived risk of severe thrombocytopenia**
Knowing that a "hypoproliferative" thrombocytopenia patient might be at risk of severe thrombocytopenia < 10 mia/l during the day if you only measure TBC count once pr.day
I have the feeling the thrombocytopenia is more severe and spontaneous in these patients
Production would probably be more impaired, so I would prophylactically transfuse the patient before achieving such a low limit
**Higher perceived bleeding risk**
Suspicion of increased bleeding risk in primary thrombocytopaenic patients, as opposed to reactive thrombocytopaenia (such as in sepsis). In practice would be guided by haematology as overall less experience in this patient group
There can also be malfunctioning thrombocytes and spontaneous bleeding is more probable
Fear of intracerebral hemorrhage
**Dysfunctional platelets**
Patients own platelets are considered less effective
Atteinte qualitative de la production plaquettaire prévisible, en plus de l'atteinte quantitative (English: Predictable qualitative impairment of platelet production, in addition to quantitative impairment)
Pas de réserve plaquettaire et plaquettes moins efficientes selon l'étiologie. (English: No platelet reserve and less efficient platelets depending on etiology)
**B: Reasons for a more restrictive transfusion strategy**
**Platelet transfusions will not work**
Prophylactic platelet transfusions in these patients are less beneficial, yield less, and are therefore somewhat purposeless.
C'est logique. Rendement transfusionnel à priori faible donc sans intérêt en contexte d'hémorragie, de sepsis ou de risque hémorragique. (English: This is logical. The transfusion yield is a priori low and, therefore, of no interest in the context of haemorrhage, sepsis or risk of haemorrhage.)
Le rendement sera inefficace au long terme et la thrombocytopénie sera prolongée dans le temps (sans facteur rapidement réversible). (English: Yield will be ineffective over the long term and thrombocytopenia will be prolonged (without rapidly reversible factors))
**Fear of allo-immunization**
The need for transfusions leads to transfusion rejection and greater difficulty with transfusions once needed
Because increased need for platelet transfusions will increase the risk of irregular antibodies
**Perceived tolerance to severe thrombocytopenia**
Physiologically adapted to living with severe thrombocytopenia and because the half-life of transfused platelets is of few hours
Because the patient is used to having low platelets. Discussable how good it will work
Usually, these patients tolerate lower levels of thrombocytes

Fig S5A – D: Importance of blood test on the decision to transfuse prophylactic platelets

The respondents were asked to consider the importance of various commonly used coagulation blood tests in decision-making when prescribing prophylactic platelet transfusions in thrombocytopenic patients without bleeding.

The importance of the different blood tests was ranked from 1 to 5; 1=no influence on the decision, 2=some influence, 3=moderate influence, 4=substantial influence, 5=complete influence on the decision, and 6=I do not know/no opinion

Fig S5A: Fibrinogen

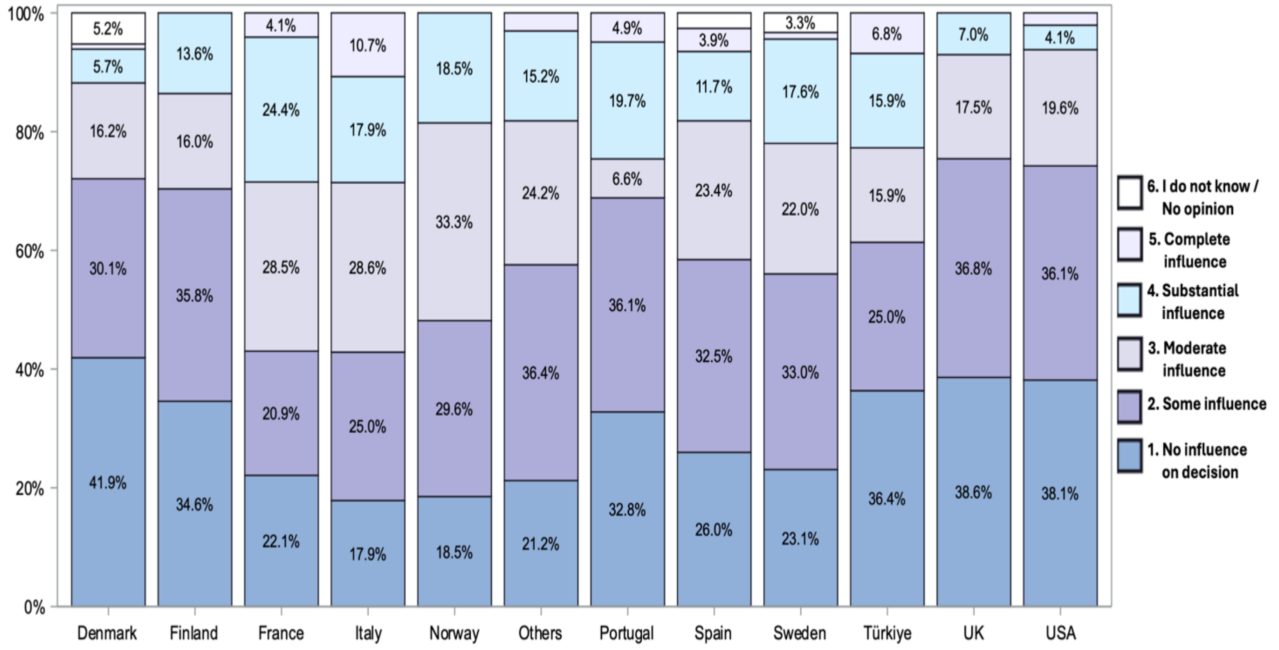

Fig S5B: Prothrombin time (PT) / International normalised ratio (INR)

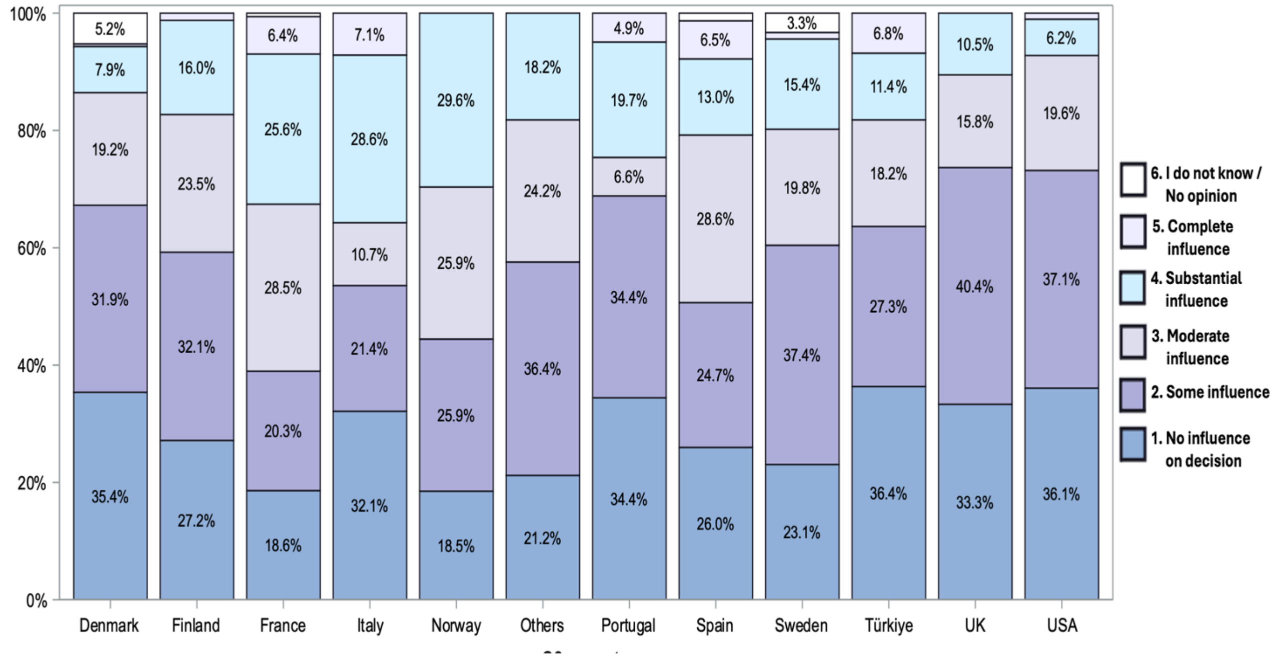

Fig S5B: Activated partial thromboplastin time (aPTT)

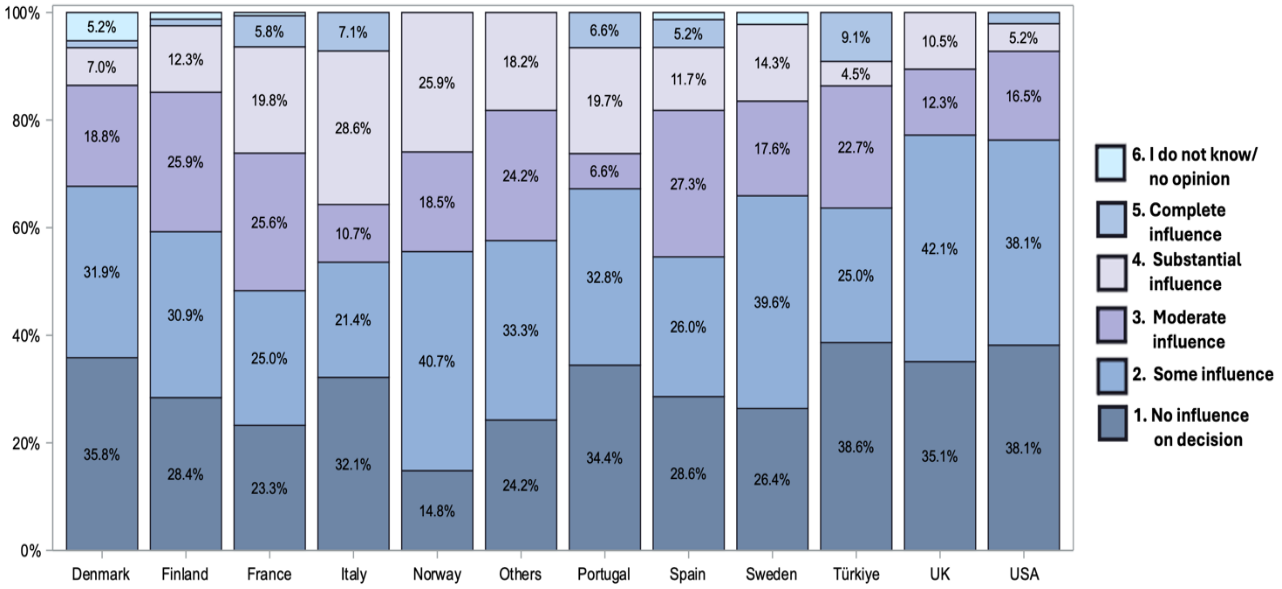

FIG S5C: Thromboelastography (TEG)/Rotational thromboelastometry (ROTEM)

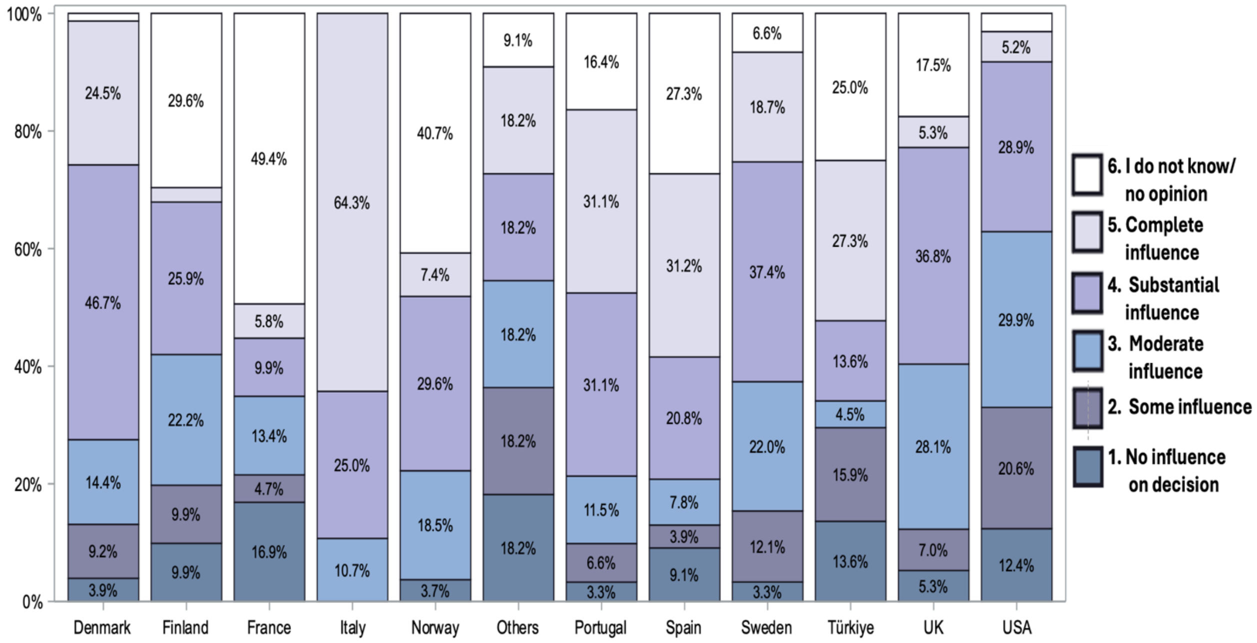

FIG S5D: Multiple electrode aggregometry (e.g. Multiplate Analyzer)

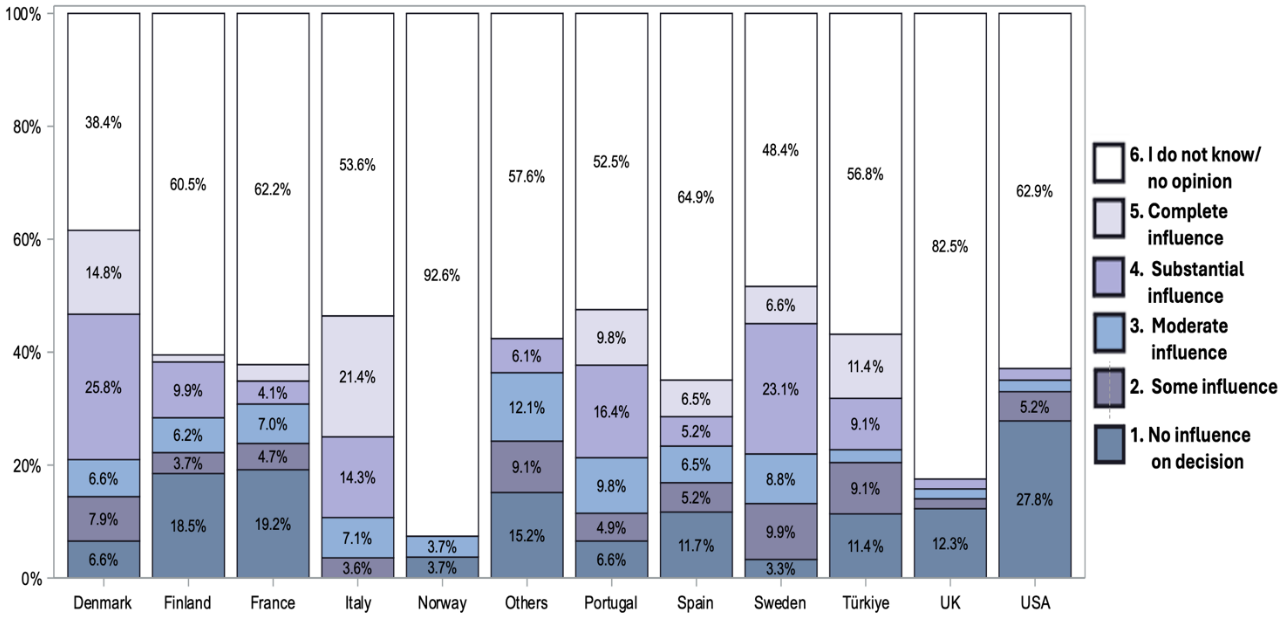

Preferred prophylactic platelet transfusion thresholds in surgical patients per country

Table S6. Prophylactic platelet transfusion threshold preferences for surgical patients without bleeding

Country	10 x 10^9/L	20 x 10^9/L	30 x 10^9/L	40 x 10^9/L	50 x 10^9/L	80 x 10^9/L	Do not use	Other thresholds[a)]
**Denmark**	**61 (26.6)**	54 (23.6)	18 (7.9)	7 (3.1)	48 (21.0)	6 (2.6)	22 (9.6)	13(5.7)
**Finland**	16 (19.8)	12 (14.8)	5 (6.2)	5(6.2)	**25 (30.9)**	2 (2.5)	8 (9.9)	8 (9.9)
**France**	17 (9.9)	37 (21.5)	18 (10.5)	4 (2.3)	**83 (48.3)**	3 (1.7)	5 (2.9)	5 (2.9)
**Italy**	4 (14.3)	3 (10.7)	5 (17.9)	1 (3.6)	**11 (39.3)**	0	3 (10.7)	1 (3.6)
**Norway**	2 (7.4)	**10 (37.0)**	1 (3.7)	1 (3.7)	9 (33.3)	0	4 (14.8)	0
**Portugal**	**25 (41.0)**	4 (6.6)	5 (8.2)	1 (1.6)	13 (21.3)	0	11 (18.0)	2 (3.3)
**Spain**	10 (13.0)	15(19.5)	7(9.1)	1 (1.3)	**37 (48.1)**	3 (3.9)	4 (5.2)	0
**Sweden**	14 (15.4)	**26 (28.6)**	18(19.8)	1 (1.1)	15 (16.5)	2 (2.2)	11 (12.1)	4 (4.4)
**Türkiye**	14 (31.8)	8 (18.2)	4 (9.1)	0	**16 (36.4)**	0	0	2 (4.6)
**UK**	14 (24.6)	**25 (43.9)**	3 (5.3)	1 (1.8)	10(17.5)	1 (1.8)	2 (3.5)	1 (1.8)
**USA**	**35 (36.1)**	21(21.7)	4 (4.1)	1(1.0)	24(24.8)	0	8 (8.3)	4(4.1)
**Others[b)]**	8 (24.2)	6 (18.2)	4 (12.1)	0	**9 (27.3)**	0	4 (12.1)	2 (6.1)
**ALL**	220 (22.1)	221 (22.2)	92 (9.2)	23 (2.3)	**300 (30.1)**	17 (1.7)	82 (8.2)	42 (4.2)

Data are presented in numbers (percentages). The threshold with the highest number of responses in each country is highlighted.
[a)] The most common replies in the category 'other' were variations of: 1) It depends on the clinical situation (n=26); here, the most common replies were the type of surgery (n=10) and time from surgery (n=7) 2) Transfusion is guided by TEG/ROTEM (n=6), 3) I use other thresholds (5x10^9/L, 30-50 x 10^9/L, 100x10^9/L) (n=4) The most common response in each country is marked with blue font.
[b)] Countries with fewer responses than 20 were pooled in the statistical analyses and labelled 'Others'.

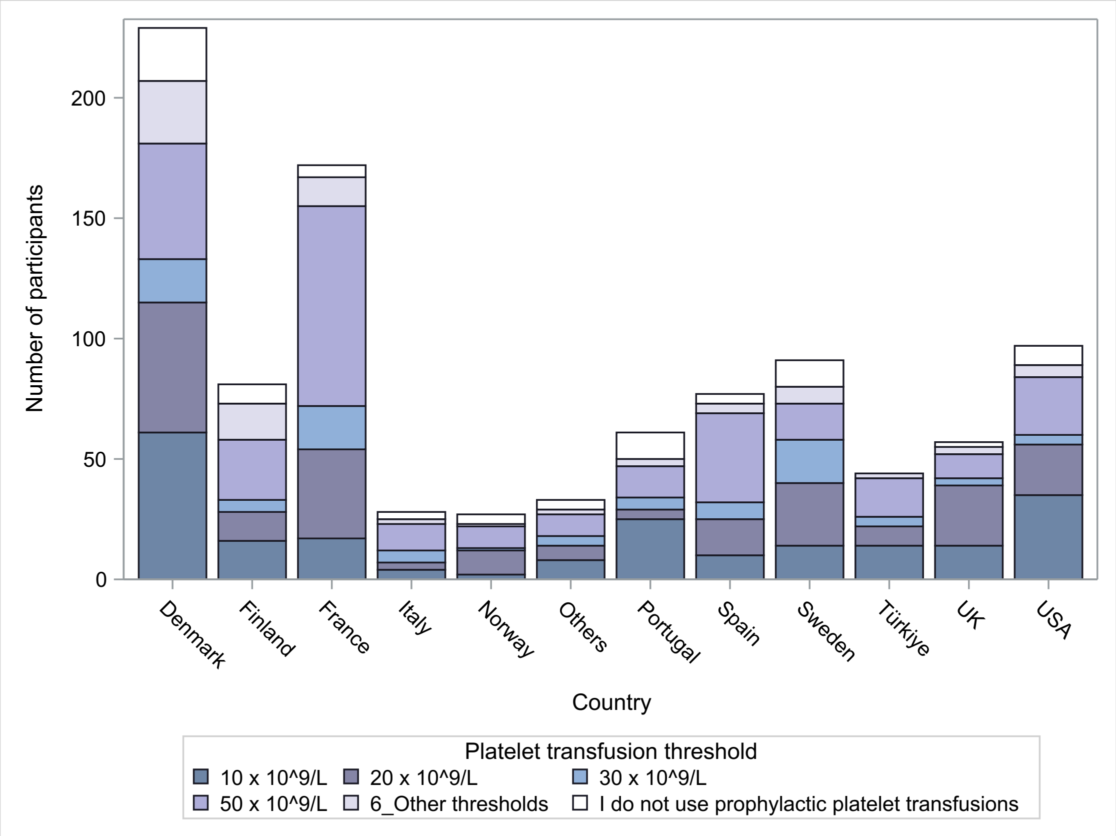

Figure S6. Preferred prophylactic platelet transfusion threshold in thrombocytopenic surgical ICU patients without bleeding, here shown per country. The corresponding numbers and percentages can be found in Table S7. Countries with fewer respondents than 20 (Germany, Ireland, The Netherlands, Belgium) have been pooled and labelled 'Others'.
Categories with less than 25 responses are pooled into the category 'Other thresholds'.

Preferred prophylactic platelet thresholds prior to different invasive procedures

A: PRIOR TO PLACING A CENTRAL VENOUS CATHETER (CVC)

Table S7: Preferred platelet prophylactic platelet threshold prior to placing a CVC

Threshold (cells/litre)	Number of respondents (%)
10 x 10^9	114 (11.4)
20 x 10^9	258 (25.9)
30 x 10^9	103 (10.3)
40 x 10^9	34 (3.4)
50x 10^9	305 (30.6)
80 x 10^9	16 (1.60)
100 x 10^9	1 (0.1)
Other preferred thresholds	42 (4.2)
Do not use prophylactic platelet transfusions	124 (12.4)

Fig S7A. Preferred platelet prophylactic platelet threshold prior to placing a CVC

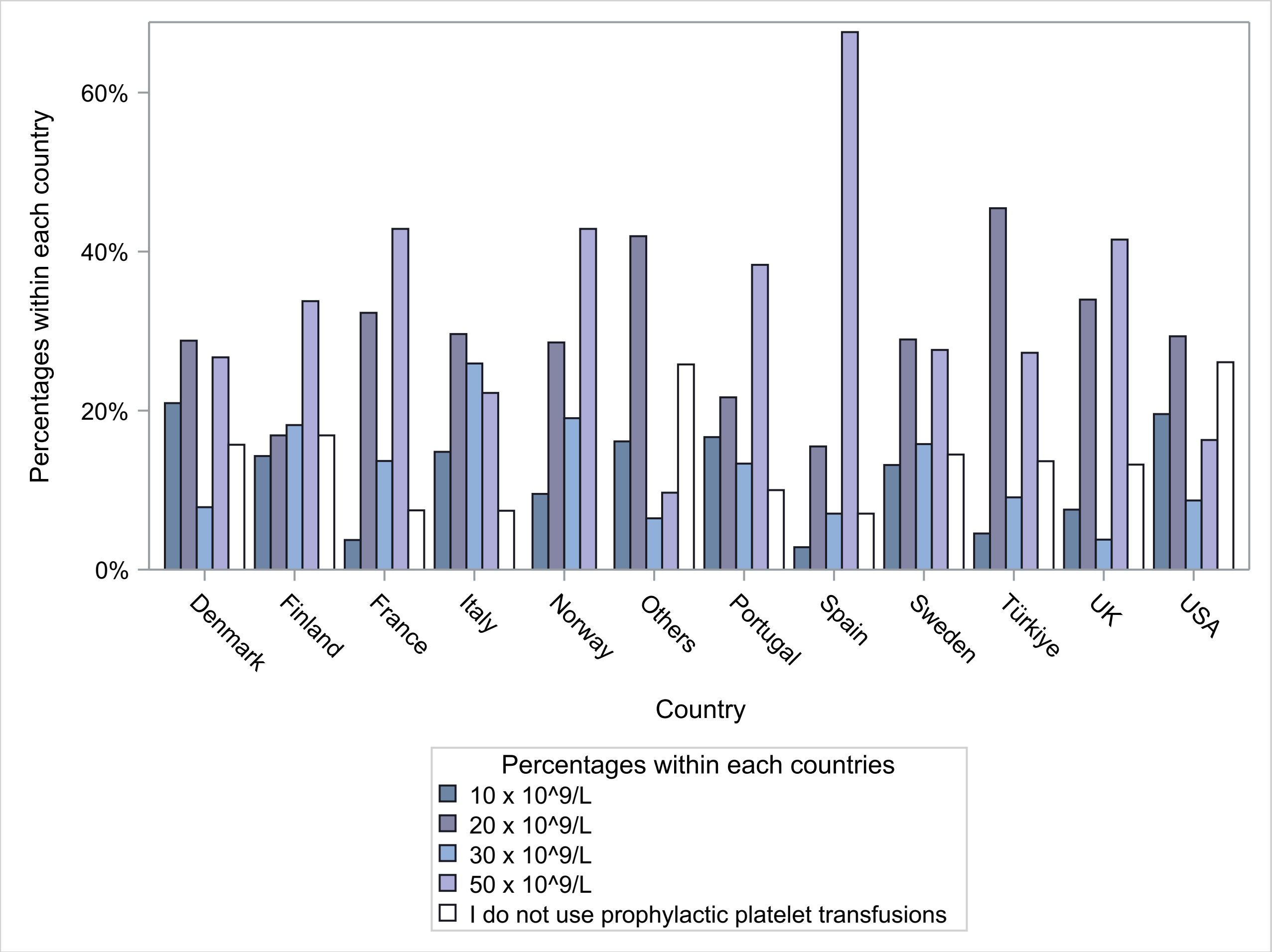

Thresholds preferred by less than 5% of respondents are not shown in the figure. All responses can be viewed in Table S7.

B: PRIOR TO A BRONCHOSCOPY

Table S8: Preferred platelet prophylactic platelet threshold prior to a bronchoscopy

Threshold (cells/litre)	Number of respondents (%)
10 x 10^9	64 (6.4)
20 x 10^9	191 (19.2)
30 x 10^9	95 (9.5)
40 x 10^9	41 (4.1)
**50 x 10^9**	**346 (34.7)**
80 x 10^9	33 (3.3)
Other preferred thresholds	44 (4.4)
Do not use prophylactic platelet transfusions	149 (14.9)
I do not know	5 (0.50)
We do not perform bronchoscopies in my ICU	29 (2.9)

Figure S8: Preferred platelet prophylactic platelet threshold prior to a bronchoscopy

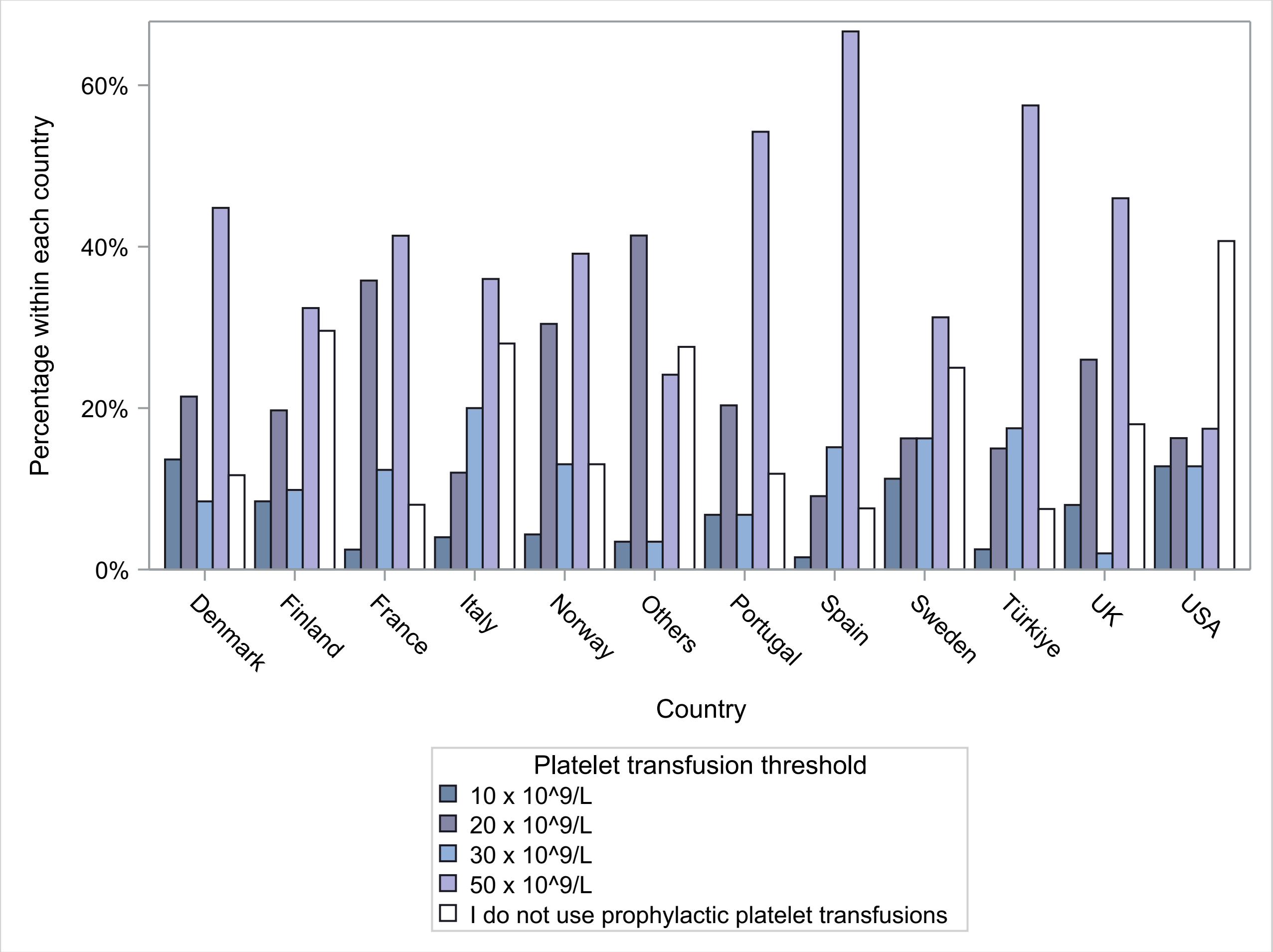

Thresholds preferred by less than 5% of respondents are not shown in the figure. All responses can be viewed in Table S8.

C: PRIOR TO A PERCUTANEOUS DILATATION TRACHEOSTOMY (PDT)

Table S9: Preferred platelet prophylactic platelet threshold prior to a PDT

**Platelet count** (cells/litre)	**Number of respondents (%)**
10 x 10^9	12 (1.2)
20 x 10^9	46 (4.6)
30 x 10^9	42 (4.21)
40 x 10^9	32 (3.21)
**50 x 10^9**	**540 (54.2)**
80 x 10^9	115 (11.5)
100 x 10^9	109 (10.9)
150 x 10^9	3 (0.30)
Other preferred thresholds	45 (4.5)
Do not use prophylactic platelet transfusions	12 (1.2)
I do not know	3 (0.3)
We do not perform PDTs in my ICU	38 (3.8)

Figure S9. Preferred platelet prophylactic platelet threshold prior to a PDT

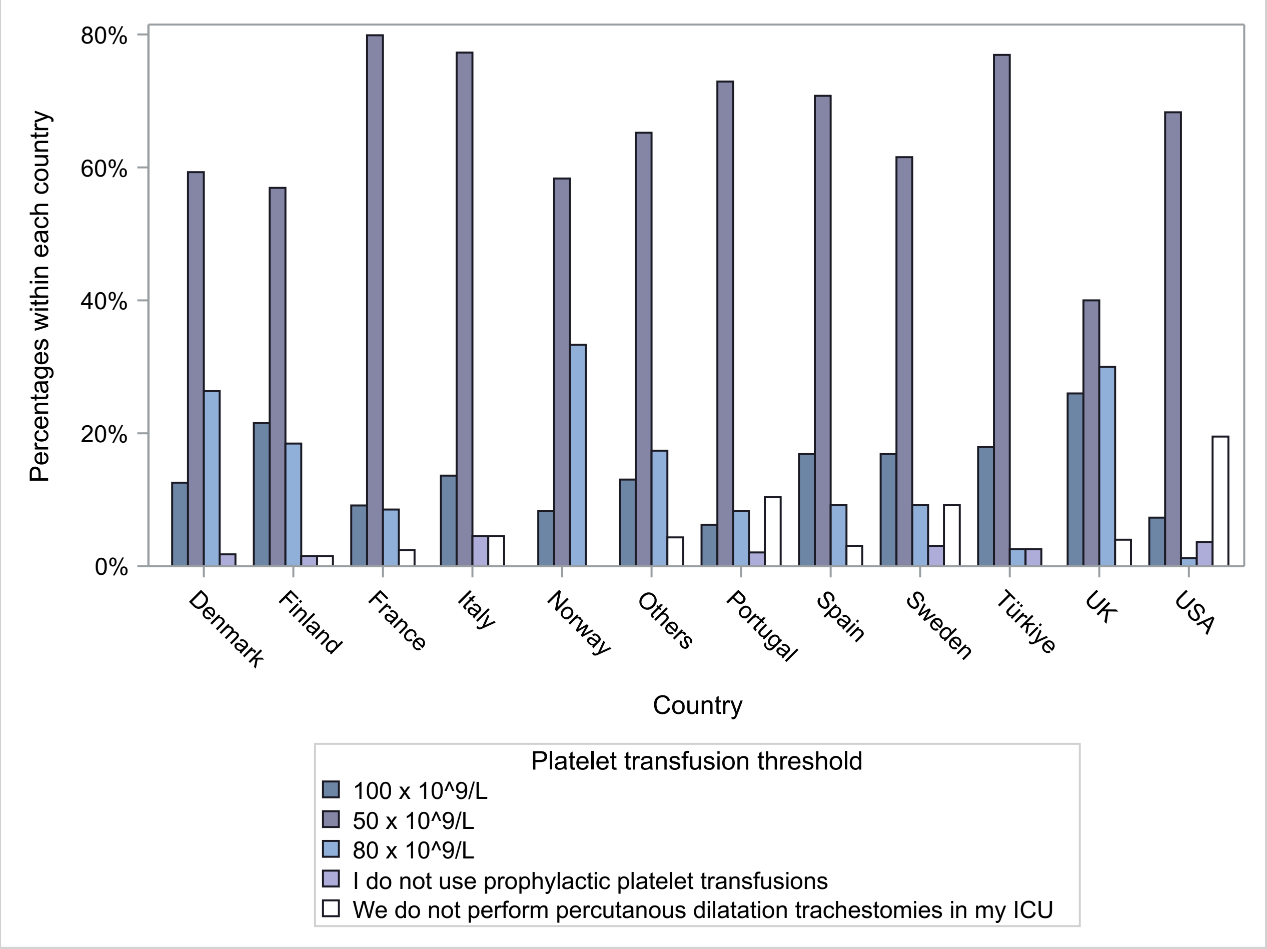

Thresholds preferred by less than 5% of respondents are not shown in the figure. All responses can be viewed in Table S9.

D: PRIOR TO A LUMBAR PUNCTURE (LP)

Table S10: Preferred platelet prophylactic platelet threshold prior to a LP

Threshold (cells/litre)	Number of respondents (%)
10 x 10^9	6 (0.6)
20 x 10^9	25 (2.5)
30 x 10^9	23 (2.3)
40 x 10^9	23 (2.3)
50 x 10^9	398 (39.9)
80 x 10^9	251 (25.2)
100 x 10^9	192 (19.3)
150 x 10^9	8 (0.8)
Other preferred thresholds	61 (6.1)
Do not use prophylactic platelet transfusions	10 (1.0)

Figure S10. Preferred platelet prophylactic platelet threshold prior to a LP

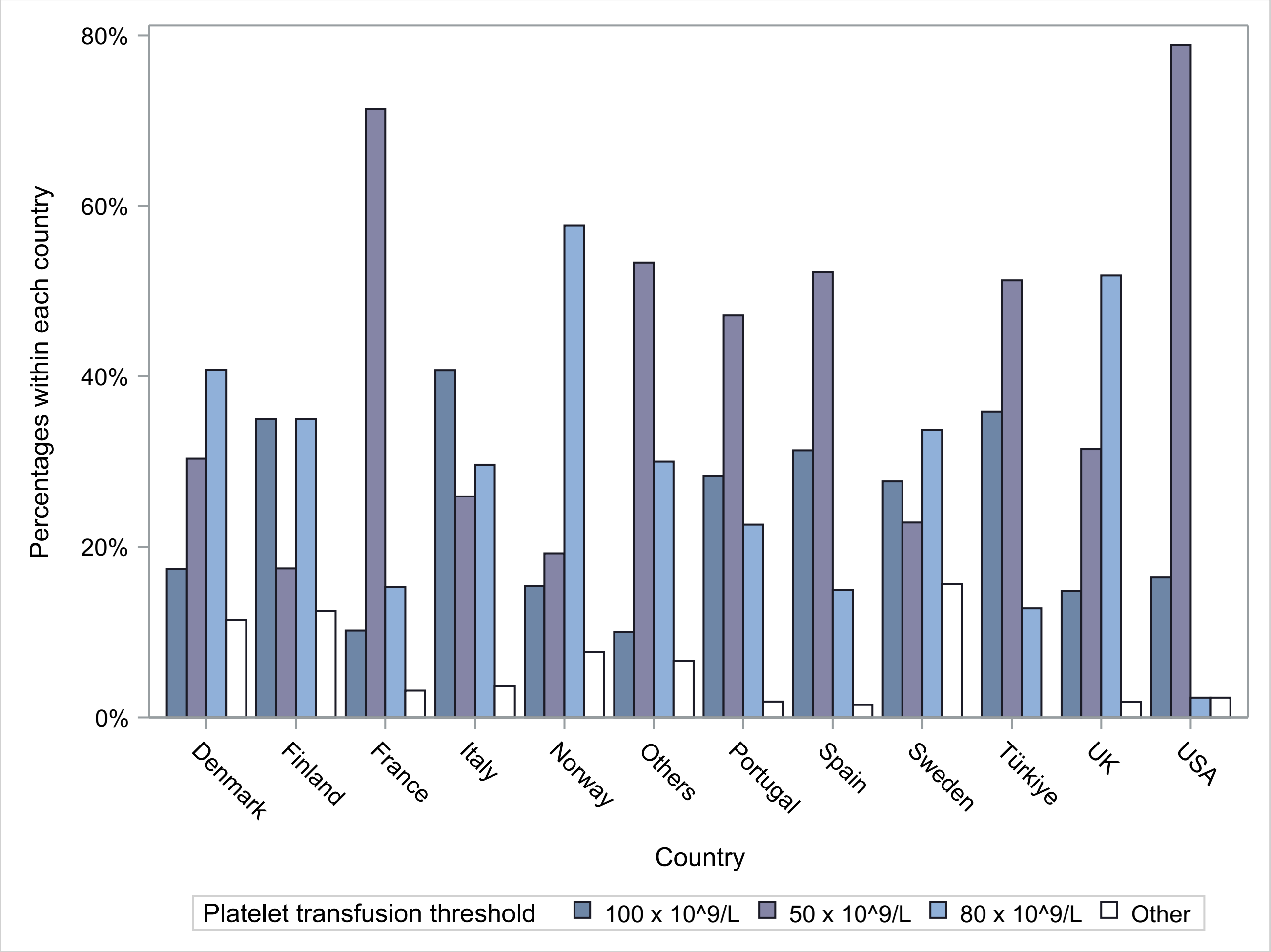

Thresholds preferred by less than 5% of respondents are not shown in the figure. All responses can be viewed in Table SX.

Platelet transfusion thresholds in patients with *minor bleeding* per country

Table S11. Platelet transfusion threshold preferences in ICU patients with minor bleeding (WHO I-II)

Country	10 x 10^9/L	20 x 10^9/L	30 x 10^9/L	40 x 10^9/L	50 x 10^9/L	80 x 10^9/L	100 x 10^9/L	Do not use	Other thresholds or strategies[a)]
**Denmark**	21 (9.2)	**72 (31.4)**	23 (10.0)	12 (5.2)	64 (28.0)	12 (5.6)	1(0.4)	14 (6.1)	10(4.4)
**Finland**	3(3.7)	8 (9.9)	14 (17.3)	6 (7.4)	**35 (43.2)**	7 (8.6)	2 (2.5)	4 (4.9)	2 (2.5)
**France**	7 (4.1)	46(26.7)	23(13.4)	3 (1.7)	**87 (50.6)**	1 (0.6)	3 (1.7)	1(0.6)	1 (0.6)
**Italy**	3 (10.7)	7(25.0)	5(17.9)	0	**8 (28.6)**	0	3 (10.7)	1(3.6)	1 (3.6)
**Norway**	4 (14.8)	**10 (37.0)**	4 (14.8)	0	8 (29.6)	1 (3.7)	0	0	0
**Portugal**	11(18.0)	12(19.7)	9(14.8)	1 (1.6)	**18 (29.5)**	0	0	10 (16.4)	0
**Spain**	4(5.2)	19(24.7)	11(14.3)	2 (2.6)	**32 (41.6)**	5 (6.5)	2(2.6)	2(2.6)	0
**Sweden**	9(9.9)	23(25.3)	17(18.7)	2 (2.2)	**32(35.2)**	5 (5.5)	3(3.3)	0	0
**Türkiye**	10(22.7)	**14(31.8)**	7 (15.9)	0	12 (27.3)	0	1(2.3)	0	0
**UK**	5 (8.8)	14(24.6)	5(8.8)	1 (1.8)	**27(47.4)**	3(5.3)	1(1.8)	0	1 (1.8)
**USA**	20(20.6)	**38(39.2)**	10(10.3)	1(1.0)	19(19.6)	1(1.0)	1(1.0)	7(7.1)	0
**Others[b)]**	2(6.1)	13(39.4)	5(15.2)	2(6.1)	**8 (24.2)**	1(3.0)	0	2 (6.1)	0
**All**	99 (9.9)	276 (27.7)	133 (13.3)	30 (3.0)	**350 (35.1)**	36 (3.6)	17 (1.7)	41 (4.1)	15 (1.5)

Data are presented in numbers (percentages). The threshold with the highest number of responses in each country is highlighted.
a) The most common replies in the category 'other thresholds' were variations of: 1) It depends on the clinical situation (n=7); Transfusion is guided by TEG/ROTEM and/or MEA (n=5). The most common response in each country is marked with blue font.

b) Countries with fewer responses than 20 were pooled in the statistical analyses and labelled 'Others'.

Figure S11. Preferred prophylactic platelet transfusion threshold preferences in ICU patients with minor bleeding (WHO I-II)

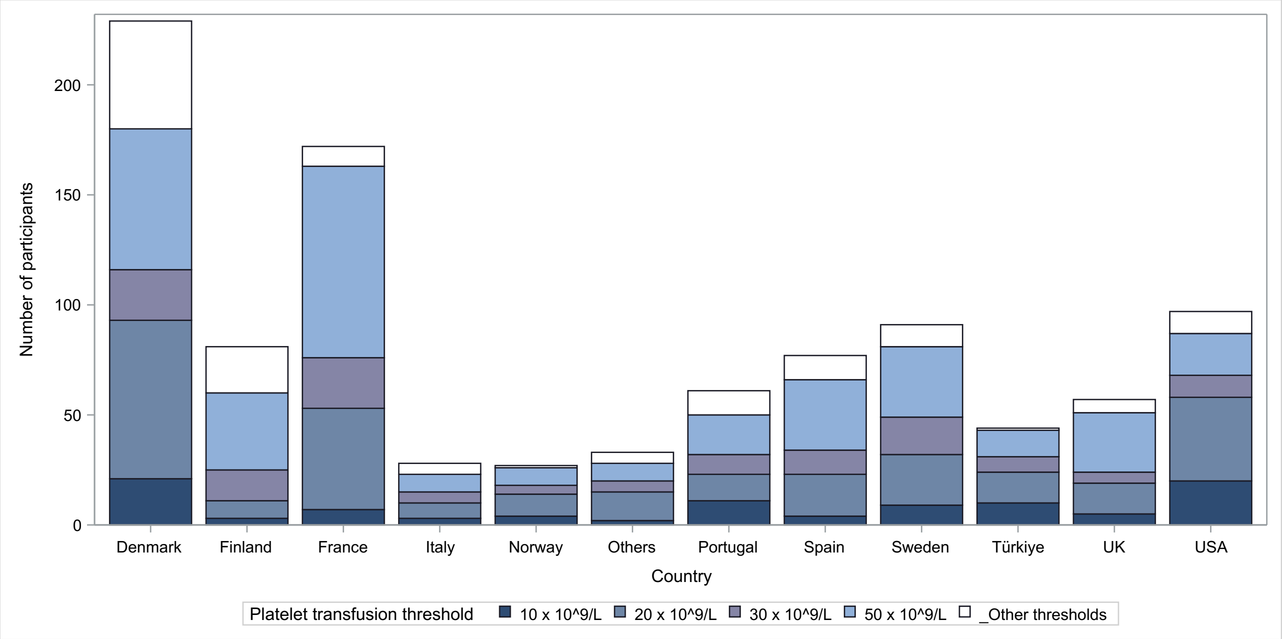

Thresholds preferred by less than 5% of all respondents are pooled as 'Other thresholds'. All responses can be viewed in Table S11.

Preferred platelet transfusion thresholds in patients with *major bleeding* per country

Table S12. Platelet transfusion threshold preferences in ICU patients with major bleeding (WHO III-IV)

Country	10 x10^9/L	20 x10^9/L	30 x10^{9}L	40 x10^9/L	50 x10^9/L	80 x10^9/L	100 x10^9/L	150 x 09/L	Do not use	Other thresholds or strategies[a)]
**Denmark**	6(2.6)	2 (0.9)	8 (3.5)	18(7.9)	**62 (27.1)**	56(24.5)	37(16.2)	7(3.1)	3 (1.3)	30(13.1)
**Finland**	0	0	0	0	17(21.0)	15(18.5)	**43(53.1)**	2(2.5)	0	4 (4.9)
**France**	0	4(2.3)	0	2(1.2)	**105(61.1)**	18 (10.5)	37(21.5)	0	0	6(3.5)
**Italy**	1(3.6)	0	1(3.6)	1(3.6)	**12 (42.9)**	8(28.6)	4 (14.3)	1(3.6)	0	0
**Norway**	1 (3.7)	0	0	0	**10 (37.0)**	4 (14.8)	8 (29.6)	1 3.7)	0	3 (11.1)
**Portugal**	6(9.8)	1 (1.6)	4(6.6)	1 (1.6)	**26 (42.6)**	8(13.1)	14(23.0)	0	0	1(1.6)
**Spain**	1(1.3)	4(5.2)	1(1.3)	4(5.2)	**32 (41.6)**	12(15.6)	20(26.0)	3(3.9)	0	0
**Sweden**	1(1.1)	1(1.1)	4(4.4)	0	20(22.0)	15 16.5)	**44(48.4)**	4(4.4)	0	2(2.2)
**Türkiye**	2(4.6)	2(4.6)	1(2.3)	0	**23(52.3)**	2(4.6)	13(29.6)	0	0	1(2.3)
**UK**	1(1.8)	4(7.0)	3(5.3)	0	**23(40.4)**	7(12.3)	18(31.6)	0	0	1 (1.8)
**USA**	2(2.1)	10(10.3)	3(3.1)	0	**50(51.6)**	0	25(25.8)	0	0	7(7.2)
**Others**	2(6.1)	1(3.0)	3(9.1)	2(6.1)	**15 (45.5)**	4(12.1)	5(15.2)	0	0	1 (3.0)
**Total**	23(2.3)	29 (2.9)	28(2.8)	28(2.8)	**395(39.6)**	149(14.9)	268(26.9)	18(1.8)	3(0.3)	56 (5.5)

Data are presented in numbers (percentages). The threshold with the highest number of responses in each country is highlighted.
a) The most common replies in the category 'other' were variations of 1) Use of balanced transfusion or massive transfusion protocols (n=16), 2) Depends on the clinical context (n=13); here, N=6 specifically mentioned they would increase threshold if CNS bleeding, 3) Transfusion would be guided by TEG/ROTEM and/or MEA(N=13). The most common response in each country is marked with blue font.
b) Countries with fewer responses than 20 were pooled in the statistical analyses and labelled 'Others'.

Fig S12. Platelet transfusion threshold preferences in ICU patients with major bleeding (WHO III-IV)

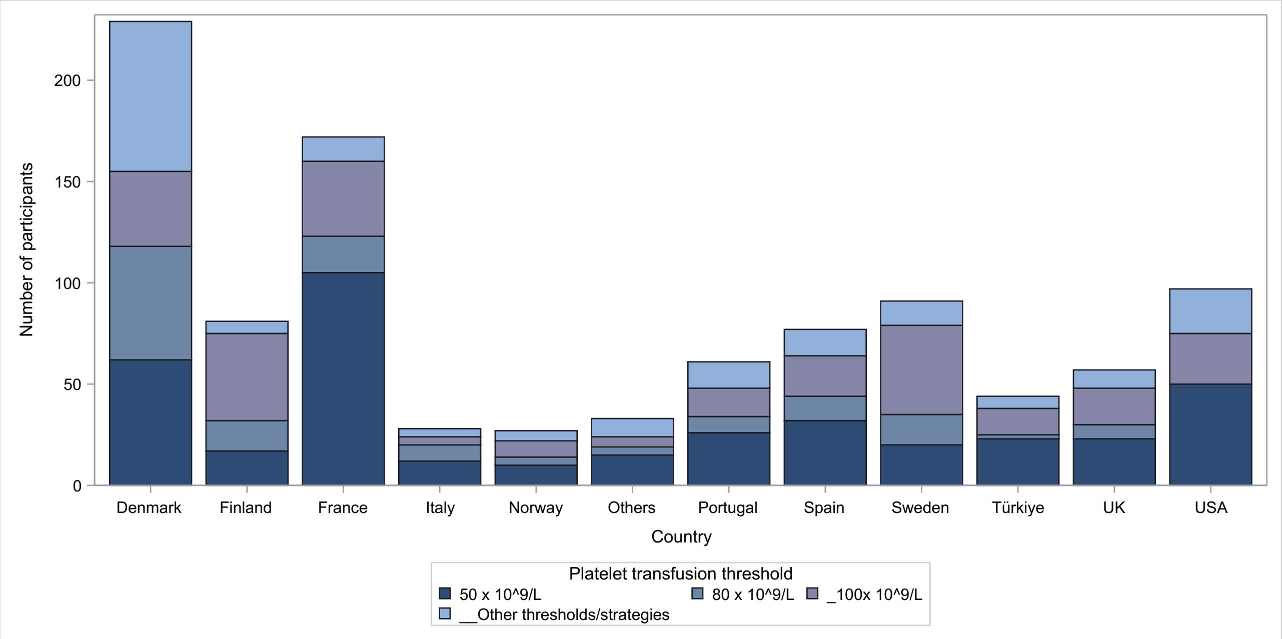

Thresholds preferred by less than 5% of respondents are pooled in the figure as 'Other thresholds'. Table S12 shows all responses.

Respondents' evaluation of coagulation in thrombocytopenic patients with bleeding

Table S13: Responses to the question 'Which blood tests do you use to evaluate coagulation in thrombocytopenic patients with MINOR bleeding?'

BLOOD TEST	USE	DO NOT USE
**PLATELET COUNT**	912 (91.5)	85 (8.5)
**HAEMOGLOBIN**	639 (64.1)	358 (35.9)
**FIBRINOGEN**	720 (72.2)	277 (27.8)
**PT/INR**	808 (81.0)	189 (19.0)
**APTT**	725 (72.7)	272 (17.3)
**TEG/ROTEM**	370 (62.9)	627 (37.1)
**MEA (MULTIPLATE)**	89 (8.9) [a)]	908 (91.1)

Data are in numbers (percentages)

[a)] Of the 89 respondents who replied that they used MEA, 67 (75%) were from Denmark.

Table S14: Responses to the question 'Which blood tests do you use to evaluate coagulation in thrombocytopenic patients with MAJOR bleeding?'

BLOOD TEST	USE	DO NOT USE
**PLATELET COUNT**	910 (91.3)	87 (8.7)
**HAEMOGLOBIN**	742 (74.4)	255 (25.6)
**FIBRINOGEN**	869 (87.2)	128 (12.8)
**PT/INR**	865 (86.8)	132 (13.2)
**APTT**	810 (81.2)	187 (18.8)
**TEG/ROTEM**	605 (60.7) [a)]	392 (39.3)
**MEA (MULTIPLATE)**	118 (11.8) [b)]	879 (88.2)

Data are in numbers (percentages)

[a)] Of the 605 respondents who used TEG, 229 (38%) were from Denmark: 225/229(98%) of Danish physicians replied they used TEG

b) Of the 118 respondents who replied that they used MEA, 79/118 (67%) were from Denmark

USE OF COAGULATION BLOOD TESTS TO EVALUATE COAGULATION IN PATIENTS WITH BLEEDING PER COUNTRY

A: Figures illustrating the number of respondents who use platelet count to evaluate coagulation in thrombocytopenic ICU patients with bleeding

FIG S13A: Use of platelet count in minor bleeding *(1: Do use, 0: Do not use)*

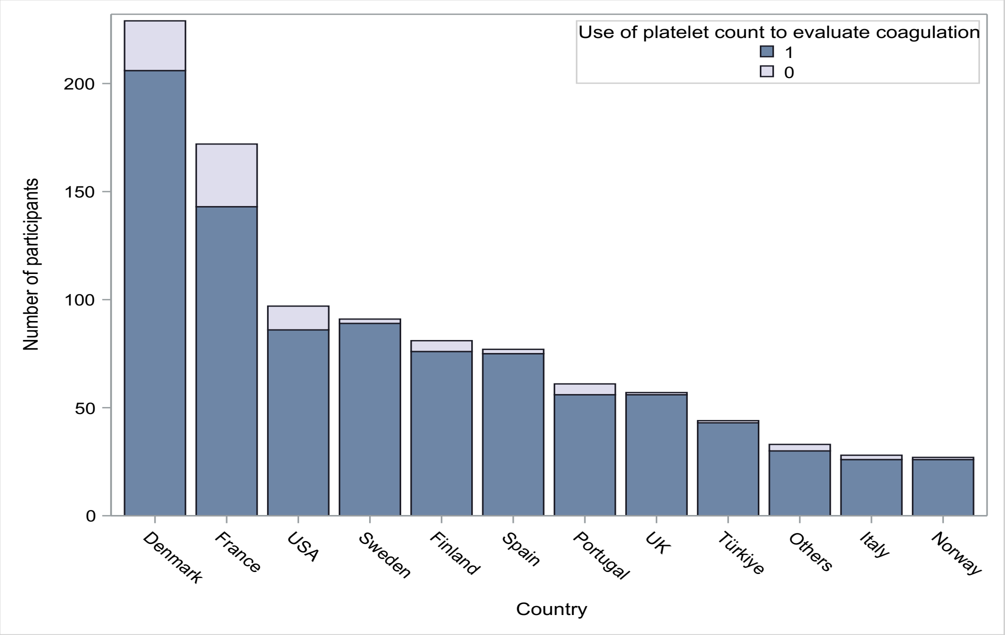

FIG S13B: Use of platelet count in major bleeding *(1: Do use, 0: Do not use)*

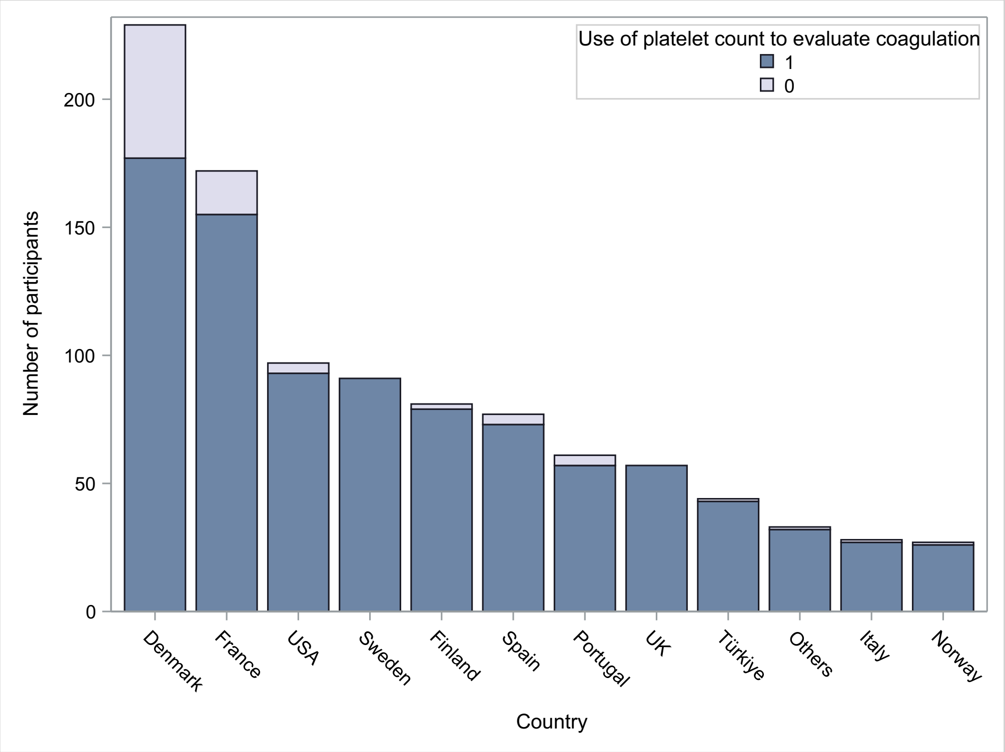

B: Figures illustrating the number of respondents who use the haemoglobin value to evaluate coagulation in thrombocytopenic ICU patients with bleeding

FIG S14A: Use of haemoglobin value in minor bleeding *(1: Do use, 0: Do not use)*

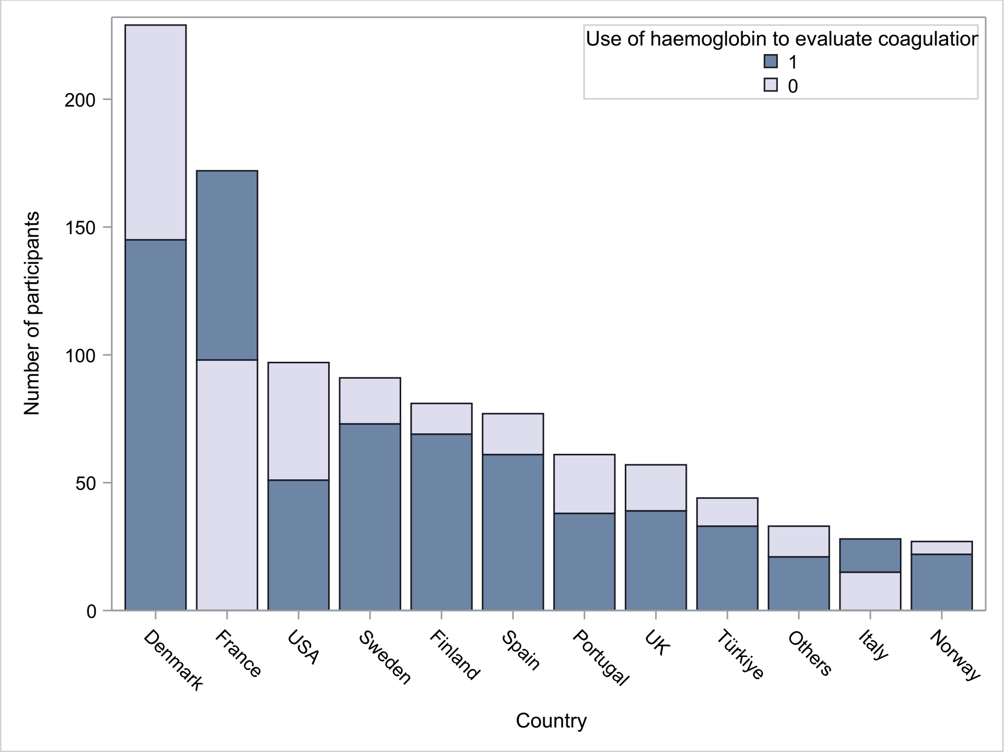

FIG S14B: Use of haemoglobin value in major bleeding *(1: Do use, 0: Do not use)*

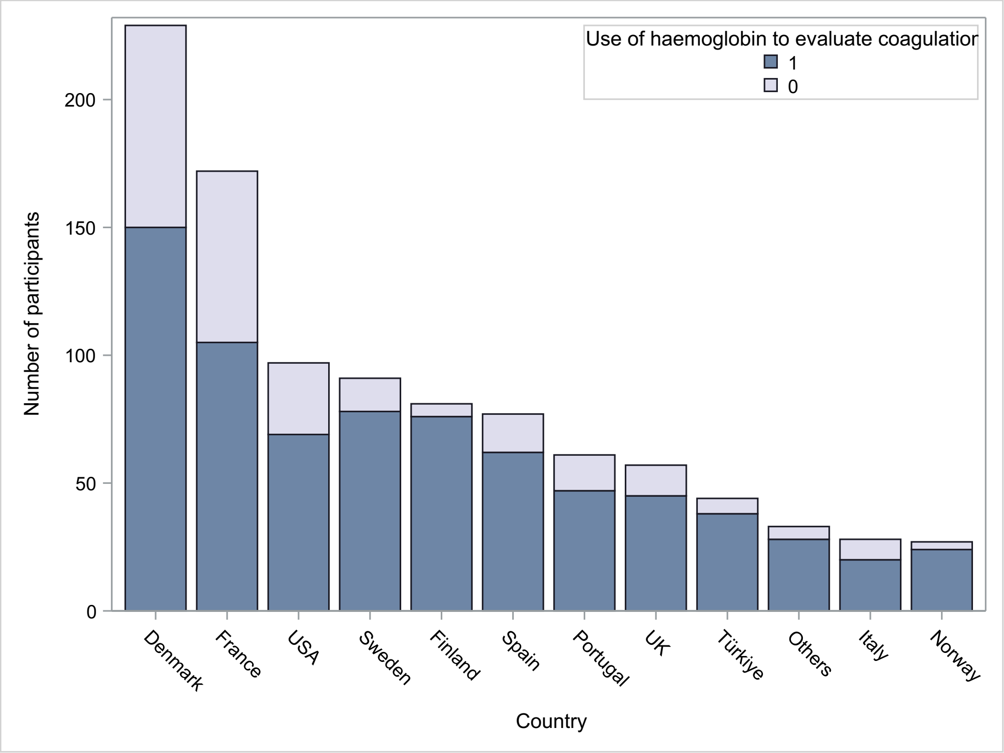

C: Figures illustrating the number of respondents who use fibrinogen to evaluate coagulation in thrombocytopenic ICU patients with bleeding

FIG S15A: Use of fibrinogen in minor bleeding *(1: Do use, 0: Do not use)*

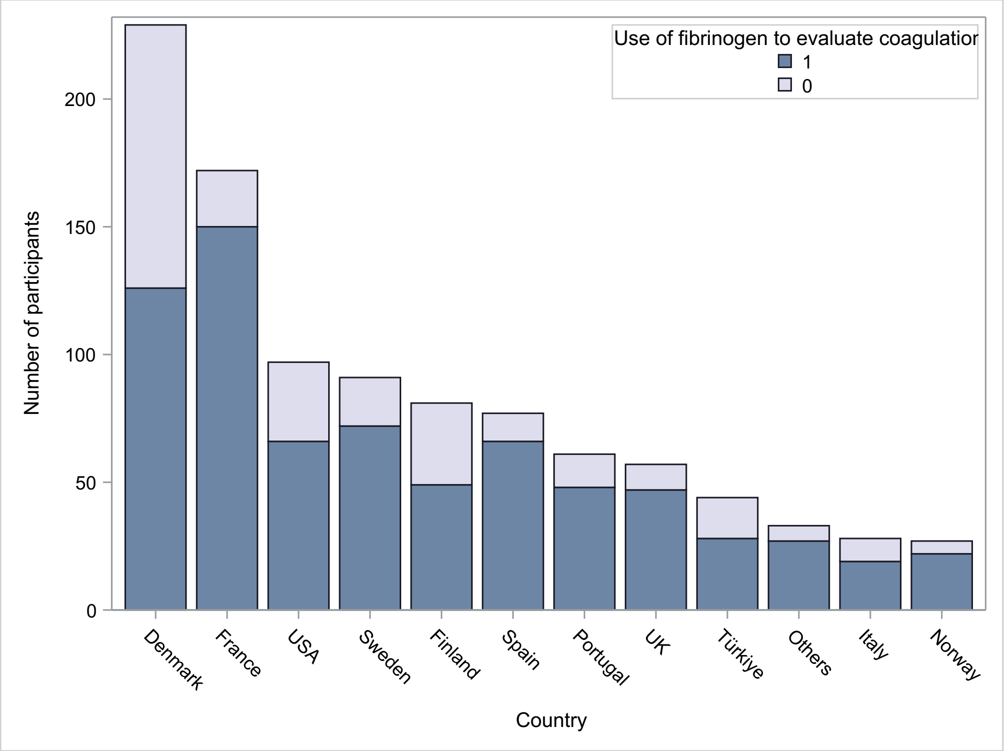

FIG S15B: Use of fibrinogen in major bleeding *(1: Do use, 0: Do not use)*

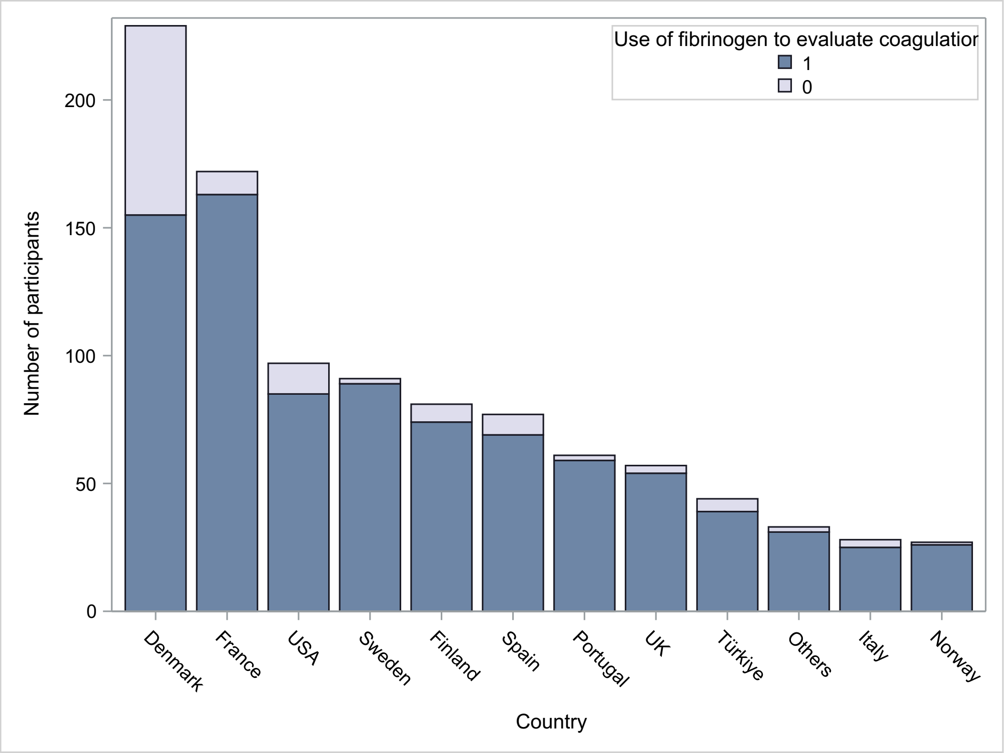

D: Figures illustrating the number of respondents who use *Prothrombin time (PT) / International normalised ratio (INR)* to evaluate coagulation in thrombocytopenic ICU patients with bleeding

FIG S16A: Use of PT/INR in minor bleeding *(1: Do use, 0: Do not use)*

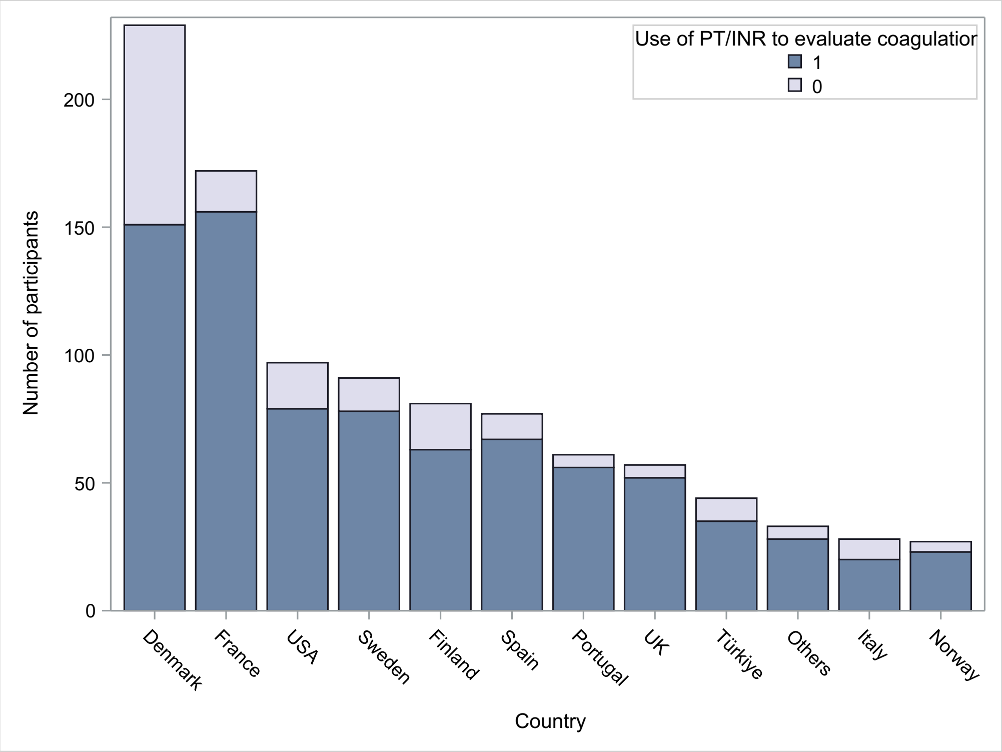

FIG S16B: Use of PT/INR in major bleeding *(1: Do use, 0: Do not use)*

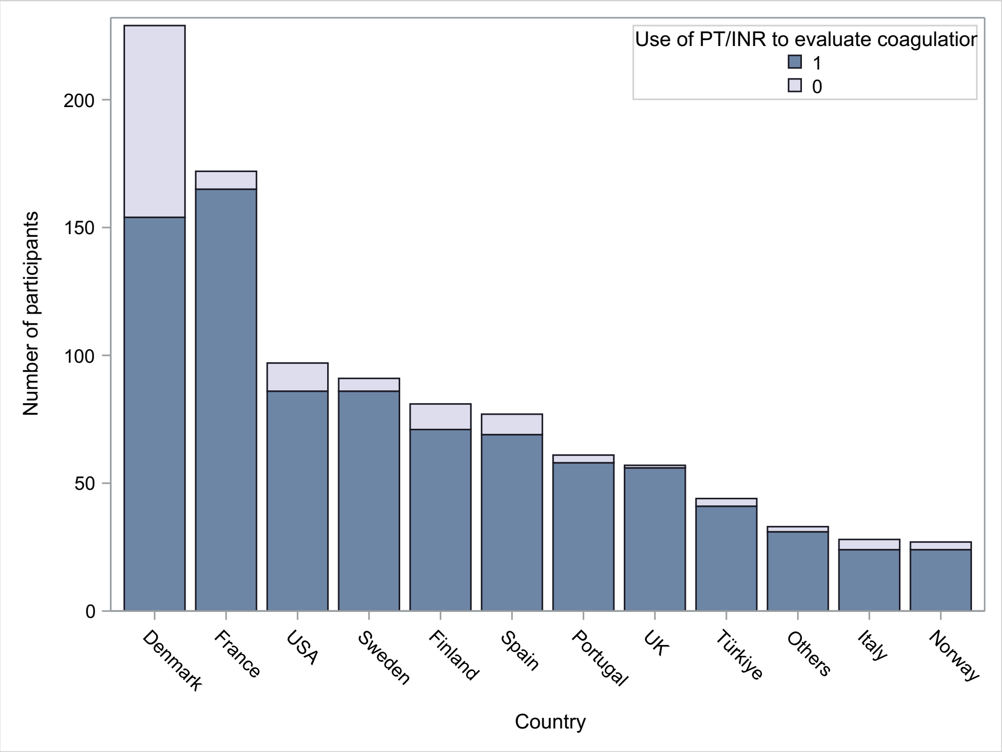

E: Figures illustrating the number of respondents who use *Activated partial thromboplastin time (aPTT)* to evaluate coagulation in thrombocytopenic ICU patients with bleeding

FIG S17A: Use of aPTT in minor bleeding *(1: Do use, 0: Do not use)*

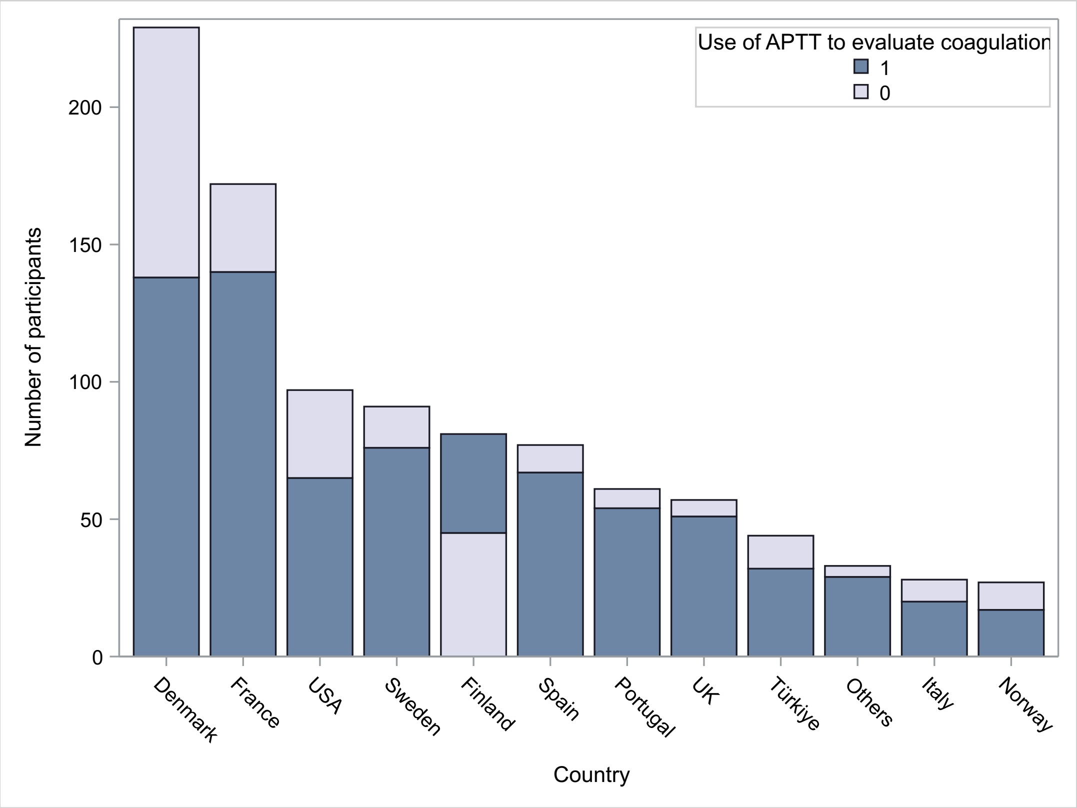

FIG S17B: Use of aPTT in major bleeding *(1: Do use, 0: Do not use)*

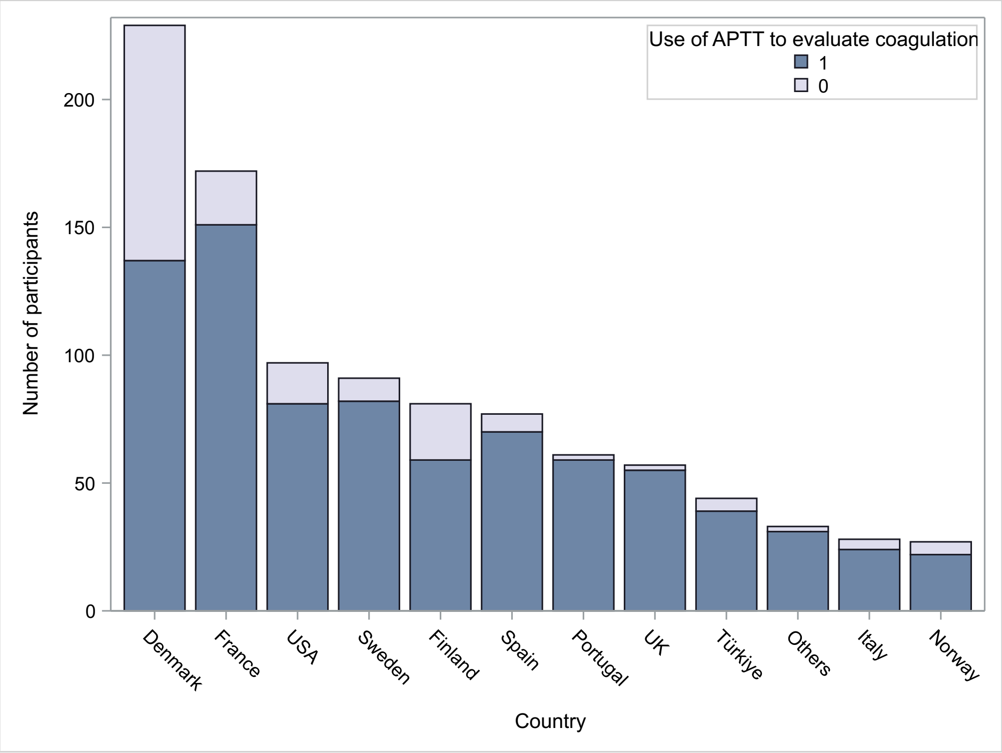

F: Figures illustrating the number of respondents who use Thromboelastography (TEG)/Rotational thromboelastometry (ROTEM) to evaluate coagulation in thrombocytopenic ICU patients with bleeding

FIG S18A: Use of TEG/ROTEM in minor bleeding *(1: Do use, 0: Do not use)*

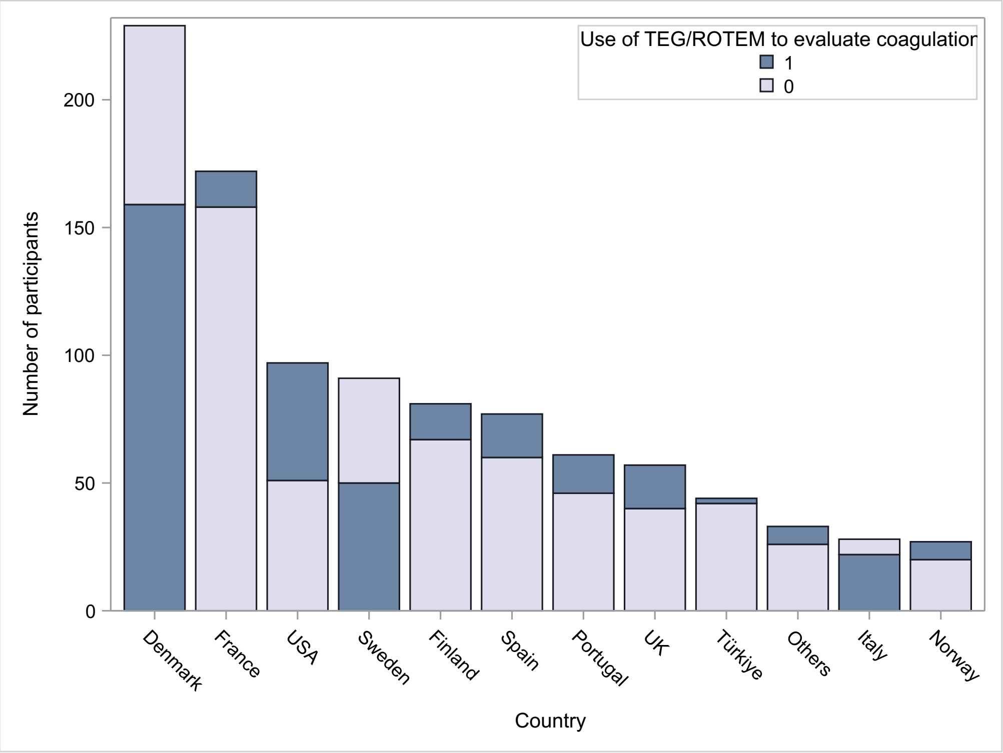

FIG S18B: Use of TEG/ROTEM in major bleeding *(1: Do use, 0: Do not use)*

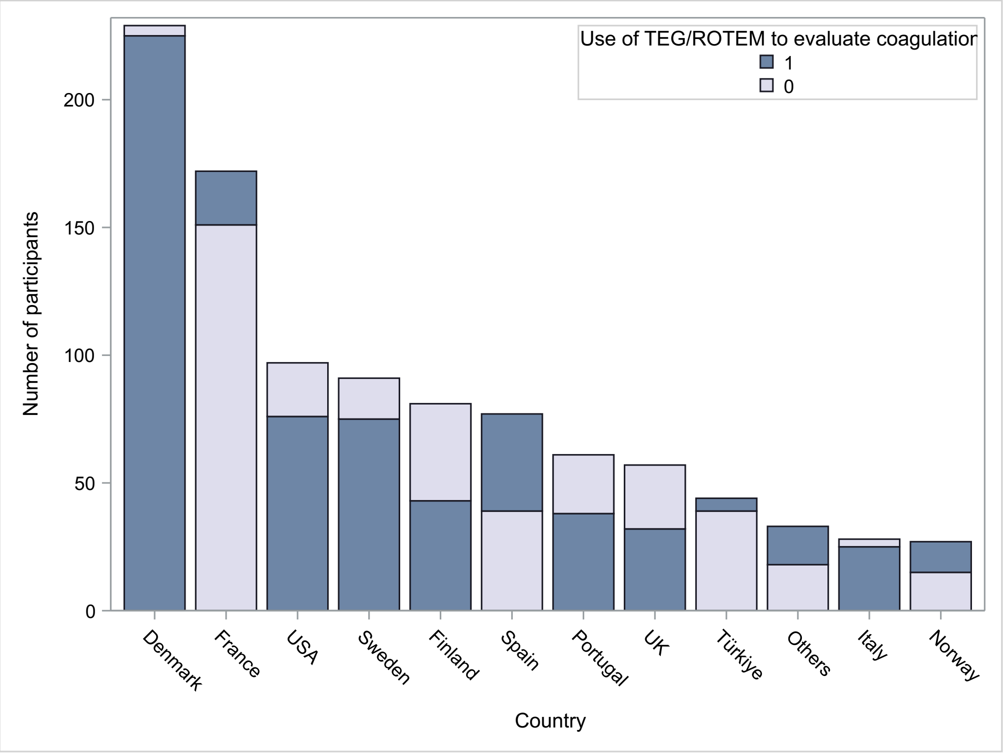

F: Figures illustrating the number of respondents who use Multiple electrode aggregometry (e.g. Multiplate Analyzer) to evaluate coagulation in thrombocytopenic ICU patients with bleeding

FIG S19A: Use of MEA in minor bleeding *(1: Do use, 0: Do not use)*

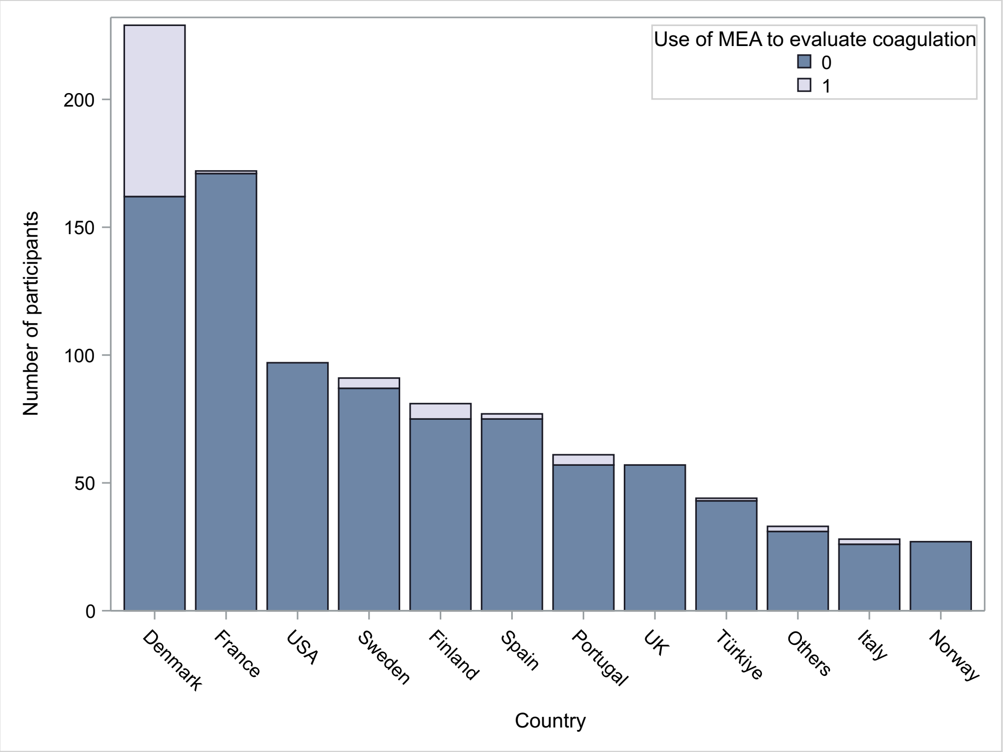

FIG S19B: Use of MEA in major bleeding *(1: Do use, 0: Do not use)*

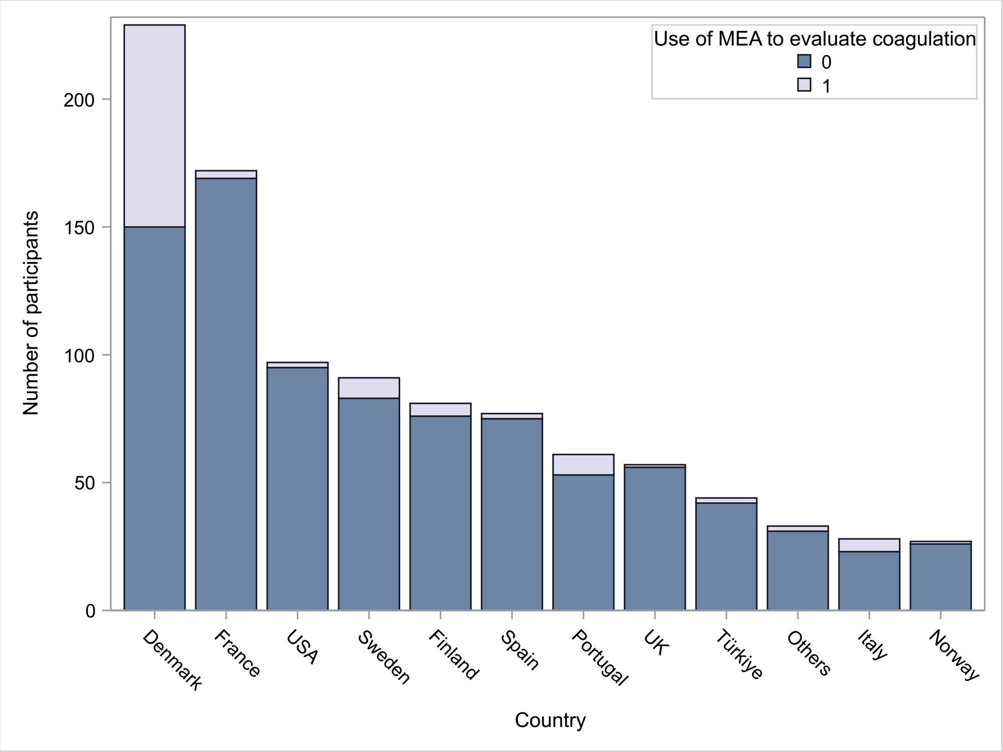

Selected quotes illustrating the most common responses to why physicians believe blood products are different from other treatments they prescribe to their patients, about the presence of unknown elements in blood transfusions, and about social influencers on decision-making

**Table S15: Reasons why physicians believe blood products are different from other treatments they prescribe to their patients**
**1. It is a rare resource**
It's a donation, so there is a limited amount available, and it should be prescribed with care; side effects may differ from transfusion to transfusion
All blood products are a precious commodity that are given to us out of charity and beneficence towards others. We should be stewards of their use as there is an implicit contract between those who donated and those who prescribe. Donors should feel confident that each transfusion is given after thoughtful reflection of its need and possible benefit.
They are limited by donations, there are often emotions around this from the patients and their families, donors would likely feel distress if their donations were used unwisely
They are donated by the public free of charge and can be each blood product can be viewed as gift. The risk of incompatible blood transfusion makes the level of process, systems checks and vigilance much greater for these products versus other therapies
They are a product of an altruistic donation (in my country) and they are expensive. Also, they are associated with major adverse events
Because stocks are, in general, more limited than of other treatments - there is a more clear potential loss to another patient from a transfusion that is not indicated, in the form of depletion of available blood products
Parce qu'ils viennent de donneurs volontaires qui ont pris du temps pour leur don; qu'ils sont chers et rares. Ces PSL méritent donc une grande vigilance dans les indications et leurs prescriptions. (Eng: Because they come from voluntary donors who have taken the time to donate; because they are expensive and rare. They therefore deserve great vigilance in their indications and prescriptions.
Ressource coûteuse, limitée, souvent sur-prescrite, dont les stocks s'affaiblissent depuis la pandémie COVID, et avec des risques sous-estimés. (Eng: A costly, limited resource, often over-prescribed, with dwindling stocks since the COVID pandemic, and with underestimated risks.)
**2. It is living material**
Transplant of cells is not totally equivalent to the patients having in mind that the majority of the ICU patients are immunesuppresssed due to their critical illnesses
I view the use of blood products as a transplant and as a result I'm more diligent with the use
Every blood transfusion is also an organ transplant. It has many side effects, it is costly and it should not be done in unnecessary situations
They are donated human substances. You can give Y kromosomes to women… We do not really know what reactions the components from other persons can start in the body
**3. It has immunological effects**
It causes exposure to an other person's imune system and probably a lot that we dont know about, long term effects.. or at least we don't see the impact in the ICU

Blood products affect not only the platelets or blood cells but the imune system and may have further effect on the patients future health and immune system
As a medicine, it is given in far greater volume, the sheer size of the medication (blood products) makes the fysiological changes far more prominent.. especiallay in patients with ongoing severe bleeding... even when we substitute the blood products.. a substitution of 75% or more of at patiens blood volume MUST also impact on the imune system as the patient will have virtually none of his/her original white leucocytes left; ; also the risk of immune system responce to the blood products must be considered and even if the risk is low, the risk of imunological complications is far greater than administering drugs such as paracetamol..
Risques immunologiques, immunisation HLA/HPA (eng: immunological risks, HLA/HPA immunisation)
**4. High risks of adverse effects**
Significant risk of infection and severe side effects such as TRALI
Blood products entail additional risks compared to other common therapies such as infection, volume overload, immunomodulation, and multiple types of immune-mediated adverse reactions. There are also religious and personal preferences to consider (and even more complexity with pediatric patients).
Effets secondaires propres, notamment infectieux et reactions transfusionnelles (eng: Specific side effects, particularly infectious and transfusion reactions)

**Table S16. Unknown elements in blood transfusions**
**1. Infections**
We cannot test viruses or particles we don't know yet..
While I think it is possible that there are transmittable things that we do not screen for (yet) that we will discover in the future, on the whole, blood transfusion is significantly safer than it was several decades ago with screening for infectious agents and things like prion disease
I witnessed the transfusion of hepatitis C, called non-A non-B for which there was no testing initially and even before LFTs were systematically checked. This can happen again with a new transmissible disease
There could be some unidentified viruses or prions or something else we can not yet find or don't know. There are multiple diseases that are shown to be at least partly by infectious origin and there may be more of them. They might spread through blood transfusion.
Viruses that have not been identified so far can be transferred (for years HCV was known as non-A/non-B hepatitis; HIV was probably spreading before we knew what it was). There is probably disease ongoing currently for which a potential viral cause has not been identified
Prions, virus, bacteria and immune components could all be transferred and it depends on how safe the system is
Tons of diseases are not tested well enough. We recently discovered malaria is not tested in our country, for example, which is surprising, as migration and world-wide travelling is usual nowadays
**2. Immunologically active components**
There are certain cut off levels that usually other doctors think transfusion is needed. Most think transfusion has almost no side effects. this causes pressure on the intensivist
We don't understand the immune system completely, and the scientific approach is: if it isn't proven its isn't non-existent, we just don't know. But there are no reasons to believe a transfusion transfers part of the soul or nano devices or something like that. Safety is proven by clinical trials
Immuno-depression induite, contamination bactérienne ... (eng: induced immunodepression, bacterial contamination)

All aspects are strongly influence by our local bloodbanks strong opinions, and we are guided by phone
**3. Unknown unknowns**
We should be humble regarding things we dont know....
Those would be unknown unknowns, but it is plausible that infectious agents can be transferred by blood products for which no screening is pursued. Furthermore, prionic diseases may still evade detection even for known diseases like Creutzfeld's.
Maybe that is possible as we don't know every adverse effect of every treatment. We don't neccesairly know everything that happens with every medication we give the patients. It is the same with blood-products
I think there are still much things we do not know or have not been defined in modern medicine, and we learn new things every day. For this reason, things that we still cannot define but exist there may be transferred

**Table S17. Social influencers on decision making**
**1. The culture in the department**
We tend to do as we have always done, although we lack evidence to support some of it
There is often very little literature to support transfusion limits in either direction. This often leads to culture based treatments "we use to transfuse when this and this and this..."; Sometimes the pendulum swings the other way and then, it is suddenly a "no-go" to transfuse blood cells unless the patient is almost clinically deranged..
We always use to give 2 bags of platelets. It is difficult to chance an ongoing practice if it is not written in a specific guideline.
Culture that influences my views has to do with the kind of patients that are admitted in the ICU I work. Since it is a medical ICU with a lot of hemato-oncologic patients, I have a more restrictive culture. If my patients were trauma or surgical, I would probably be less restrictive
I work in transfusion-liberal ICU. I figure we could improve treatment
Transfusion levels are very influenced by the culture at the hospital. The culture-level [which we transfuse at] at our hospital is higher than what I believe the international recommendations are
I'm currently changing work place a lot. Based on that, I can see different cultures and departments adhering to different guidelines or local customs. For now I follow the guidelines provided where I work.
I think transfusion practices in my department or hospital is partially cultural. What are traditional transfusion triggers here? If guidelines are updated it trickles down over years to include all physician, some probably never. Norwegians are generally positive to transfusions, and the blood bank seems to be functioning well
We'r quite liberal, are allowed to decide ourselves and there less unwritten rules than in neighboring countries. Although legal issues can be severe in the Netherlands I think healthcare workers have a lot of room to maneuver in.
Culture inquiète du service et on transfuse dès que le taux de plaquette est en dessous de 20 G/l chez les patients à faible risque hémorragique alors que je pense que 10 G/l est un seuil raisonnable chez ces patients. (eng: There is an anxious culture/attituden in the department, transfuses as soon as the platelet count falls below 20 G/l in patients at low risk of haemorrhage, whereas I think that 10 G/l is a reasonable threshold for these patients.)
Formation hématologue avec beaucoup de transfusion dans le service d'hématologie et actuellement dans un service de réanimation qui freine l'utilisation des produits sanguins. (Eng: Trained as a haematologist with a lot of transfusions in the haematology department and currently in an intensive care unit, where the use of blood products is restricted.)
**2. Colleagues views**

There are certain cut off levels that usually other doctors think transfusion is needed. Most think transfusion has almost no side effects. this causes pressure on the intensivist
All of my medical practice is heavily influenced by my colleagues and the local practices of the department, hospital and country, this includes blood transfusions
I am influenced by surgeon and anesthesia colleagues' preferences
This is common practice in the hospital/surgical unit/ICU.. When you are young, you "do as you are told" by superior/more experienced doctors
General practice and superior doctors' opinion affects transfusion policies. Defensive medicine and the risk of complaints, due to conservative recommendations
You learn from older colleges
All aspects are strongly influence by our local bloodbanks strong opinions, and we are guided by phone
**3. Religion**
Some religions don't allow transfusions and that limits our treatment options
I see patients of different cultures and beliefs, particularly Jehovah's witnesses, which impact how I approach transfusion of all blood products
Patients' religious beliefs and the shared decision making model in the US, which is different from South America, for example.
**4. Viewing blood products as a limited resource**
We are used to relate blood products to a limited product which depends on human donors and we associate blood transfusion to be harmful or to haver severe side effects
We do not have a full blood bank in our hospital. We have stockage of RBCs and FFPs, but not of platelets. They have to be transported from 75km away, so this is definitely a factor influencing our decision, particularly for prophylactic administration.
Je crois que la disponibilité des produits sanguins influence la générosité de leur prescription. J'ai constaté beaucoup de surtransfusion dans les centres universitaires de grande taille (Eng: I believe that the availability of blood products influences the generosity of their prescription. I've seen a lot of over-transfusion in large university centres.)
Informations récurrentes sur le risque de pénurie de sang, et situation déjà vécu de pénurie avec limitation de la stratégie transfusionnelle. (Eng: Recurrent information on the risk of a blood shortage, and a situation of shortage already experienced, limiting the transfusion strategy.)

Survey respondents' preferences if participating in a Randomised Clinical Trial (RCT) on platelet transfusions

Table S18: Respondents' replies to the question: "Would you be willing to randomise patients with thrombocytopenia to different management strategies in a future randomised clinical trial (RCT) on platelet transfusions?

Reply	Number
Yes	478 (47.94%)
Yes, maybe	405 (40.62%)
Probably not	91 (9.13%)
No	23 (2.31%)

Fig S20. The number of responses to the question "Would you be willing to randomise patients with thrombocytopenia to different management strategies in a future randomised clinical trial (RCT) on platelet transfusions?

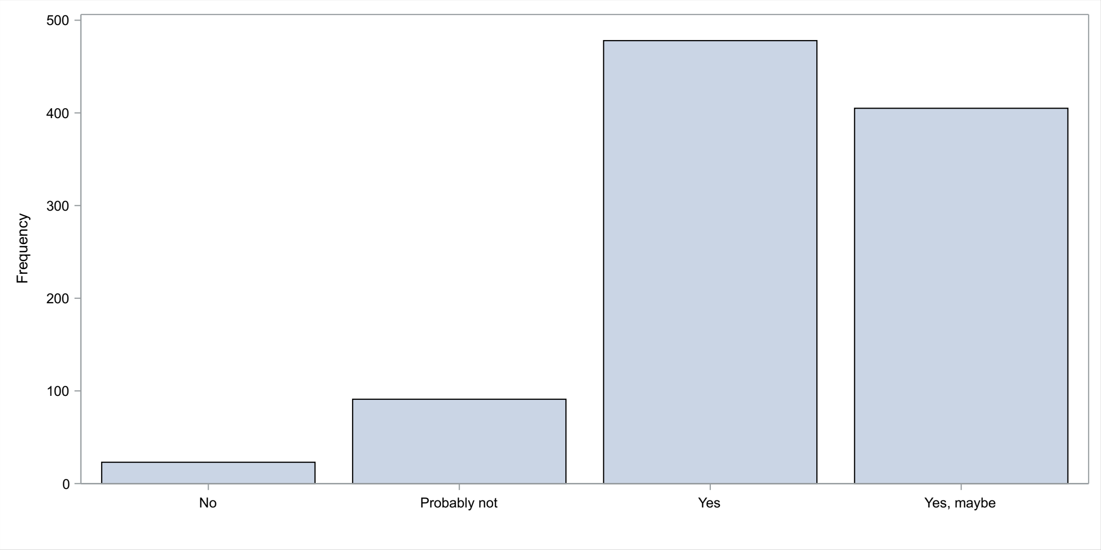

Table S19 Respondents' replies to the question: If you were to participate in an RCT – which transfusion protocol would you prefer?

Reply	Number
Two-level protocol [a)]	282 (28.28 %)
Three-level protocol [b)]	676 (67.80 %)
No opinion/unspecified/other	15 (1.51 %)
Do not wish to participate	24 (2.41%)

a) One transfusion threshold for low (normal) risk of bleeding and one threshold for high risk of bleeding or ongoing bleeding

b) One transfusion threshold for low (normal) risk of bleeding, one threshold for high risk of bleeding and one threshold for ongoing bleeding

Table S20. Respondents' replies to the question: If you were to participate in an RCT – which transfusion protocol (low vs high) would you find acceptable in ICU patients with a low risk of bleeding?

Reply	Number
Platelet transfusion threshold of:	
- 10 x 10^9/L vs 20 x 10^9/L	283 (28.39 %)
- 10 x 10^9/L vs 30 x 10^9/L	142 (14.24 %)
- 10 x 10^9/L vs 40 x 10^9/L	102 (10.23 %)
- 20 x 10^9/L vs 40 x 10^9/L	42 (4.21 %)
- 20 x 10^9/L vs 50 x 10^9/L	114 (11.43 %)
No transfusion vs 10 x 10^9/L	262 (26.28 %)
No opinion/unspecified/other	28 (2.87 %)
Do not wish to participate	24 (2.41%)

Figure S21: Respondents' replies to the question: If you were to participate in an RCT – which transfusion protocol (low vs high) would you find acceptable in ICU patients with a low risk of bleeding?

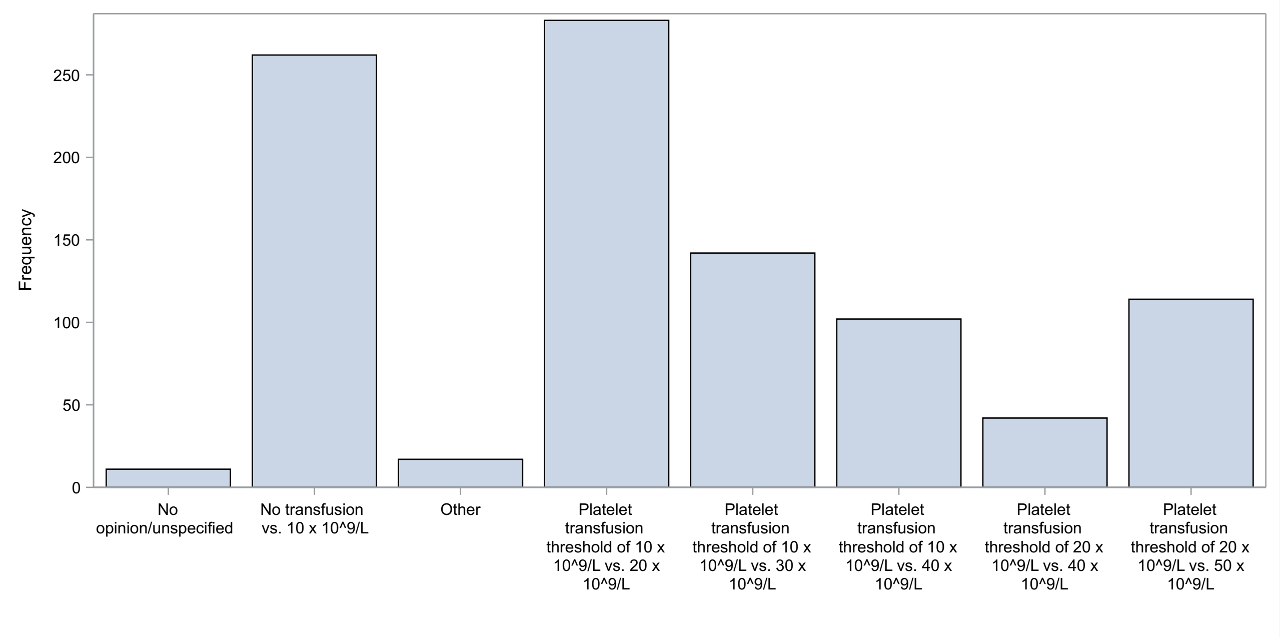

Table S21. Respondents' replies to the question: If you were to participate in an RCT – which transfusion protocol (low vs high) would you find acceptable in ICU patients with a high risk of bleeding?

Reply	Number
I would not include patients with high risk of bleeding in a trial	71 (7.12 %)
Platelet transfusion threshold of:	
- 10 x 10^9/L vs 20 x 10^9/L	78 (7.82%)
- 10 x 10^9/L vs 30 x 10^9/L	71 (7.12%)
- 10 x 10^9/L vs 40 x 10^9/L	80 (8.02 %)
- 20 x 10^9/L vs 40 x 10^9/L	75 (7.52 %)
- 20 x 10^9/L vs 50 x 10^9/L	325 (32.60 %)
- 30 x 10^9/L vs 50 x 10^9/L	241 (24.17 %)
No opinion/unspecified/other	32 (3.28 %)
Do not wish to participate	24 (2.41%)

Figure S22. **Respondents' replies to the question: If you were to participate in an RCT – which transfusion protocol (low vs high) would you find acceptable in ICU patients with a high risk of bleeding?**

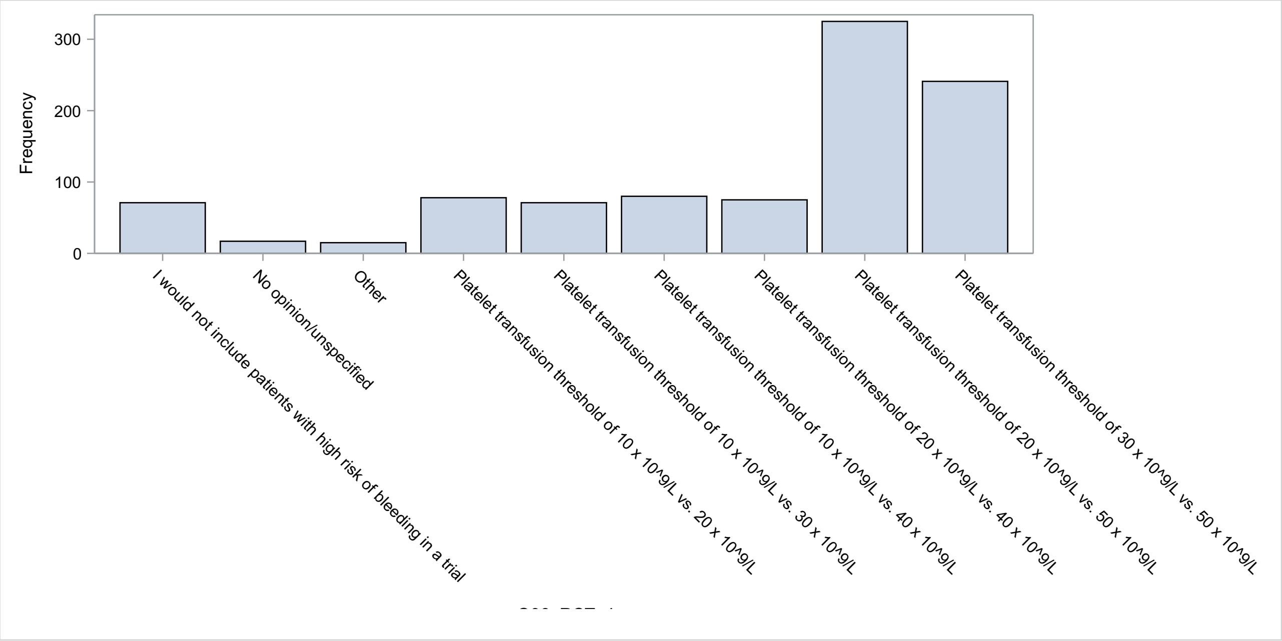

Table S22. Respondents' replies to the question: If you were to participate in an RCT – which transfusion protocol (low vs high) would you find acceptable in ICU patients with ONGOING bleeding?

**Reply**	**Number**
I would not include patients with ongoing bleeding in a trial	126 (12.64)
Platelet transfusion threshold of:	
- 10 x 10^9/L vs 20 x 10^9/L	21 (2.11 %)
- 10 x 10^9/L vs 30 x 10^9/L	15 (1.50 %)
- 10 x 10^9/L vs 40 x 10^9/L	20 (2.01 %)
- 20 x 10^9/L vs 40 x 10^9/L	25 (2.51 %)
- 20 x 10^9/L vs 50 x 10^9/L	128 (12.84 %)
- 30 x 10^9/L vs 50 x 10^9/L	104 (10.43 %)
- 30 x 10^9/L vs 80 x 10^9/L	107 (10.73%)
- 50 x 10^9/L vs 80 x 10^9/L	383 (38.42 %)
No opinion/unspecified/other	44 (4.50 %)
Do not wish to participate	24 (2.41%)
