# Supplementary material for: Platelet transfusion practice in the intensive care unit: the Nine-I international platelet transfusion survey
Source: Ann Intensive Care. 2025 Jul 8;15:91. doi: 10.1186/s13613-025-01494-4 (PMC12237844; doi:10.1186/s13613-025-01494-4)
Supplement: Supplementary file 2 — Supplementary material 2. [file 13613_2025_1494_MOESM2_ESM.pdf]

SUPPLEMENTAL MATERIAL 2 – Invitation letter and surveys

1. English survey p. 2
2. French survey p. 18
3. Invitation letter p. 34
4. Information about the Nine-I Research Network p. 37

PLATELET TRANSFUSIONS IN THE ICU – A SURVEY

This survey consists of 7 sections and 43 questions. Please note that most questions only concern platelet transfusions, but section 6 is about your views on all blood transfusions.

Survey content:

1. Hospital and intensive care unit structure (7 questions)
2. Institutional protocols and guidelines for platelet transfusions (3 questions)
3. Prophylactic platelet transfusions in the ICU (14 questions)
4. Platelet transfusions in the bleeding ICU patients (4 questions)
5. Preferences for a platelet transfusion trial in the ICU (5 questions)
6. Personal views on blood product transfusions (4 questions)
7. Personal details (6 questions)

PART 1: HOSPITAL AND INTENSIVE CARE UNIT (ICU) STRUCTURE (7 questions)

1. Please select the best description for your hospital

- ○ University hospital (hospital directly affiliated with a university medical school and participates in teaching and research activities)
- ○ Community/regional/district hospital-teaching (hospital affiliated with university medical school and provides regular teaching activity)
- ○ Community/regional/district hospital-nonteaching (hospital not affiliated with university medical school and does not usually provide teaching activities)
- ○ Other (please specify): ________________________________

2. Please specify the country where you practice

- ○ Scroll-down list of countries.

3. What is the structure of funding for your hospital?

- ○ Private
- ○ Public
- ○ Mixed
- ○ Other (please specify): ________________________________

4. Has your hospital a department (non-ICU) specific for haematology patients?

- ○ Yes
- ○ No

5. Has your hospital got a department (non-ICU) specific for oncology patients?

- ○ Yes
- ○ No

6. How many beds are in your ICU?

- ○ <10
- ○ 10-19
- ○ 20-29
- ○ 30-39
- ○ 40 or more

7. What types of patients are admitted to your ICU? (Please select all that apply)

- ○ Medical patients
- ○ Surgical patients (including trauma patients)
- ○ Neurosurgery patients
- ○ Cardio-thoracic surgery patients

- ○ Haematological patients
- ○ Oncological patients
- ○ Burn patients
- ○ Solid organ transplant patients
- ○ Haematopoetic stem cell transplant patients
- ○ ECMO/ECLS patients

PART 2: INSTITUTIONAL PROTOCOLS AND GUIDELINES (3 questions)

8. Do you have a platelet transfusion protocol in your hospital?

- ○ Yes
- ○ No
- ○ I do not know

9. Do you have an ICU-specific platelet transfusion protocol?

- ○ Yes
- ○ No
- ○ I do not know

10. Who can prescribe a platelet transfusion in your ICU? (Please select all that apply)

- ○ ICU specialist
- ○ Haematologist
- ○ Dedicated transfusion medicine specialist
- ○ Resident (specialist in training)/junior doctor/house officer / intern
- ○ Nurse
- ○ Other, please specify: ____

PART 3: PROPHYLACTIC PLATELET TRANSFUSIONS IN THE ICU ***(14 questions)***

11. In general, at what platelet transfusion threshold would you give a prophylactic platelet transfusion in a thrombocytopenic **non-bleeding medical** ICU patient?

- I do not use prophylactic platelet transfusions
- 10×10^9/L
- 20×10^9/L
- 30×10^9/L
- 40×10^9/L
- 50×10^9/L
- Other, please specify ______________________________

12. If the medical patient in the previous question had **hypoproliferative thrombocytopenia (bone-marrow failure)**, would that change your strategy?

- Yes
- No

If yes:
12 b) How would the presence of hypoproliferative thrombocytopenia change your management?

- I would be more liberal with platelet transfusion
- I would be more restrictive with platelet transfusion
- Other, please specify

12 c) Why would you consider changing your management?

____

13. At what threshold would you give a prophylactic platelet transfusion in a thrombocytopenic **non-bleeding surgical ICU patient** (including **trauma patients**)?

- I do not use prophylactic platelet transfusions
- 10×10^9/L
- 20×10^9/L
- 30×10^9/L

- ○ 40 x 10^9/L
- ○ 50 x 10^9/L
- ○ 80 x 10^9/L
- ○ Other, please specify ____________________

14. What is your threshold for platelet transfusion in a non-bleeding thrombocytopenic patient **prior to placing a central line** in the ICU?

- ○ I do not use prophylactic platelet transfusions
- ○ 10 x 10^9/L
- ○ 20 x 10^9/L
- ○ 30 x 10^9/L
- ○ 40 x 10^9/L
- ○ 50 x 10^9/L
- ○ 80 x 10^9/L
- ○ Other, please specify ____________________

15. What is your threshold for platelet transfusion in non-bleeding thrombocytopenic ICU patients **prior to a bronchoscopy**?

- ○ I do not use prophylactic platelet transfusions
- ○ 10 x 10^9/L
- ○ 20 x 10^9/L
- ○ 30 x 10^9/L
- ○ 40 x 10^9/L
- ○ 50 x 10^9/L
- ○ 80 x 10^9/L
- ○ Other, please specify ____________________

16. What is your threshold for platelet transfusion in non-bleeding thrombocytopenic ICU patients **prior to a percutanous dilatation tracheostomy**?

- ○ I do not use prophylactic platelet transfusions

- ○ 10 x 10^9/L
- ○ 20 x 10^9/L
- ○ 30 x 10^9/L
- ○ 40 x 10^9/L
- ○ 50 x 10^9/L
- ○ 80 x 10^9/L
- ○ Other, please specify ____________________
- ○ We do not perform percutanous dilatation trachestomies in my ICU

17. What is your threshold for platelet transfusion in a thrombocytopenic non-bleeding ICU patient **prior to a lumbar puncture or epidural?**

- ○ I do not use prophylactic platelet transfusions
- ○ 10 x 10^9/L
- ○ 20 x 10^9/L
- ○ 30 x 10^9/L
- ○ 40 x 10^9/L
- ○ 50 x 10^9/L
- ○ 80 x 10^9/L
- ○ 100 x 10^9/L
- ○ 150 x 10^9/L
- ○ Other, please specify ____________________

18. What is your threshold for platelet transfusion in thrombocytopenic non-bleeding critically ill patients **prior to neurosurgery**? (10^9 cells/L)

- ○ I do not use prophylactic platelet transfusions
- ○ 10 x 10^9/L
- ○ 20 x 10^9/L
- ○ 30 x 10^9/L
- ○ 40 x 10^9/L
- ○ 50 x 10^9/L
- ○ 80 x 10^9/L
- ○ 100 x 10^9/L

- 150 x 10^9/L
- Other, please specify ____________________

19. To what extent do the following conditions or treatments **influence** your decision to initiate a prophylactic platelet transfusion?

Please choose 1 out of the five options for each condition; alternatively, choose “I do not know”.

1: No influence 2: Some influence 3: Moderate influence 4: Substantial influence 5: Complete influence

	1	2	3	4	5	I do not know
Fever						
Sepsis						
Shock						
Liver failure						
Mechanical ventilation						
Renal replacement therapy in the ICU						
Extracorporeal life support (ECLS) incl. ECMO						
Immunocompromised status						
Haematological malignancy						
Ongoing treatment with chemotherapy						
Angioinvasive fungal infection (pulmonary or cerebral)						
Solid organ transplantation						
Presence of minor bleeding(s) (Equivalent to WHO grade 1-2)*						
Recent major bleeding (within 7 days)						
Recent intracerebral bleeding (within 7 days)						
Recent thrombosis						
Disseminated intravascular coagulation (DIC)						
Platelet availability alerts						

Patient's wishes						
Family wishes						
Conflict with the family						

* WHO I-II: non-CNS-bleeding not requiring transfusion

20. To what extent do the following **biomarkers and coagulation tests** influence your decision to initiate a prophylactic platelet transfusion? (Please choose 1 out of the five options for each condition; alternatively chose "I do not know/do not use")

1: No influence 2: Some influence 3: Moderate influence 4: Substantial influence 5: Complete influence

	1	2	3	4	5	I do not use
Haemoglobin level						
Leukocyte count						
Fibrinogen						
Prothrombin time (PT)/International Normalized Ratio (INR)						
C-Reactive Protein (CRP)						
Rotational thromboelastometry (ROTEM) or Thromboelastography (TEG)						
Multiple electric aggregometry (MEA) - Multiplate						
Other, please specify						

21. Do you check the platelet count after giving one platelet transfusion before giving a second platelet transfusion in the **non-bleeding** ICU patient?

- ○ Yes
- ○ No
- ○ Sometimes

22. Are you concerned about the side-effects of prophylactic platelet transfusions?

- ○ Always
- ○ Sometimes
- ○ Rarely
- ○ Never

If yes:

22 b). In general, on a scale from 1 to 5, how concerned are you about the following side effects when prescribing a platelet transfusion?

a) TRALI
b) ICU-acquired infections
c) Effects on the immune system
d) Risk of thrombosis
e) Fluid overload
f) Virus- or prion infections transmitted through the transfusion (hepatitis, HIV, Creutzfeldt-Jakob disease or similar)

not at all / 1 2 3 4 5 / very concerned

23. Over the last 10 times you provided patients with platelet transfusions, how many times were you confident about the clinical indication

- ○ All
- ○ Most
- ○ Half

- Some
- None
- I do not know

24. Written guidelines on platelet transfusion in the ICU based on best-practise and expert opinions have been published.

a) Do you read these guidelines?

- Always
- Sometimes
- Rarely
- Never
- I do not know

b) If you have read the guidelines - do you adhere to these guidelines?

- Always
- Sometimes
- Rarely
- Never
- I do not know

PART 4: PLATELET TRANSFUSIONS IN THE BLEEDING ICU-PATIENT (4 questions)

25. In general, at what platelet transfusion threshold would you give a platelet transfusion in thrombocytopenic ICU patients with **minor bleeding** (WHO grade I-II; non-CNS bleeding not requiring red blood cell transfusion)?

- I do not use prophylactic platelet transfusions
- 10 x 10^9/L
- 20 x 10^9/L
- 30 x 10^9/L
- 40 x 10^9/L
- 50 x 10^9/L

- ○ 80 x 10^9/L
- ○ 100 x 10^9/L
- ○ Other, please specify ______________________________

26. Which coagulation tests do you routinely use to evaluate coagulation status in a thrombocytopenic patient with **minor bleeding** (WHO I-II; not requiring transfusion with red blood cells)?
(Multiple answers possible)

- ○ None
- ○ Haemoglobin
- ○ Platelet count
- ○ Prothrombin time (PT) / International normalized ratio (INR)
- ○ Activated partial thromboplastin time (APTT)
- ○ Fibrinogen
- ○ Antithrombin
- ○ Rotational thromboelastometry (ROTEM)/Thromboelastography (TEG)
- ○ Multiple electric aggregometry (MEA) – Multiplate
- ○ Others, please specify______________________________

27. In general, at what platelet transfusion threshold would you give a platelet transfusion in thrombocytopenic ICU patients with **major bleeding** (WHO grade III-IV; requiring transfusion with red blood cells)?

- ○ I do not use prophylactic platelet transfusions
- ○ 10 x 10^9/L
- ○ 20 x 10^9/L
- ○ 30 x 10^9/L
- ○ 40 x 10^9/L
- ○ 50 x 10^9/L
- ○ 80 x 10^9/L
- ○ 100 x 10^9/L
- ○ Other, please specify ______________________________

28. Which coagulation tests do you routinely use to evaluate coagulation status in a thrombocytopenic patient with **major bleeding** (WHO III-IV; bleeding requiring transfusion with red blood cells)?
(Multiple answers possible)

- ○ None
- ○ Haemoglobin
- ○ Platelet count
- ○ Prothrombin time (PT)/ International normalized ratio (INR)
- ○ Activated partial thromboplastin time (APTT)
- ○ Fibrinogen
- ○ Antithrombin
- ○ Rotational thromboelastometry (ROTEM) / Thromboelastography (TEG)
- ○ Multiple electric aggregometry (MEA) – Multiplate
- ○ Others, please specify____

PART 5: PLATELET TRANSFUSION TRIAL

(5 questions)

29. Would you be willing to randomise patients with thrombocytopenia to different management strategies in a future randomised clinical trial (RCT)?

- ○ Yes
- ○ Yes, maybe
- ○ Probably not
- ○ No

30. If you were to participate in a RCT – which transfusion protocol would you prefer?

- ○ Two-level protocol:
  One transfusion threshold for low (normal) risk of bleeding and one threshold for high risk of bleeding or ongoing bleeding
- ○ Three-level protocol:
  One transfusion threshold for low (normal) risk of bleeding, one threshold for high risk of bleeding and one threshold for ongoing bleeding
- ○ Other, please specify:
  ____
  ____

—

31. If you were to participate in an RCT: Which platelet transfusion threshold (low vs high) would you find acceptable in ICU patients with a **low risk of bleeding**?

- ○ No transfusion vs. 10×10^9/L
- ○ Platelet transfusion threshold of 10×10^9/L vs. 20×10^9/L
- ○ Platelet transfusion threshold of 10×10^9/L vs. 30×10^9/L
- ○ Platelet transfusion threshold of 10×10^9/L vs. 40×10^9/L
- ○ Platelet transfusion threshold of 20×10^9/L vs. 40×10^9/L
- ○ Platelet transfusion threshold of 20×10^9/L vs. 50×10^9/L
- ○ Other, please specify:

____

32. If you were to participate in an RCT: Which platelet transfusion threshold (low vs high) would you find acceptable in ICU patients with a **high risk of bleeding?**

- ○ Platelet transfusion threshold of 10×10^9/L vs. 20×10^9/L
- ○ Platelet transfusion threshold of 10×10^9/L vs. 30×10^9/L
- ○ Platelet transfusion threshold of 10×10^9/L vs. 40×10^9/L
- ○ Platelet transfusion threshold of 20×10^9/L vs. 40×10^9/L
- ○ Platelet transfusion threshold of 20×10^9/L vs. 50×10^9/L
- ○ Platelet transfusion threshold of 30×10^9/L vs. 50×10^9/L
- ○ Other, please specify:

____

33. If you were to participate in an RCT: Which platelet transfusion threshold (low vs high) would you find acceptable in ICU patients with **ongoing bleeding**?

- ○ I would not include patients with ongoing bleeding in a trial
- ○ Platelet transfusion threshold of 10×10^9/L vs. 20×10^9/L
- ○ Platelet transfusion threshold of 10×10^9/L vs. 30×10^9/L
- ○ Platelet transfusion threshold of 10×10^9/L vs. 40×10^9/L
- ○ Platelet transfusion threshold of 20×10^9/L vs. 40×10^9/L
- ○ Platelet transfusion threshold of 20×10^9/L vs. 50×10^9/L

- ○ Platelet transfusion threshold of 30 x 10^9/L vs. 50 x 10^9/L
- ○ Platelet transfusion threshold of 30 x 10^9/L vs. 80 x 10^9/L
- ○ Platelet transfusion threshold of 50 x 10^9/L vs. 80 x 10^9/L
- ○ Other, please specify: ____

PART 6: PERSONAL VIEWS ON BLOOD PRODUCT TRANSFUSIONS (4 questions)

Please note that the following questions are about all blood transfusions, including red blood cells (RBC), fresh frozen plasma (FFP) and platelet concentrate.

34 a). Do you think blood products are different from other treatments we prescribe to our patients?

- ○ Yes
- ○ No

33 b) If yes, why is that?____

35. Do you think your views and experiences of blood transfusions is influenced by culture (in the hospital, in the department, in the society)?

- ○ Yes
  - Please specify what elements of your culture affect your views and experiences (key words): ________________________________
- ○ No
- ○ I do not know

36 a). Some patients believe that other unknown elements can be transferred through a blood transfusion. Do you think this is possible?

- ○ Yes, definitely
- ○ Yes, maybe
- ○ No, but I cannot rule it out
- ○ No, absolutely not
- ○ I do not know

36 b) If yes, could you give some examples?

___

37. Have you or one of your loved ones ever received a blood transfusion?

- ○ Yes
- ○ No
- ○ I do not know
- ○ I prefer not to answer

FINAL PART (7): DEMOGRAPHICS AND CONSENT

38. What is your primary medical speciality?

- ○ Intensive Care Medicine
- ○ Anaesthesiology
- ○ Haematology
- ○ Oncology
- ○ Pulmonology
- ○ Internal medicine
- ○ Neurology
- ○ Surgery
- ○ Other, please specify: ___

39. How many years have you been working in the ICU? ___

40. Your age? ___

41. Your sex?

- ○ Male
- ○ Female
- ○ Non-binary
- ○ Other
- ○ Prefer not to answer

42. Do you have religious beliefs?

- ○ Yes, and I am practising
- ○ Yes, but I am not practising
- ○ Not sure
- ○ No
- ○ Prefer not to answer

43. Any additional comments or reflections you would like to add:

____
____
____

THIS WAS THE LAST QUESTION. THANK YOU SO MUCH FOR YOUR HELP!

Enquête sur les transfusions plaquettaires en réanimation médicale

Cette enquête se compose de 7 sections et de 43 questions. Veuillez noter que la plupart des questions concerne les transfusions plaquettaires, la 6eme partie de cette enquête s'intéresse à votre opinion sur les transfusions sanguines en générale.

Contenu de l'enquête :

8. Organisation des hôpitaux et des unités de soins intensifs (7 questions)
9. Protocoles et recommandations pour les transfusions plaquettaires (3 questions)
10. Transfusions plaquettaires prophylactiques en réanimation médicale (14 questions)
11. Transfusions plaquettaires chez les patients présentant une hémorragie active en réanimation médicale (4 questions)
12. Collecte d'avis sur un futur essai clinique évaluant la transfusion plaquettaire en soins intensifs (5 questions)

    Opinions personnelles des médecins sur les transfusions de produits sanguins (4 questions)

13. Données demographiques (6 questions)

PARTIE 1 : ORGANISATION DES HOPITAUX ET DES UNITES DE SOINS INTENSIFS (7 questions)

1. Veuillez sélectionner la meilleure description de votre hôpital

   - ○ Hôpital universitaire (hôpital directement lié à une faculté de médecine et participant à des activités d'enseignement et de recherche)
   - ○ Hôpital communautaire/Centre hospitalier régional/Centre hospitalier universitaire (hôpital lié à une faculté de médecine et offrant une activité d'enseignement régulière)
   - ○ Hôpital communautaire/Centre hospitalier régional/Hôpital non universitaire (hôpital non lié à une faculté de médecine et n'offrant généralement pas d'activités d'enseignement)
   - ○ Autre (veuillez préciser):______________________________

2. Veuillez préciser le pays où vous pratiquez ?
   Liste déroulante des pays

3. Quelle est la structure de financement de votre hôpital ?

- ○ Privé
- ○ Publique
- ○ Mixte privé et public
- ○ Autre (veuillez préciser):____

4. Votre hôpital dispose-t-il d'un service d'hématologie (hors USI) ?

- ○ Oui
- ○ Non

5. Votre hôpital dispose-t-il d'un service d'oncologie (hors USI) ?

- ○ Oui
- ○ Non

6. De combien de lits dispose votre unité de soins intensifs ?

- ○ <10
- ○ 10-19
- ○ 20-29
- ○ 30-39
- ○ 40 ou plus

7. Quels sont les différents types de patients admis dans votre unité de soins intensifs ? (Plusieurs réponses possibles)

- ○ Patients médicaux
- ○ Patients chirurgicaux (y compris les patients traumatisés)

- ○ Patients de neurochirurgie
- ○ Patients de chirurgie cardio-thoracique
- ○ Patients d'hématologie
- ○ Patients d'oncologie
- ○ Patients brûlés
- ○ Patients transplantés d'organe solide
- ○ Patients allogreffés/autogreffés de cellules souches hématopoïétiques
- ○ Patients sous ECMO/ECLS

PARTIE 2 : PROTOCOLES ET RECOMMANDATIONS POUR LES TRANSFUSIONS PLAQUETTAIRES (3 QUESTIONS)

8. Avez-vous un protocole de transfusion de plaquettes dans votre hôpital ?
   - ○ Oui
   - ○ Non
   - ○ Je ne sais pas

9. Disposez-vous d'un protocole de transfusion de plaquettes spécifique aux soins intensifs ?
   - ○ Oui
   - ○ Non
   - ○ Je ne sais pas

10. Qui peut prescrire une transfusion de plaquettes dans votre unité de soins intensifs? (Plusieurs réponses possibles)
   - ○ Spécialiste en médecine intensive et reanimation
   - ○ Hématologue
   - ○ Médecin spécialiste responsable des transfusions
   - ○ Résident (spécialiste en formation)/Docteur junior/Chef de clinique des universites-assistant des hopitaux/interne
   - ○ Infirmière
   - ○ Autre, veuillez préciser : ____

PARTIE 3 : TRANSFUSIONS PLAQUETTAIRES PROPHYLACTIQUES EN REANIMATION MEDICALE (14 QUESTIONS)

11. En général, quel est pour vous le seuil de transfusion plaquettaire prophylactique chez les patients de réanimation médicale présentant une thrombocytopénie **sans syndrome hémorragique associé** ?

- ○ Je ne prescris pas de transfusions plaquettaires
- ○ 10 x 10^9/L
- ○ 20 x 10^9/L
- ○ 30 x 10^9/L
- ○ 40 x 10^9/L
- ○ 50 x 10^9/L
- ○ Autre, veuillez préciser: ____________________

12. Si le patient de la question précédente souffrait d'une **Thrombopenie centrale (thrombocytopénie hypoproliférative)**, liée a insuffisance medullaire, vous changerez votre stratégie ?

- ○ Oui
- ○ Non

Si oui :

12 b) En quoi la présence d'une thrombocytopénie hypoproliférative modifie-t-elle votre prise en charge?

- ○ Je prescrirais plus facilement les transfusions plaquettaires
- ○ Je restreindrais mes prescriptions de transfusion de plaquettaire.
- ○ Autre, veuillez préciser : ____________________

12 c) Pourquoi envisageriez-vous de changer votre stratégie transfusionnelle ?

____

13. Quel est pour vous le seuil de transfusion plaquettaire prophylactique chez **les patients de réanimation chirurgiacale** (y compris **les patients traumatisés**) présentant une thrombocytopénie sans syndrome hémorragique associé ?

- ○ Je ne prescris pas de transfusions plaquettaires
- ○ 10 x 10^9/L
- ○ 20 x 10^9/L
- ○ 30 x 10^9/L
- ○ 40 x 10^9/L
- ○ 50 x 10^9/L
- ○ 80 x 10^9/L
- ○ Autre, veuillez préciser :____

14. En unité de soins intensifs, quel est pour vous le seuil de transfusion plaquettaire chez les patients, nécessitant **un cathéter veineux central**, et présentant une thrombopénie sans syndrome hémorragique associé ?

- ○ Je ne prescris pas de transfusions plaquettaires
- ○ 10 x 10^9/L
- ○ 20 x 10^9/L
- ○ 30 x 10^9/L
- ○ 40 x 10^9/L
- ○ 50 x 10^9/L
- ○ 80 x 10^9/L
- ○ Autre, veuillez préciser : ____

15. En unité de soins intensifs, quel est pour vous le seuil de transfusion plaquettaire chez les patients, nécessitant **une fibroscopie bronchique**, et présentant une thrombopénie sans syndrome hémorragique associé ?

- ○ Je ne prescris pas de transfusions plaquettaires
- ○ 10 x 10^9/L
- ○ 20 x 10^9/L
- ○ 30 x 10^9/L
- ○ 40 x 10^9/L

- ○ 50 x 10^9/L
- ○ 80 x 10^9/L
- ○ Autre, veuillez préciser: ______________________________

16. En unité de soins intensifs, quel est pour vous le seuil de transfusion plaquettaire chez les patients, nécessitant **une trachéotomie percutanée**, et présentant une thrombopénie sans syndrome hémorragique associé ?
    - ○ Je ne prescris pas de transfusions plaquettaires
    - ○ 10 x 10^9/L
    - ○ 20 x 10^9/L
    - ○ 30 x 10^9/L
    - ○ 40 x 10^9/L
    - ○ 50 x 10^9/L
    - ○ 80 x 10^9/L
    - ○ Autre, veuillez préciser: ______________________________
    - ○ Nous ne réalisons pas de trachéotomie par dilatation percutanée dans mon unité de soins intensifs.
17. En unité de soins intensifs, quel est pour vous le seuil de transfusion plaquettaire chez les patients, nécessitant **une ponction lombaire ou une péridurale**, et présentant une thrombopénie sans syndrome hémorragique associé ?
    - ○ Je ne prescris pas de transfusions plaquettaires
    - ○ 10 x 10^9/L
    - ○ 20 x 10^9/L
    - ○ 30 x 10^9/L
    - ○ 40 x 10^9/L
    - ○ 50 x 10^9/L
    - ○ 80 x 10^9/L
    - ○ 100 x 10^9/L
    - ○ 150 x 10^9/L
    - ○ Autre, veuillez préciser: ______________________________
18. En unité de soins intensifs, quel est pour vous le seuil de transfusion plaquettaire chez les patients, nécessitant **une intervention neurochirurgicale**, et présentant une

thrombopénie sans syndrome hémorragique associé ?

- ○ Je ne prescris pas de transfusions plaquettaires
- ○ 10×10^9/L
- ○ 20×10^9/L
- ○ 30×10^9/L
- ○ 40×10^9/L
- ○ 50×10^9/L
- ○ 70×10^9/L
- ○ 100×10^9/L
- ○ 150×10^9/L
- ○ Autre, veuillez préciser: ____________________

19. Dans quelle mesure les conditions ou traitements suivants influencent-ils votre décision d'initier une transfusion plaquettaire prophylactique?
    Veuillez choisir une des cinq options pour chaque condition; vous pouvez également choisir "Je ne sais pas".

1 : Aucune influence 2 : Une influence minime 3 : Une influence modérée 4 : Une influence importante 5 : Une influence complète

	1	2	3	4	5	Je ne sais pas
Fièvre						
Sepsis						
Etat de choc						
Insuffisance hépatique						
Ventilation mécanique						
Epuration extra reanale en unite de soins intensifs						
Assistance respiratoire extracorporelle (ECLS), y compris ECMO						
Statut d'immunodéficience						

Hemopathie maligne						
Traitement en cours par chimiothérapie						
Infection fongique angioinvasive (pulmonaire ou cérébrale)						
Transplantation d'organes solides						
Présence de saignement(s) mineur(s) (équivalent au grade 1-2 de l'OMS)*.						
Hémorragie majeure récente (dans les 7 jours)						
Hémorragie intracérébrale récente (dans les 7 jours)						
Thrombose récente						
Coagulation intravasculaire disséminée (CIVD)						
Probleme de disponibilite de plaquettes						
Le souhait du patient						
Le souhait de la famille						
Les conflits avec les familles						

* OMS I-II : hémorragie sans hémorragie du système nerveux central ne nécessitant pas de transfusion.

20. Dans quelle mesure les **biomarqueurs et les tests de coagulation** suivants influencent-ils votre décision de prescrire une transfusion plaquettaire prophylactique ?

    (Veuillez choisir 1 des cinq options pour chaque condition ; vous pouvez également choisisir "Je ne sais pas/je ne prescris pas")

1 : Aucune influence 2 : Une influence minime 3 : Une influence modérée 4 : Une influence importante 5 : Une influence complète

	1	2	3	4	5	Je ne sais pas/Je ne prescris pas
Taux d'hémoglobine						
Taux de leucocytes						
Fibrinogène						
Temps de prothrombine (TP)/International Normalised ratio (INR)						
Protéine C-Réactive (CRP)						

Thromboélastométrie rotationnelle (ROTEM) ou Thromboélastographie (TEG)						
Agrégométrie électrique multiple (MEA) –Agrégométrie plaquettaire (Multiplate)						
Autre, veuillez préciser : ______________________						

21. En unité de soins intensifs, après avoir administré une première transfusion de plaquettes, chez un patient thrombopénique sans syndrome hémorragique associé, contrôlez-vous systématiquement la numération plaquettaire avant la deuxième transfusion de plaquettes ?

- ○ Oui
- ○ Non
- ○ Parfois

22. Êtes-vous préoccupé par les effets secondaires des transfusions plaquettaires prophylactiques ?

- ○ Toujours
- ○ Parfois
- ○ Rarement
- ○ Jamais

Si oui:

21 b) En général : sur une échelle de 1 à 10, comment evaluez vous votre preoccupation par les effets secondaires suivants, lors d'une prescription de transfusion de plaquettes?

g) TRALI

h) Infections acquises en soins intensifs
i) Effets sur le système immunitaire
j) Risque de thrombose
k) La surcharge hydrosodée
l) Infections virales ou maladies à prions transmises par la transfusion (hépatite, VIH, maladie de Creutzfeldt-Jakob ou similaire)

pas du tout / 1 2 3 4 5 6 7 8 9 10 / très préoccupé

23. Au cours de vos 10 dernières prescriptions de transfusions plaquettaires, combien de fois étiez-vous sûr de l'indication clinique ?

- ○ A chaque fois
- ○ La plupart du temps
- ○ La moitié du temps
- ○ Parfois
- ○ Aucun
- ○ Je ne sais pas

24. Des directives écrites sur la transfusion de plaquettes aux soins intensifs, basées sur les meilleures pratiques et les avis d'experts, ont été publiées.

a) Lisez-vous ces recommandations ?

- ○ Toujours
- ○ Parfois
- ○ Rarement
- ○ Jamais
- ○ Je ne sais pas

b) Si vous avez lu ces recommandations - appliquez-vous ces recommandations

- ○ Toujours
- ○ Parfois
- ○ Rarement
- ○ Jamais

- ○ Je ne sais pas

PARTIE 4 : TRANSFUSIONS PLAQUETTAIRES CHEZ LES PATIENTS PRESENTANT UNE HEMORRAGIE ACTIVE EN REANIMATION MEDICALE (4 QUESTIONS)

25. En général, quel est pour vous le seuil de transfusion plaquettaire chez les patients thrombopéniques, en soins intensifs, présentant **une hémorragie mineure** (grade I-II de l'OMS ; hémorragie hors SNC ne nécessitant pas de transfusion de globules rouges) ?
    - ○ Je ne prescris pas de transfusions plaquettaires prophylactiques
    - ○ 10 x 10^9/L
    - ○ 20 x 10^9/L
    - ○ 30 x 10^9/L
    - ○ 40 x 10^9/L
    - ○ 50 x 10^9/L
    - ○ 80 x 10^9/L
    - ○ 100 x 10^9/L
    - ○ Autre, veuillez préciser : ____

26. Quels tests de coagulation utilisez-vous systématiquement pour évaluer l'état de la coagulation chez un patient thrombocytopénique présentant **une hémorragie mineure** (OMS I-II ; ne nécessitant pas de transfusion de globules rouges) ? (Plusieurs réponses possibles)
    - ○ Aucune
    - ○ Hémoglobine
    - ○ Numération plaquettaire
    - ○ Temps de prothrombine (PT) / Rapport international normalisé (INR)
    - ○ Temps de thromboplastine partielle activée (TCA)
    - ○ Fibrinogène
    - ○ Antithrombine
    - ○ Thromboélastométrie rotationnelle (ROTEM)/Thromboélastographie (TEG)
    - ○ Agrégométrie électrique multiple (AEM) - Agrégométrie plaquettaire (Multiplate)
    - ○ Autres, veuillez préciser: ____________________________________

27. En général, quel est pour vous le seuil de transfusion plaquettaire chez les patients thrombopéniques, en soins intensifs, présentant **une hémorragie majeure** (grade III-IV de l'OMS ; nécessitant une transfusion de globules rouges) ?

- ○ Je ne prescris pas de transfusions plaquettaires
- ○ 10 x 10^9/L
- ○ 20 x 10^9/L
- ○ 30 x 10^9/L
- ○ 40 x 10^9/L
- ○ 50 x 10^9/L
- ○ 80 x 10^9/L
- ○ 100 x 10^9/L
- ○ Autre, veuillez préciser : ____

28. Quels tests de coagulation prescrivez-vous systématiquement pour évaluer l'état de la coagulation chez un patient thrombocytopénique présentant **une hémorragie majeure** (OMS III-IV ; Hemorragie nécessitant une transfusion de globules rouges) ?
(Plusieurs réponses possibles)

- ○ Aucun
- ○ Hémoglobine
- ○ Numération plaquettaire
- ○ Temps de prothrombine (PT) / Rapport international normalisé (INR)
- ○ Temps de thromboplastine partielle activée (TCA)
- ○ Fibrinogène
- ○ Antithrombine
- ○ Thromboélastométrie rotationnelle (ROTEM)/Thromboélastographie (TEG)
- ○ Agrégométrie électrique multiple (AEM) - Agrégométrie plaquettaire (Multiplate)
- ○ Autres, veuillez préciser: ____

PARTIE 5 : ESSAI SUR LA TRANSFUSION PLAQUETTAIRE (4 questions)

29. Seriez-vous prêt à inclure les patients thrombopeniques dans différentes stratégies de prise en charge dans un futur essai clinique randomisé (ECR) ?

- ○ Oui
- ○ Oui, peut-être
- ○ Probablement pas
- ○ Non

30. Si vous deviez participer à un ECR, quel protocole de transfusion préféreriez-vous ?

- ○ Un protocole à deux niveaux :
  Un seuil de transfusion pour les patients a risque faible/ sans risque de saignement et un seuil pour les patients a risque éleve de saignement ou ayant un saignement actif.
- ○ Un protocole à trois niveaux :
  Un seuil de transfusion pour les patients à risque faible/ sans risque de saignement, un seuil pour les patients à risque élevé de saignement et un seuil pour les patients présentant un saignement actif.
- ○ Autre, veuillez préciser :
  ____
  ____

31. Soins intensifs, si vous deviez participer à un ECR : quel seuil de transfusion de plaquettes (faible versus élevé) recommandiez-vous chez les patients **à faible risque hémorragique** ?

- ○ Pas de transfusion contre 10 x 10^9/L
- ○ Seuil de transfusion de plaquettes de 10 x 10^9/L contre 20 x 10^9/L
- ○ Seuil de transfusion de plaquettes de 10 x 10^9/L contre 30 x 10^9/L
- ○ Seuil de transfusion de plaquettes de 10 x 10^9/L contre 40 x 10^9/L
- ○ Seuil de transfusion de plaquettes de 20 x 10^9/L contre 40 x 10^9/L
- ○ Seuil de transfusion de plaquettes de 20 x 10^9/L contre 50 x 10^9/L
- ○ Autre, veuillez préciser :
  ____

32. En Soins intensifs, si vous deviez participer à un ECR : quel seuil de transfusion de plaquettes (faible versus élevé) recommandiez-vous chez les patients **à haut risque hémorragique** ?

- ○ Je n'inclurais pas les patients présentant un risque élevé d'hémorragie dans un essai
- ○ Seuil de transfusion de plaquettes de 10 x 10^9/L contre 20 x 10^9/L
- ○ Seuil de transfusion de plaquettes de 10 x 10^9/L contre 30 x 10^9/L
- ○ Seuil de transfusion de plaquettes de 10 x 10^9/L contre 40 x 10^9/L
- ○ Seuil de transfusion de plaquettes de 20 x 10^9/L contre 40 x 10^9/L

- ○ Seuil de transfusion de plaquettes de 20 x 10^9/L contre 50 x 10^9/L
- ○ Seuil de transfusion de plaquettes de 30 x 10^9/L contre 50 x 10^9/L
- ○ Autre, veuillez préciser: ____

33. En Soins intensifs, si vous deviez participer à un ECR : quel seuil de transfusion de plaquettes (faible versus élevé) recommandiez-vous chez les patients présentant **une hémorragie active** ?

- ○ Je n'inclurais pas les patients présentant une hémorragie en cours dans un essai.
- ○ Seuil de transfusion de plaquettes de 10 x 10^9/L contre 20 x 10^9/L
- ○ Seuil de transfusion de plaquettes de 10 x 10^9/L contre 30 x 10^9/L
- ○ Seuil de transfusion de plaquettes de 10 x 10^9/L contre 40 x 10^9/L
- ○ Seuil de transfusion de plaquettes de 20 x 10^9/L contre 40 x 10^9/L
- ○ Seuil de transfusion de plaquettes de 20 x 10^9/L contre 50 x 10^9/L
- ○ Seuil de transfusion de plaquettes de 30 x 10^9/L contre 50 x 10^9/L
- ○ Seuil de transfusion de plaquettes de 30 x 10^9/L contre 80 x 10^9/L
- ○ Seuil de transfusion de plaquettes de 50 x 10^9/L contre 80 x 10^9/L
- ○ Autre, veuillez préciser :
  ____

PARTIE 6 : OPINIONS PERSONNELLES DES MEDECINS SUR LES TRANSFUSIONS DE PRODUITS SANGUINS (4 questions)

Veuillez noter que les questions suivantes concernent toutes les transfusions sanguines : les transfusions de globules rouges (GR), de plasma frais congelé (PFC) et de plaquettes.

34 a). Pensez-vous que les produits sanguins sont différents des autres traitements prescrits en réanimation ?

- ○ Oui
- ○ Non

34 b) Si oui, pour quelles raisons ? ____

35. Pensez-vous que votre point de vue et vos expériences en matière de transfusion sanguine sont influencés par la culture (à l'hôpital, dans le service, dans la société) ?

- Oui
  - Veuillez préciser quels éléments de votre culture influencent vos opinions et vos expériences (mots clés) :______________________________________
- Non
- Je ne sais pas

36 a). Certains patients pensent que d'autres éléments inconnus peuvent être transférés par une transfusion sanguine. Pensez-vous que cela soit possible ?

- Oui, certainement
- Oui, peut-être
- Non, mais je ne peux pas l'exclure
- Non, absolument pas
- Je ne sais pas

36 b) Si oui, pouvez-vous donner quelques exemples ?

____

37. Avez-vous, ou l'un de vos proches, déjà reçu une transfusion sanguine ?

- Oui
- Non
- Je ne sais pas
- Je préfère ne pas répondre

PARTIE (7) : DONNEES DEMOGRAPHIQUES

38. Quelle est votre principale spécialité ?

- Médecine intensive et réanimation
- Anesthésiologie
- Hématologie
- Oncologie
- Pulmonologie
- Médecine interne
- Neurologie

- ○ Chirurgie
- ○ Autre, veuillez préciser : ____

39. Depuis combien d'années travaillez-vous en réanimation ?_________________

40. Votre âge ? _____________

41. Votre sexe ?

- ○ Homme
- ○ Femme
- ○ Non-binaire
- ○ Autre
- ○ Préfère ne pas répondre

42. Avez-vous des croyances religieuses ?

- ○ Oui, et je suis pratiquant
- ○ Oui, mais je ne suis pas pratiquant
- ○ Je ne sais pas
- ○ Non
- ○ Préfère ne pas répondre

43. Tout commentaire ou réflexion supplémentaire que vous souhaiteriez ajouter :
____
____
____

C'ÉTAIT LA DERNIÈRE QUESTION. MERCI BEAUCOUP POUR VOTRE AIDE !

INVITATION LETTER

Dear intensive care colleague,

We would be very grateful if you would take a few minutes to complete the attached survey about thrombocytopenia and platelet transfusions in the intensive care unit. The survey will take about 10 minutes to complete, and the data collection activities will be conducted anonymously.

The survey consists of seven parts and covers several aspects of platelet and blood transfusions. Please note that we will consider the completion of the survey link as informed consent on your part. We will send you the survey results via email once the survey is closed and the results are analysed.

Your response is extremely important. Please feel free to call or email us if you have any questions, concerns or feedback on the survey. We have provided a short rationale for the study below.

Thank you!

Kind regards,

On behalf of the Nine-I Research Group,

Lene Russell, MD, PhD

Copenhagen University Hospital Gentofte
E-mail: lene.russell@mail.dk
Phone: +453867 2561, Mobile: +45 60213066

Background and rationale for the survey

Thrombocytopenia is common in the intensive care unit (ICU)[1–3] and increases the risk of major bleeding complications. [4] Thrombocytopenia is seen in different subpopulations, such as trauma patients[5], cardiac ICU patients,[6,7] patients with sepsis and in patients with cancer. Therefore, the

pathogenesis behind thrombocytopenia may differ among ICU patients. Platelet transfusions are often used to try to correct thrombocytopenia in ICU patients[8] in order to prevent or treat bleeding,[9,10] however, evidence is still scarce within this area, and specific guidelines for ICU subpopulations are often lacking. Therefore, the decision to transfuse platelets is often left to the treating physician. We are performing this study as part of a larger platelet transfusion research program to identify and describe the preferences of ICU physicians regarding platelet transfusions and explore which variables influence their decisions.

With this survey, we aim to investigate common practices and establish whether variations exist and the extent to which they may be present. We sincerely hope that the information we will receive through this survey will help us design future research on platelet transfusions in the ICU and thereby provide the evidence that can help us improve treatment for our patients.

References

1. Williamson DR, Lesur O, Tétrault J-P, Nault V, Pilon D. Thrombocytopenia in the critically ill: prevalence, incidence, risk factors, and clinical outcomes.Can J Anaesth 2013; 60: 641–51.

2. Crowther MA, Cook DJ, Meade MO, Griffith LE, Guyatt GH, Arnold DM, Rabbat CG, Geerts WH, Warkentin TE. Thrombocytopenia in medical-surgical critically ill patients: Prevalence, incidence, and risk factors.J Crit Care 2005; 20: 348–53.

3. Strauss R, Wehler M, Mehler K, Kreutzer D, Koebnick C, Hahn EG. Thrombocytopenia in patients in the medical intensive care unit: bleeding prevalence, transfusion requirements, and outcome.Crit Care Med 2002; 30: 1765–71.

4. Cook RJ, Sigouin CS, Heddle NM, Webert KE. The risk of bleeding in thrombocytopenic patienyts with acute myeloid leukemia.Haematologica 2006; 91: 1530–7.

5. Hanes SD, Quarles DA, Boucher B. Incidence and risk factors of thrombocytopenia in critically ill trauma patients.Ann Pharmacother 1997; 3: 285–9.

6. Selleng S, Selleng K, Wollert HG, Muellejans B, Lietz T, Warkentin TE, Greinacher A. Heparin-induced thrombocytopenia in patients requiring prolonged intensive care unit treatment after cardiopulmonary bypass.J Thromb Haemost 2008; 6: 428–35.

7. Vonderheide RH, Thadhani R, Kuter DJ. Association of thrombocytopenia with the use of intra-aortic balloon pumps.Am J Med 1998; 105: 27–32.

8. Stanworth SJ, Walsh TS, Prescott RJ, Lee RJ, Watson DM, Wyncoll DLA. Thrombocytopenia and platelet transfusion in UK critical care: A multicenter observational study.Transfusion 2013; 53: 1050–8.

9. McIntyre L, Tinmouth AT, Fergusson D a. Blood component transfusion in critically ill patients.Curr Opin Crit Care 2013; 19: 326–33.

10. Wandt H, Schaefer-Eckart K, Wendelin K, Pilz B, Wilhelm M, Thalheimer M, Mahlknecht U, Ho A, Schaich M, Kramer M, Kaufmann M, Leimer L, Schwerdtfeger R, Conradi R, Dölken G, Klenner A, Hänel M, Herbst R, Junghanss C, Ehninger G. Therapeutic platelet transfusion versus Routine prophylactic transfusion in patients with haematological malignancies: An open-label, multicentre, randomised study.Lancet 2012; 380: 1309–16.

11. Gaur PS, Zimba O, Agarwal V, Gupta L. Reporting Survey Based Studies – a Primer for Authors.J Korean Med Sci 2020; 35: 1–15.

12. Edwards PJ, Roberts I, Clarke MJ, DiGuiseppi C, Wentz R, Kwan I, Cooper R, Felix LM, Pratap S. Methods to increase response to postal and electronic questionnaires.Cochrane Database Syst Rev 2009. doi: 10.1002/14651858.MR000008.pub4

13. Jones TL, Baxter M, Khanduja V. A quick guide to survey research.Ann R Coll Surg Engl 2013; 95: 5–7.

14. De Bruin S, Scheeren TWL, Bakker J, Van Bruggen R, Vlaar APJ. Transfusion practice in the non-bleeding critically ill: An international online survey-the TRACE survey.Crit Care 2019; 23: 1–8.

15. Sharma A, Minh Duc NT, Luu Lam Thang T, Nam NH, Ng SJ, Abbas KS, Huy NT, Marušić A, Paul CL, Kwok J, Karbwang J, de Waure C, Drummond FJ, Kizawa Y, Taal E, Vermeulen J, Lee GHM, Gyedu A, To KG, Verra ML, Jacqz-Aigrain ÉM, Leclercq WKG, Salminen ST, Sherbourne CD, Mintzes B, Lozano S, Tran US, Matsui M, Karamouzian M. A Consensus-Based Checklist for Reporting of Survey Studies (CROSS).J Gen Intern Med 2021; 36: 3179–87.

16. Fincham JE. Response rates and responsiveness for surveys, standards, and the Journal.Am J Pharm Educ 2008; 72: 43.

17. van de Weerdt EK, Peters AL, Goudswaard EJ, Binnekade JM, van Lienden KP, Biemond BJ, Vlaar APJ. The practice of platelet transfusion prior to central venous catheterization in presence of coagulopathy: a national survey among clinicians.Vox Sang 2017; 112: 343–51.

SUPPLEMENTAL MATERIAL: Nine-I

The NINE-I research network

The Nine-I ("Caring for critically ill immunocompromised patients: Multinational research network") is an international research network founded by Professor Elie Azoulay in 2015. It is closely associated with the French study group on critically ill immunocompromised patients (GRRR-OH).

The Nine-I group is meeting annually in Paris, France.

More information can be found at: https://www.grrroh.fr/en/nine-i
